# Clinical, economic and humanistic outcomes of medication therapy management services: A systematic review and meta-analysis

**DOI:** 10.3389/fphar.2023.1143444

**Published:** 2023-04-05

**Authors:** Zhi-Jie Deng, Lin Gui, Jing Chen, Shun-Shun Peng, Yu-Feng Ding, An-Hua Wei

**Affiliations:** Department of Pharmacy, Tongji Hospital, Tongji Medical College, Huazhong University of Science and Technology, Wuhan, China

**Keywords:** medication therapy management, economic, clinical, humanistic, systematic review

## Abstract

**Background:** Medication therapy management (MTM) services is a method that can effectively improve patients’ conditions, but the efficacy of economic and humanistic outcomes remain unclear. This systematic review and meta-analysis aim to use economic, clinical and humanistic outcomes to evaluate the multi-benefits of MTM services.

**Method:** A systematic review and meta-analysis was conducted by retrieving PubMed, EMBASE, the Cochrane Library and ClinicalTrial.gov from the inception to April 2022. There were two reviewers screening the records, extracting the data, and assessing the quality of studies independently.

**Results:** A total of 81 studies with 60,753 participants were included. MTM services were more effective in clinical outcomes with decreasing the rate of readmission (OR: 0.78; 95% CI: 0.73 to 0.83; I^2^ = 56%), emergency department visit (OR: 0.88; 95% CI: 0.81 to 0.96; I^2^ = 32%), adverse drug events (All-cause: OR: 0.68; 95% CI: 0.56 to 0.84; I^2^ = 61%; SAE: OR: 0.51; 95% CI: 0.33 to 0.79; I^2^ = 35%) and drug-related problems (MD: −1.37; 95% CI: −2.24 to −0.5; I^2^ = 95%), reducing the length of stay in hospital (MD: −0.74; 95% CI: −1.37 to −0.13; I^2^ = 70%), while the economic and humanistic outcomes were less effective.

**Conclusion:** Our systematic review and meta-analysis demonstrated that MTM services had great ability to improve patients’ clinical conditions while the efficacy of economic and humanistic outcomes, with some of the outcomes showing high degree of heterogeneity and possible publication bias, required more future studies to provide stronger evidence.

**Systematic Review Registration**: [https://www.crd.york.ac.uk/PROSPERO/display_record.php?RecordID=349050], identifier [CRD42022349050].

## 1 Introduction

Medication therapy management (MTM) services encompass multiple medical services delivered by pharmacists. Since the concept of MTM was first proposed in the 1990s (Hepler and Strand, 1990), this novel and integrated strategy to improve patients’ quality of life attracted widespread attention. The MTM services were strongly supported by the US government to facilitate the development of an integrated healthcare system—known as Fairview Health Services, which helped millions of patients over the years (Burns, 2008). A meta-analysis in 2014 concluded that MTM services helped patients to reduce drug-related problems, decrease healthcare utilities, and cut down the cost of healthcare, while the evidence supporting the improvement in health outcomes was insufficient (Viswanathan et al., 2015). This meta-analysis investigated the efficacy of MTM services. The results indicated that the development of MTM services was hindered by the lack of strong evidence and suggested the need for comprehensive and rigorous studies in the future.

Economic, Clinical and Humanistic Outcomes (ECHO) is a model used to provide an omnidirectional view of medications and medical interventions (Reeder, 1995). It depicts the importance of pharmaceutical services combined with traditional clinical outcomes based on more contemporary measures of economic efficiency and quality (Kozma et al., 1993). According to the model, economic, clinical and humanistic outcomes are used to comprehensively analyze the efficacy of medical intervention. Although several studies comprehensively evaluated patient’s burden and medical interventions using the ECHO model, such as refill adherence measures and STOPP/START criteria (Reeder et al., 2000; Jeremy et al., 2011; Hill-Taylor et al., 2013; Zhao et al., 2019; Chua et al., 2020), few studies evaluated the efficacy of MTM services with full ECHO model. Despite descriptive studies demonstrating that MTM services improved patients’ clinical conditions, quality of life and potentially reduced healthcare disparities (Bunting and Cranor, 2006; Shrestha et al., 2022), studies analyzed the three parts of ECHO model in one assessment was little (Singhal et al., 1999; Cheng et al., 2013; Johnson et al., 2018). The incompleteness of the analysis may result in superficial conclusions and less precise outcomes.

Leveraging the ECHO model, our meta-analysis divided outcomes into economic, clinical and humanistic outcomes to evaluate the efficacy of MTM services. The goal of our study was to elucidate the benefits of MTM services and to provide robust evidence supporting the efficacy of MTM services.

## 2 Methods

This systematic review and meta-analysis followed the Preferred Reporting Items for Systematic Reviews and Meta-analyses (PRISMA) 2020 statement (Page et al., 2021). The study protocol (Deng et al., 2022) was registered on PROSPERO 2022 (Registered ID: CRD42022349050).

### 2.1 Database search

MTM services-related studies were retrieved systematically from several mainstream databases and websites (PubMed, The Cochrane Library, Embase and ClinicalTrial.gov) using comprehensive search strategies (Table 1). To ensure compatibility with the requirements of each database, one experienced researcher (WAH) reviewed and refined the search terms. The main key words used in search terms included: “medication therapy management,” “MTM,” “medication reconciliation” and “drug therapy management”. Two researchers (PSS and CJ) conducted the search independently. The final search was conducted on 16 April 2022.

**TABLE 1:** Search terms for databases.

Database	Search terms
PubMed	(“medication therapy management”[MeSH Terms] OR “medication therapy management”[MeSH Terms] OR “medication reconciliation”[MeSH Terms] OR “drug therapy management”[Title/Abstract] OR “medication therapy management”[Title/Abstract] OR “medication reconciliation”[Title/Abstract]) AND {[clinicaltrial(Filter) OR randomizedcontrolledtrial(Filter) OR observationalstudy(Filter)] AND [2014/1/10:2022/4/16(pdat)]}
	Filters applied: Clinical Trial, Randomized Controlled Trial, Observational Study, Humans, English, from 2014/1/10—2022/04/16.
The Cochrane Library	#1 MeSH descriptor: [Medication Therapy Management] explode all trees
	#2 MeSH descriptor: [Medication Reconciliation] explode all trees
	#3 (“medication therapy management” or “MTM” or “drug therapy management” or “medication reconciliation”):ti,ab,kw
	#4 #1 OR #2 OR #3
Embase	(“medication therapy management”/exp OR “medication therapy management” OR “medication reconciliation”/exp OR “medication reconciliation”) AND ([controlled clinical trial]/lim OR [randomized controlled trial]/lim OR “cohort analysis”/de OR “prospective study”/de OR “retrospective study”/de) AND [article]/lim AND [humans]/lim AND [english]/lim
ClinicalTrials.gov	(“medication therapy management” OR “Medication Reconciliation” OR “pharmaceutical case management” OR “drug therapy management” OR “drug therapy problem” OR “drug therapy problems” OR “medicine management” OR “medicines management”) AND AREA[ResultsFirstSubmitDate] NOT MISSING | Available, Completed Studies | medication therapy management | Start date from 01/10/2014 to 04/16/2023

### 2.2 Inclusion and exclusion criteria

Studies included met the following criteria:

1) Predesigned of studies including RCTs and non-randomized studies.
2) Participants: Adults requiring MTM services.
3) Interventions: Containing MTM services administered by pharmacists.
4) Outcomes: At least one of the outcomes we interested in should be reported in the study, including clinical, economic, and humanistic outcomes.

Studies with following criteria were excluded:

1) Studies without control group.
2) Studies published in languages other than English.
3) Animal experiments.
4) Pilot studies and feasibility studies.
5) Studies comparing the efficacy between different MTM interventions.

### 2.3 Definition of outcomes

Basing on the definition from ECHO model for the clinical, economic and humanistic outcomes (Cheng et al., 2013; Shrestha et al., 2022; Gunter., 2023), the definition of outcomes included in our study was confirmed.

Clinical outcomes: Clinical outcomes were defined as medical events that occurred as a result of MTM services, including rate of readmission, emergency department visit, mortality, adverse drug events (including all-cause and serious adverse events), and so on.

Economic outcomes: Based on the definitions of direct and indirect cost in ECHO model, we defined the economic outcomes as measures of medical resource utilization such as hospitalization and medication costs. Indirect costs, such as evaluations of reduced productivity and lost work days, could be assessed if adequate studies results included in.

Humanistic outcomes: Humanistic outcomes were defined as the consequences of disease or treatment on patient functional status or quality of life, including physical function, social function, general health and wellbeing, and life satisfaction. Consequently, our study included life quality scales and evaluations of adherence as part of our assessment of humanistic outcomes.

### 2.4 Definition of intervention and comparison

Intervention: To be eligible for inclusion criteria in our meta-analysis, studies needed to involve the administration of MTM services. To avoid omission of studies and based on the definition of MTM services, medication reconciliation and drug therapy management were also included.

Comparison: The control interventions consisted of usual care or other standard therapies depending on the participants’ diseases. These usual care and standard therapies did not involve any additional medication administration or other therapies when compared with the intervention arm.

### 2.5 Study selection and data extraction

Two researchers (DZJ and GL) independently assessed the studies with eligibility criteria. The data of all included studies were extracted according to a predesigned format by two independent researchers. Any deviation from this approach was resolved by consensus or consultation with a third expert. The following data were extracted: 1) basic information (title, first author, year of publication, type of study, and location of study); 2) study population (age, sample size, detailed description of participants, and diseases); 3) details of interventions and comparison; 4) outcomes and 5) the length of follow-up time.

### 2.6 Quality assessment

The risk of bias of RCTs and non-randomized studies was evaluated by two independent researchers using Risk of Bias 2 tool (Hastert and Denni, 2003; Sterne et al., 2019) and ROBINS-I tool (Sterne et al., 2016), as recommended by Cochrane Handbook for Systematic Reviews of Interventions (version 6.3) (Cochrane, 2022), respectively. Any disagreement was resolved by consensus or consultation with a third expert.

### 2.7 Statistical analysis

All the data were imported and analyzed by RevMan (ver. 5.4). Outcomes including 3 studies or above were synthesized as the results. We used odds ratios (ORs) and 95% confidence intervals (CIs) to measure dichotomous variables, and mean differences (MDs) with 95% CIs for continuous variables. To synthesize the results with different scales involving the same outcomes, such as EQ-5D-3L and 5L, SF-12 and 36, and potential huge variance of unit in the cost in different studies, standard mean differences (SMDs) were calculated as effect measures to eliminate possible discrepancies in different scales. All the outcomes were applied with random-effects model. Heterogeneity was calculated using Chi-squared and I^2^. Results with an I^2^ value greater than 50% were deemed to have a high potential for heterogeneity.

Subgroup analysis was performed to detect the sources of heterogeneity. Type of studies (RCTs/No-randomized studies), district of studies (US/Non-US), and type of diseases (chronic disease/non-chronic disease/undefined) were predetermined as subgroups to analyze the outcomes. Additionally, the robustness of results was evaluated *via* sensitivity analysis—using the method that eliminated one study in the outcome at a time to synthesize the results of remaining studies. Publication bias was assessed *via* funnel plots and Egger’s test using RevMan (ver. 5.4) and STATA (ver. 16), respectively.

## 3 Results

### 3.1 Study selection

**FIGURE 1:**
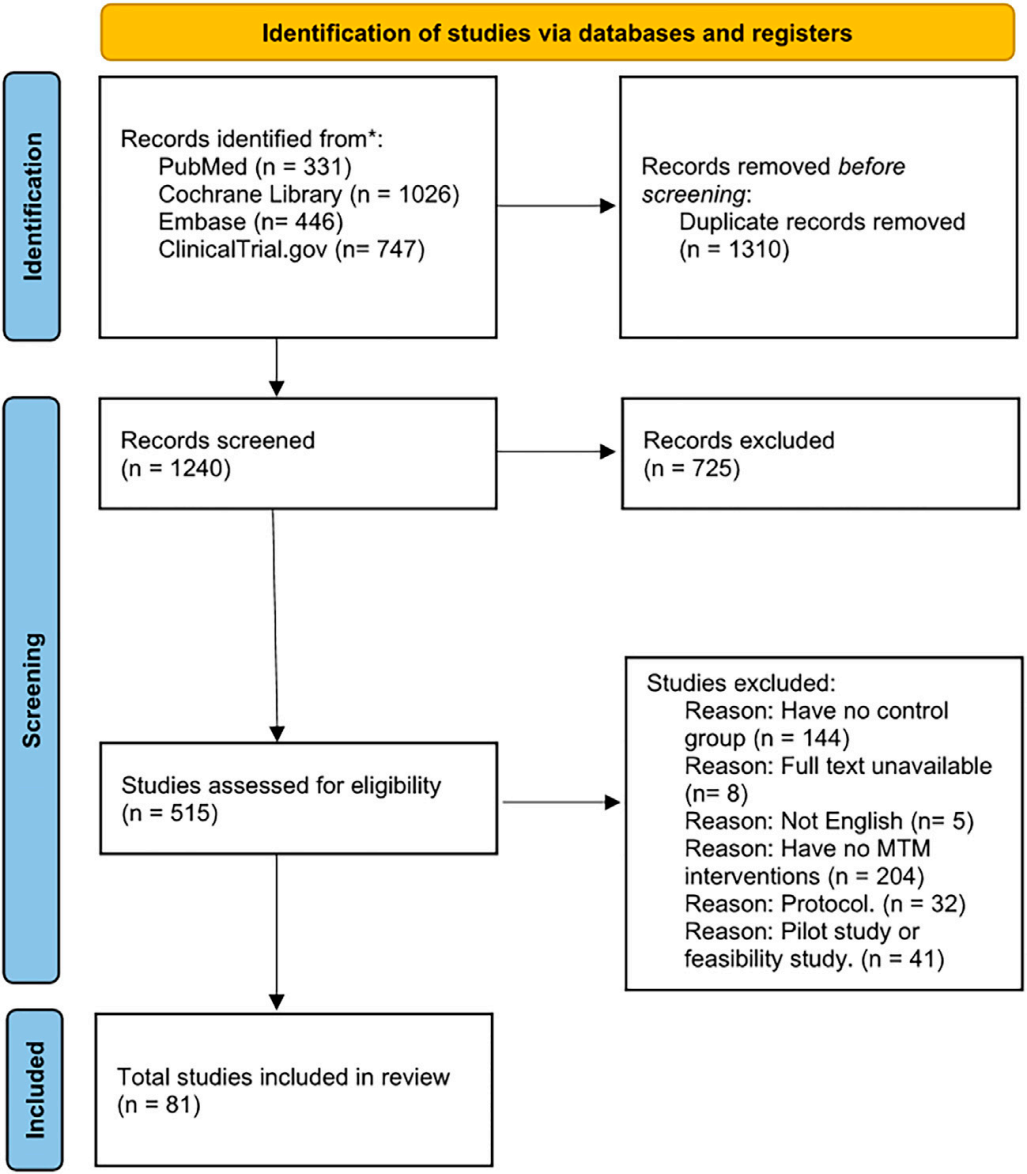
PRISMA flow diagram.

A PRISMA flow diagram was used to illustrate our process in study selection (Figure 1). A total of 2,550 records were identified in our initial search with comprehensive search strategies (Table 1). Finally, our quantitative analysis encompassed 81 studies, consisting of 66 RCTs and 15 non-randomized studies (included 10 NRCTs and 5 cohort studies). These studies were conducted across various countries and covered a range of diseases.

### 3.2 Study characteristics

According to the baseline table, 81 studies involved a total of 60,753 participants, with 29,587 in the intervention group and 31,166 in the control group. Aside from 30 studies conducted in the US, the remaining studies were conducted across various countries such as China, Japan, Australia, and England. In summary, 25 studies were conducted in Europe, 13 in the Aisa and 13 in other locations. This distribution of location roughly showed an overview of development of MTM services in the world, which means the US and Europe was advanced to other countries. Regarding research type, 66 studies were designed as RCT, 15 as non-randomized studies (including 10 NRCTs and 5 cohort studies). Furthermore, among the included studies, 16 studies distinctly focused on patients with chronic diseases, 5 studies included non-chronic disease patients, and 30 studies included patients with no specific definition of diseases. These findings highlight the need to pay more attention to non-chronic diseases and emphasize that MTM services could benefit various kinds of patients, not only those with chronic diseases. Besides, the follow-up time of these studies varied from 30 to 780 days, indicating that MTM services could cover both short-term and long-term care. A baseline table was formulated for the detail characteristics of included studies (Supplementary Table S1).

**FIGURE 2:**
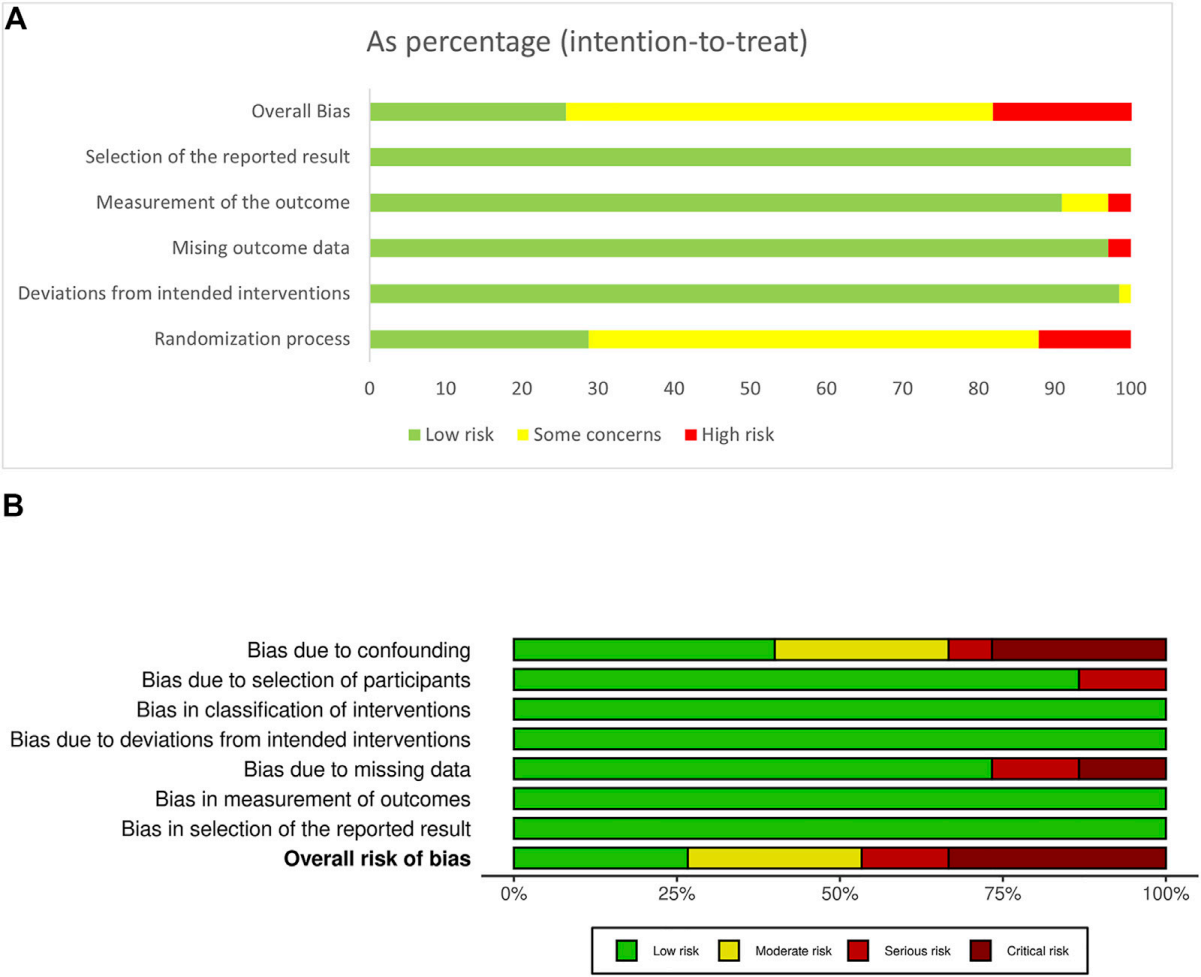
Summaries of risk of bias assessment. **(A)** Risk of Bias 2; **(B)** ROBINS-I.

**FIGURE 3:**
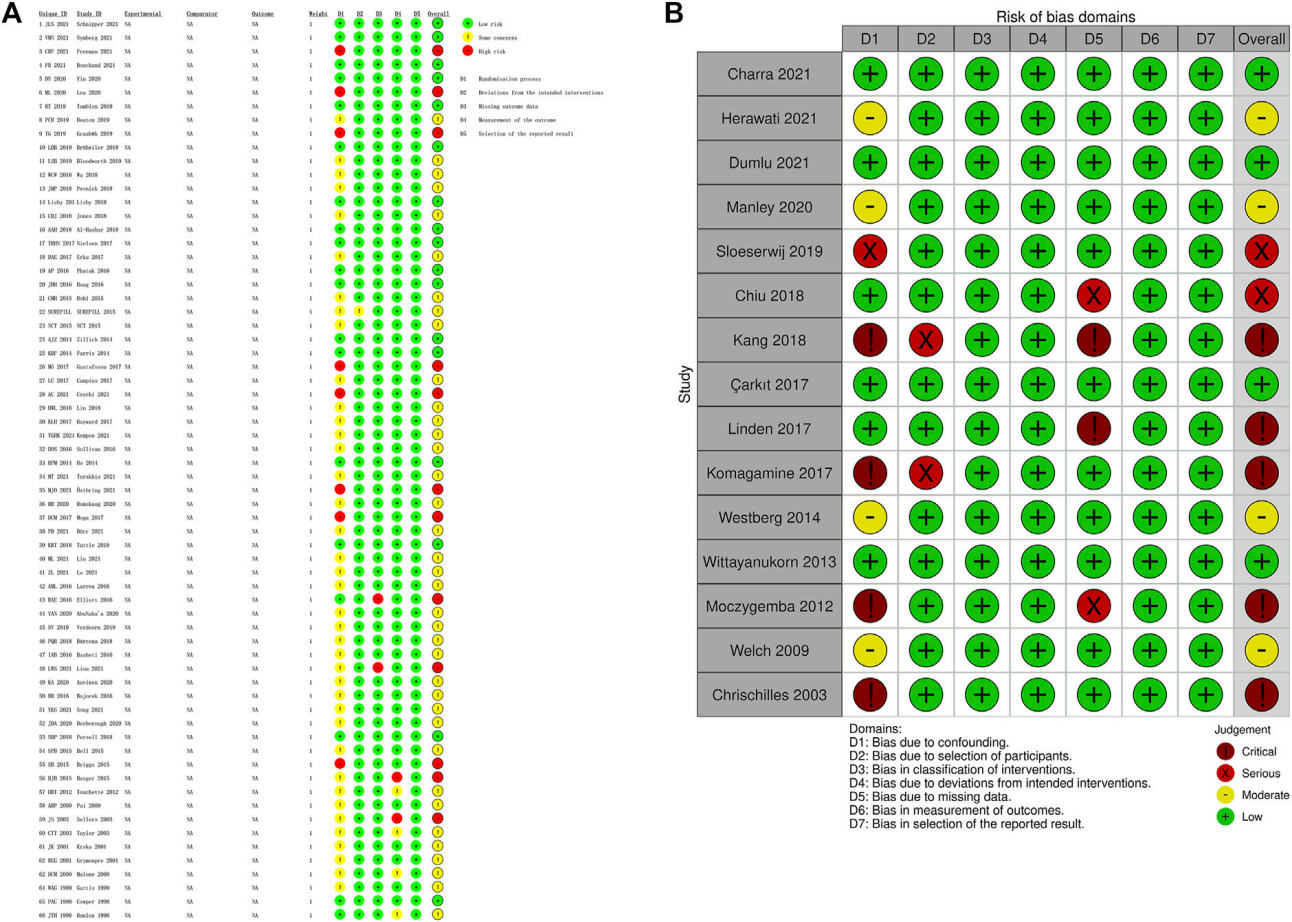
Details of risk of bias assessment. **(A)** Risk of Bias 2; **(B)** ROBINS-I.

The evaluation of risks of bias showed that RCTs had lower risks of bias than non-randomized studies (Figures 2, 3). In RCT studies, the most critical factor leading to bias was randomization process with 28 studies having some concern and 7 having high risk in this domain. In the results of ROBINS-I, confounding had a higher risk of bias than other domains, indicating that many of the included non-randomized studies had less control over confounding factors.

### 3.3 Clinical outcomes

**FIGURE 4:**
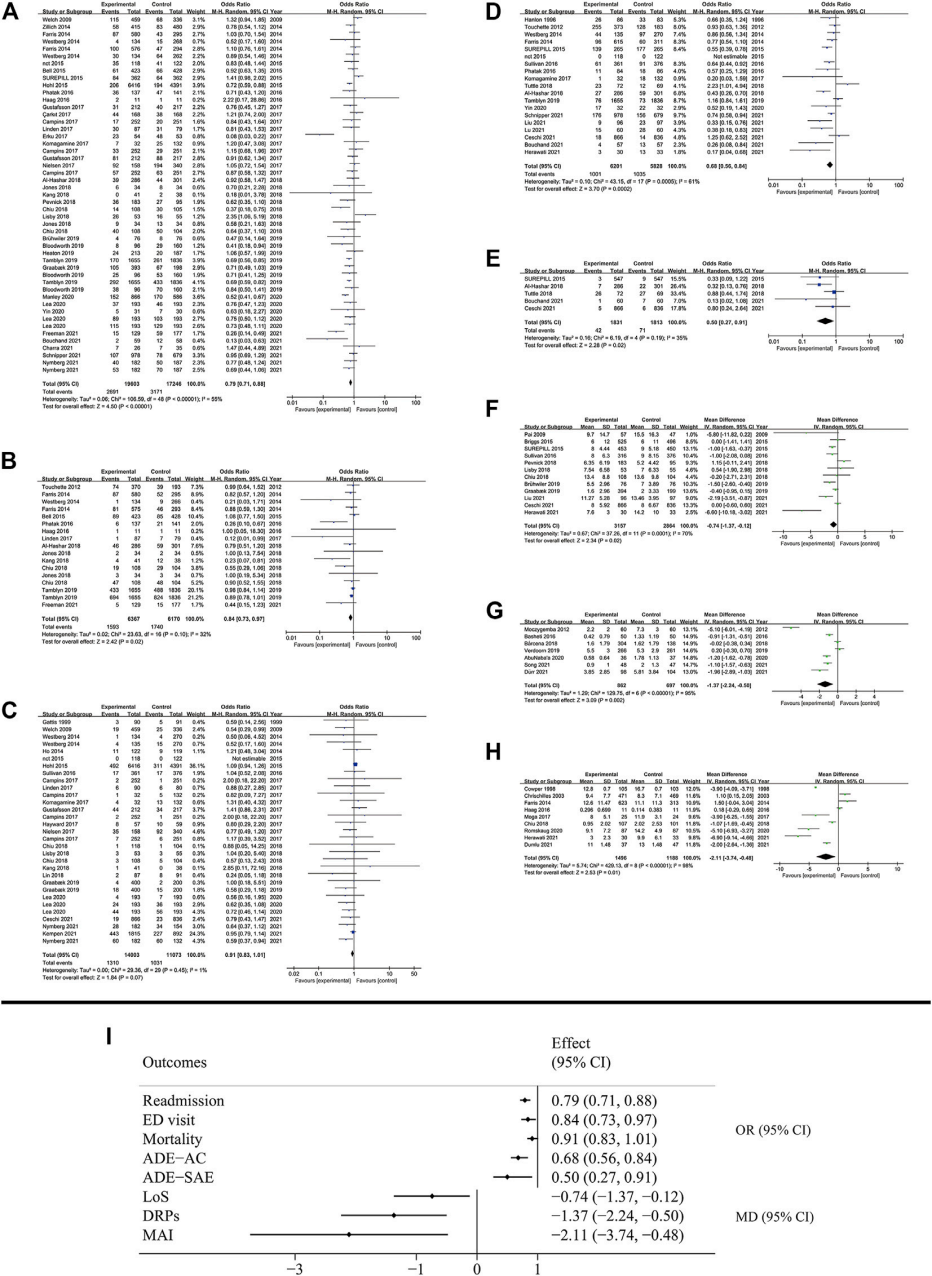
Results of clinical outcomes. **(A)** Readmission; **(B)** ED visit; **(C)** Mortality; **(D)** All cause ADE; **(E)** SAE; **(F)** LoS; **(G)** DRPs; **(H)** MAI; **(I)** Summary of clinical outcomes.

Seven clinical outcomes were analyzed including: rate of readmission, emergency department (ED) visit, mortality, adverse drug event (ADE) which included all-cause ADE and serious adverse event (SAE), the length of stay (LoS) in hospital, drug-related problems (DRPs) and medication appropriateness index (MAI) (Figure 4). Most outcomes demonstrated significant improvement in patient condition with MTM services, though some of them showed high degree of heterogeneity (Figure 2). MTM services effectively reduced the rate of readmission (OR: 0.77; 95% CI: 0.73 to 0.82; I^2^ = 55%), ED visit (OR: 0.88; 95% CI: 0.81 to 0.96; I^2^ = 32%), ADE (All-cause: OR: 0.68; 95% CI: 0.56 to 0.84; I^2^ = 61%; SAE: OR: 0.51; 95% CI: 0.33 to 0.79; I^2^ = 35%), the number of DRPs (MD: −1.37; 95% CI: −2.24 to −0.5; I^2^ = 95%) and MAI (MD: −2.11; 95% CI: −3.74 to −0.48; I^2^ = 98%). Also, the LoS in hospital was significantly shortened (MD: −0.74; 95% CI: −1.37 to −0.13; I^2^ = 70%). However, the mortality rate (OR: 0.91; 95% CI: 0.83 to 1.01; I^2^ = 1%) was not affected by MTM services. The subgroup analysis showed that the disease type was associated with a high possibility of high heterogeneity. For example, based on the results of readmission, three groups of diseases showed huge heterogeneity (chronic disease: OR: 0.82; 95% CI: 0.72 to 0.93; I^2^ = 70%; non-chronic disease: OR: 0.96; 95% CI: 0.69 to 1.34; I^2^ = 0%; undefined: OR: 0.75; 95% CI: 0.7 to 0.81; I^2^ = 41%). All the results of subgroup analysis are presented in Supplementary Figures S16–S39.

### 3.4 Economic outcomes

**FIGURE 5:**
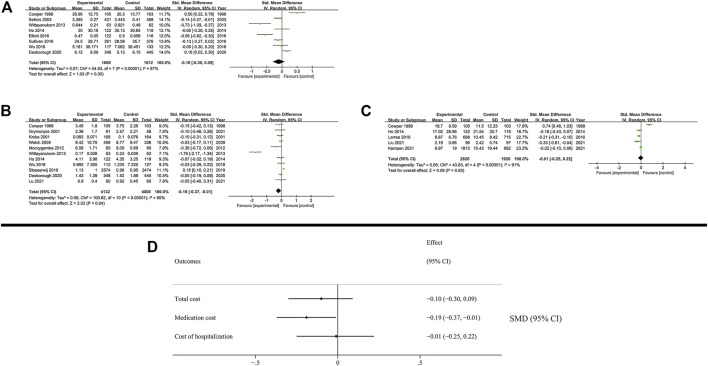
Results of economic outcomes. **(A)** Total cost; **(B)** Medication cost; **(C)** Cost of hospitalization **(D)** Summary of economic outcomes.

Our results showed that the total cost (SMD: −0.1; 95% CI: −0.3 to 0.09; I^2^ = 87%) and the cost of hospitalization (SMD: −0.01; 95% CI: −0.25 to 0.22; I^2^ = 91%) were not reduced, while the medication cost was significantly decreased (SMD: −0.19; 95% CI: −0.37 to −0.01; I^2^ = 90%) (Figure 5). District of study was inferred as a high possibility source of heterogeneity in medication cost. (US: SMD: −0.37; 95% CI: −0.74 to 0.00; I^2^ = 92%; Non-US: SMD: 0.00; 95% CI: −0.14 to 0.15; I^2^ = 69%). The results of subgroup analysis are presented in the Supplementary Figures S40–S48.

### 3.5 Humanistic outcomes

Humanistic outcomes included quality of life and patient’s adherence (Figure 6). The subgroup analysis is presented in the Supplementary Figures S49–S60.

**FIGURE 6:**
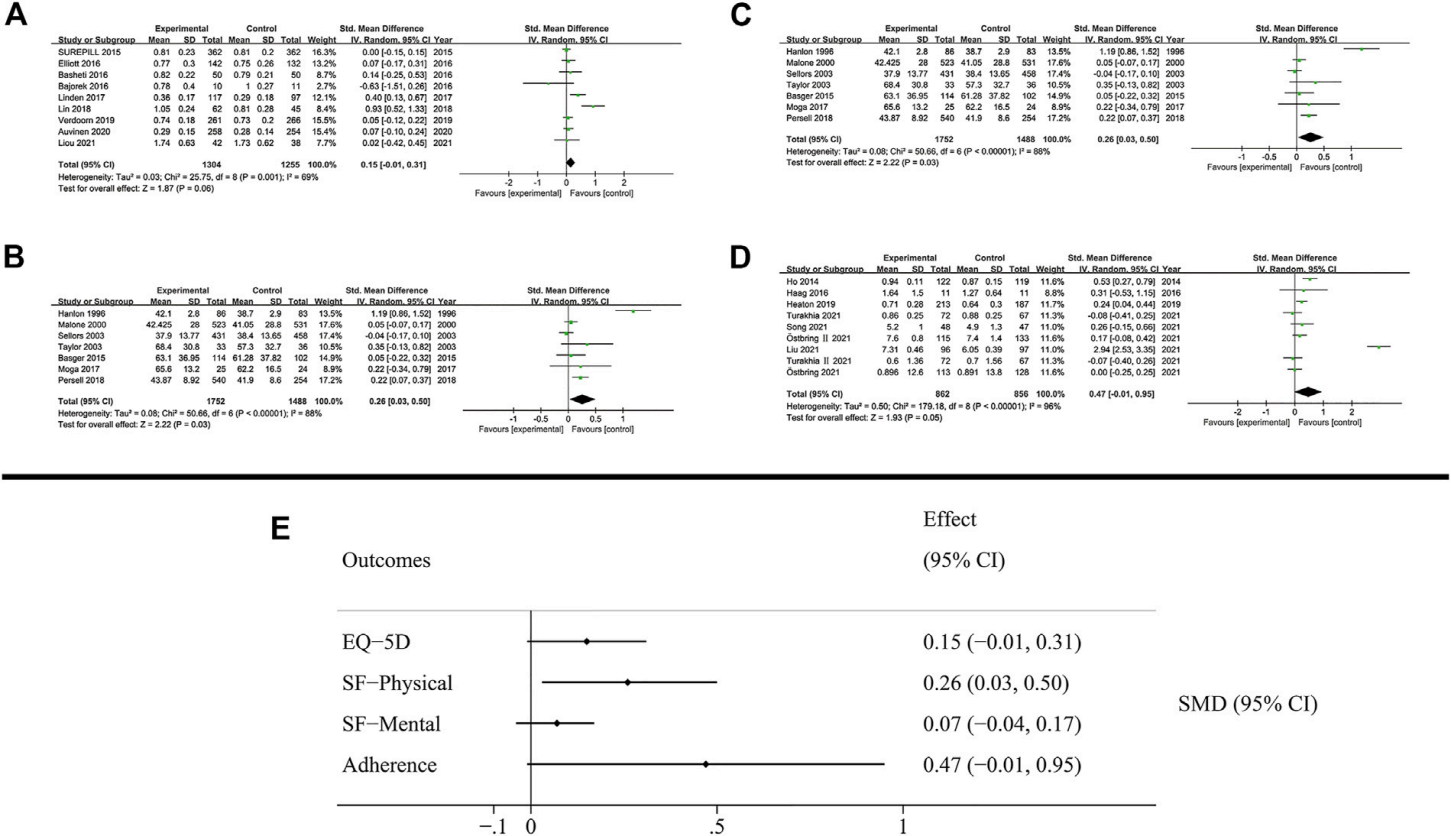
Results of humanistic outcomes. **(A)** EQ-5D; **(B)** SF-Physical outcomes; **(C)** SF-Mental outcomes; **(D)** Adherence; **(E)** Summary of humanistic outcomes.

The quality of life was measured using EQ-5D (3L and 5L) and SF (12D and 36D) scales. The patient’s quality of life based on EQ-5D was not increased by MTM services (SMD: 0.15; 95% CI: −0.01 to 0.31; I^2^ = 69%). The SF scale evaluated the quality of life based on physical and mental outcomes. Notably, MTM services effectively improved physical outcomes (SMD: 0.26; 95% CI: 0.03 to 0.5; I^2^ = 88%), while negligible effect was observed in mental outcomes (SMD: 0.05; 95% CI: −0.02 to 0.12; I^2^ = 43%).

In the analysis of patient’s adherence, the proportion of days covered of medication and the MMAS8 scale were both used as the method to evaluate patient’s adherence in medication administration. The results showed that MTM services had no effect on promoting adherence during the therapy (SMD: 0.47; 95% CI: −0.01 to 0.95; I^2^ = 96%).

### 3.6 Publication bias and sensitivity analysis

Publication bias was assessed by funnel plots and Egger’s test respectively. And according to the Egger’s test, ED visit and medication cost may have the potential of publication bias. Funnel plots were presented in Supplementary Figures S61-S75 and results of Egger’s test were in Supplementary Table S2.

The results of sensitivity analysis indicated that some outcomes, such as mortality, adherence, MAI, medication cost and cost of hospitalization, might have problems of robustness of results. For example, in results of cost of hospitalization, the elimination of Cowper et al.’s (Cowper et al., 1998) study changed the result from negative to positive. However, this change might be attributed to the old year of study. All the results of sensitivity analysis are presented in the Supplementary Figures S76–S90.

## 4 Discussion

Our meta-analysis evaluated the impact of MTM services with ECHO model on multiple outcomes in one assessment. The pooled analysis of 81 studies demonstrated the significant clinical efficacy of MTM services. However, due to limited study numbers and high degree of heterogeneity brought by methodological variations in measuring the economic and humanistic outcomes, the efficacy in economic and humanistic outcomes requires further investigation through additional studies.

### 4.1 Clinical outcomes

Collectively, our meta-analysis demonstrated that MTM services were effective in improving clinical outcomes by decreasing the risks of readmission, ED visit after discharge, lowering the rate of ADE, the numbers of DRPs, shortening the LoS in the hospital and lowering the score of MAI. These results of clinical outcomes were consistent with the results of previous meta-analysis in 2014 (Viswanathan et al., 2015). Other studies also confirmed the clinical efficacy of MTM services, like reducing return visits to emergency department (Hayes et al., 2012) and improving short-term outcomes of patients with diabetes and cardiovascular conditions (Babar et al., 2018). Further, data from Minnesota’s Fairview Health System revealed that about 85% of patients had at least 1 DRP, while the health conditions of 55% of patients improved after receiving comprehensive medication management (McFarland et al., 2021). Despite the remarkable efficacy of MTM services in improving patients’ near-term conditions, the effectiveness of MTM services at usance, as the result of mortality in our meta-analysis showed, was not significant. Some of RCTs and reviews attributed the unsignificant results of reducing the rate of mortality to the inadequate follow-up time (Christensen and Lundh, 2016; Lea et al., 2020) while our meta-analysis, which was larger than previous studies, included more studies with a follow-up time greater than 1 year, yielded the same non-significant results. The primary objective of MTM services was to facilitate patients’ self-managing their health conditions, rather than directly curing the disease and extending their lifetime expectancy. Consequently, the MTM services were more effective in improving patients’ near-term conditions, with limited long-term effects beyond a year.

### 4.2 Economic outcomes

In terms of economic outcomes, the MTM services reduced the need for additional medication, thereby decreasing the medication cost directly. The intervention reduced ADEs, facilitated adherence to evidence-based guidelines, timely implemented medication therapies, and increased the use of cost-effective drug therapies (Morgan et al., 2018). MTM services significantly reduced the rates of readmission and ED visits, which benefit-cost ratio of MTM services was reported that ranged from 2.1:1 to 2.6:1 by another review (Moczygemba et al., 2019). A prospective study demonstrated that MTM services contributed to a 57.9% reduction in facility costs and an 11.1% reduction in professional claims, despite a 19.7% increase in prescription drug expenditures (Isetts et al., 2008). Though many same conclusions were reported to demonstrate the efficacy of saving partial cost by MTM services (Campbell et al., 2018; Ni et al., 2018; Chung et al., 2020; Bezerra et al., 2022), several other costs apart from the medication cost might be increased by delivering MTM services, which diluted the efficacy in reducing total cost.

### 4.3 Humanistic outcomes

The results of humanistic outcomes showed that MTM services were more effective in improving physical outcomes like SF in the physical outcome compared with mental results, such as adherence and SF in the mental outcome. This result indicated that MTM services focused more on the patients’ physical condition and less on the mental condition. Numerous studies had reported similar results, suggesting the need for further research with greater methodological rigor to obtain evidence supporting mental health (Finley et al., 2003; Rubio-Valera et al., 2014; Hattingh et al., 2016; Silva et al., 2018). In contrast to a Cochrane review assessing multiple pharmaceutical interventions in elders and reporting little or slight effects on improving patients’ adherence, appropriateness of polypharmacy and quality of life (Rankin et al., 2018), our meta-analysis demonstrated some efficacy of MTM services in humanistic outcomes. While this Cochrane review focused on elders receiving polypharmacy, our meta-analysis was not restricted to a specific population, which enhances the generalizability of our results. Besides, as a general instrument involving the general population (Rabin and de Charro, 2001), EQ-5D might result in a less precise evaluation of a patient’s condition with a specific disease. A combination of this general instrument and disease-specific scales, such as the global registry of acute coronary events (GRACE) risk score, would be more beneficial for a comprehensive analysis. These considerations highlight the necessity for improved measures to evaluate patients’ quality of life. In adherence outcomes, due to differences in the definition of “adherent” and the lack of diagnostic gold standard, the rate of adherence in a patient with chronic conditions was reported to range between 43% and 78% (Osterberg and Blaschke, 2005) as a result. However, patients receiving multiple pharmaceutical care have a higher rate of adherence, reported to be approximately 72% (DiMatteo, 2004). Thus, despite the results of negative adherence and high levels of heterogeneity in our meta-analysis, the efficacy of MTM services for improving patients’ adherence was undeniable.

### 4.4 Prospects and limitations

Our meta-analysis also provided the idea that more studies need to focus on economic and humanistic outcomes to provide more comprehensively perspective of MTM services. Although MTM services could provide continuous support to patients with chronic conditions for a prolonged period, the identified disparities, according to our results, between the near-term and long-term outcomes of MTM services underscore the need for more targeted and precise research for long-term studies. Based on the results of risks of bias assessment, both RCT studies and non-randomized studies face challenges in controlling bias. To further enhance the quality of evidence generated from future studies, the adoption of the Grading of Recommendations, Assessment, Development and Evaluations (GRADE) framework (Guyatt et al., 2008) could be considered to provide a comprehensive assessment of the evidence quality and ensure the validity of the evidence generated. As MTM services are an intervention designed to guide patients in developing self-management (Burns, 2008), a lot of anthropic factors and confounding factors makes trials more difficulty to control the bias, especially in the non-randomized studies. Though subgroup analysis indicated that there was little heterogeneity in results between RCTs and non-randomized studies, future study design should be more cautious in controlling for bias and clarifying confounding factors to enhance the confidence of study results. The subgroup analysis also reveals the differences in the efficacy of MTM services between the US and other countries. These findings can be further explored to understand the variations in the more developed MTM services system in the US compared to other countries that are relatively new to this field.

Several limitations exist in this meta-analysis. First, despite the best possible search of databases, the omission of studies was inevitable, including grey articles and ongoing clinical trials. Second, though the extensive outcomes including the evaluation of the ECHO model leading to comprehensive conclusions, the meta-analysis is limited by the inadequate number of studies for every outcome analyzed. Several outcomes were predesigned in our protocol, while some of them were eventually excluded in our meta-analysis after retrieving studies. Third, the results of sensitivity analysis and publication bias showed that several outcomes, like cost of hospitalization, medication cost, mortality, and MAI, involved unstable results or publication bias. Finally, although a subgroup analysis was performed to identify the sources of heterogeneity, it was difficult to explain all the reasons underlying the high degree of heterogeneity.

The RCTs, with more rigorous inclusion and exclusion criteria, could generate more robust findings than non-randomized studies, while these restrictions limited generalizability of RCT results to real-world settings compared to non-randomized studies. And some studies reported that these two types of studies might induce the heterogeneity (Mekonnen et al., 2016; Ahmed et al., 2022). Based on these facts, we supposed that if both types of studies showed consistent tendencies of results in terms of specific outcomes, the combination analysis of these two types of studies would be more considered valid. The second one of possible source of heterogeneity was the type of diseases. Subgroup analysis revealed that MTM services were more effective in studies including patients with chronic diseases. This result was consistent with the results of other studies assessing chronic conditions (Hohmeier et al., 2019; Thompson et al., 2020; Wang et al., 2021). But limited studies showed the efficacy of MTM services in patients with non-chronic conditions, like cancer (Dürr et al., 2021) and acute coronary syndrome (Kang et al., 2018). These results showed possibility of heterogeneity in the type of diseases and indicated the need for additional evidence to support the efficacy of MTM services for managing patients with non-chronic disease. The development level and different work model of MTM services system in different countries was another main factor being considered. MTM services in the US, for instance, developed over the years and the fees were covered by American healthcare (H.R.1–108th Congress (2003-2004): Medicare Prescription Drug, Improvement, and Modernization Act of 2003, 2019), while other countries such as China offered similar MTM services free of charge. These discrepancies between different countries led to large disparities in medical expenses and healthcare use (Khalil et al., 2017; Maria et al., 2021), resulting in a high degree of heterogeneity. As a review resulted that the impact of the payment model on healthcare spending and utilization was found to vary considerably across studies (Agarwal et al., 2020), this difference might also be a source of heterogeneity. Furthermore, it is important to acknowledge that in addition to the factors mentioned, there might be other potential sources of heterogeneity such as variations between urban and rural patients, differences between those with medical insurance and those without, and variances in MTM services provided by a medical team versus those solely delivered by pharmacists. However, due to limited studies including these facts, the conduction of these analysis required more future studies focusing on them.

## 5 Conclusion

The results of our meta-analysis provide some evidence demonstrating the effectiveness of MTM services across multiple outcome measures. The findings suggest that MTM services are beneficial in improving short-term patient outcomes. However, due to high heterogeneity and unstable results observed in some economic and humanistic outcomes, additional studies are necessary to establish their efficacy in reducing patient costs and enhancing their quality of life. Further evaluation and standardization of economic outcome measures are necessary for a more effective analysis. Moreover, the humanistic outcomes should underscore the importance of mental healthcare in promoting patients’ overall wellbeing.

## Data Availability

The raw data supporting the conclusion of this article will be made available by the authors, without undue reservation.

## References

[B1] Agarwal R. Liao J. M. Gupta A. Navathe A. S. (2020). The impact of bundled payment on health care spending, utilization, and quality: A systematic review. Health Aff. 39, 50–57. 10.1377/hlthaff.2019.00784 31905061

[B2] Ahmed A. Abdulelah Dujaili J. Rehman I. U. Chuah L. H. Hashmi F. K. Awaisu A. (2022). Effect of pharmacist care on clinical outcomes among people living with HIV/aids: A systematic review and meta-analysis. Res. Soc. Adm. Pharm. 18, 2962–2980. 10.1016/j.sapharm.2021.07.020 34353754

[B3] Babar Z. U. Kousar R. Murtaza G. Azhar S. Khan S. A. Curley L. (2018). Randomized controlled trials covering pharmaceutical care and medicines management: A systematic review of literature. Res. Soc. Adm. Pharm. 14 (6), 521–539. 10.1016/j.sapharm.2017.06.008 28651923

[B4] Bezerra H. S. Brasileiro Costa A. L. Pinto R. S. Ernesto de Resende P. Martins de Freitas G. R. (2022). Economic impact of pharmaceutical services on polymedicated patients: A systematic review. Res. Soc. Adm. Pharm. 18 (9), 3492–3500. 10.1016/j.sapharm.2022.03.005 35337757

[B5] Bunting B. A. Cranor C. W. (2006). The asheville project: Long-term clinical, humanistic, and economic outcomes of a community-based medication therapy management program for asthma. J. Am. Pharm. Assoc. 46 (2), 133–147. 10.1331/154434506776180658 16602223

[B6] Burns A. (2008). Medication therapy management in pharmacy practice: Core elements of an MTM service model (version 2.0). J. Am. Pharm. Assoc. 48 (3), 341–353. 10.1331/japha.2008.08514 18595820

[B7] Campbell A. M. Coley K. C. Corbo J. M. DeLellis T. M. Joseph M. Thorpe C. T. (2018). Pharmacist-led drug therapy problem management in an interprofessional geriatric care continuum: A subset of the pivots group. Am. Health Drug Benefits 11 (9), 469–478. 30746018 PMC6322592

[B8] Cheng Y. Raisch D. W. Borrego M. E. Gupchup G. V. (2013). Economic, clinical, and humanistic outcomes (ECHOs) of pharmaceutical care services for minority patients: A literature review. Res. Soc. Adm. Pharm. 9 (3), 311–329. 10.1016/j.sapharm.2012.05.004 22835704

[B9] Christensen M. Lundh A. (2016). Medication review in hospitalised patients to reduce morbidity and mortality. Cochrane Database Syst. Rev. 2, Cd008986. 10.1002/14651858.CD008986.pub3 26895968 PMC7119455

[B10] Chua B. Morgan J. Yap K. Z. (2020). Refill adherence measures and its association with economic, clinical, and humanistic outcomes among pediatric patients: A systematic review. Int. J. Environ. Res. Public Health 17 (6), 2133. 10.3390/ijerph17062133 32210111 PMC7142643

[B11] Chung T. H. Hernandez R. J. Libaud-Moal A. Nguyen L. K. Lal L. S. Swint J. M. (2020). The evaluation of comprehensive medication management for chronic diseases in primary care clinics, a Texas delivery system reform incentive payment program. BMC Health Serv. Res. 20(1), 671. 671, 10.1186/s12913-020-05537-3 32690015 PMC7372764

[B12] Cochrane (2022). Cochrane Handbook for systematic reviews of interventions Cochrane.org. Available at: https://training.cochrane.org/handbook/current (Assessed 8 30, 2022).

[B13] Cowper P. A. Weinberger M. Hanlon J. T. Landsman P. B. Samsa G. P. Uttech K. M. (1998). The cost-effectiveness of a clinical pharmacist intervention among elderly outpatients. Pharmacotherapy 18, 327–332. 9545151

[B14] Deng Z.-J. Ding Y.-F. Peng S.-S. Wang L. Wei A.-H. (2022). Multiple beneficial outcomes of medication therapy management interventions in randomized control trials and non-randomized control trials: A protocol for systematic review and meta-analysis. Medicine 101 (43), e31491. 10.1097/MD.0000000000031491 36316852 PMC9622691

[B15] DiMatteo M. R. (2004). Variations in patients' adherence to medical recommendations: A quantitative review of 50 years of research. Med. Care 42 (3), 200–209. 10.1097/01.mlr.0000114908.90348.f9 15076819

[B16] Dürr P. Schlichtig K. Kelz C. Deutsch B. Maas R. Eckart M. J. (2021). The randomized AMBORA trial: Impact of pharmacological/pharmaceutical care on medication safety and patient-reported outcomes during treatment with new oral anticancer agents. J. Clin. Oncol. 39 (18), 1983–1994. 10.1200/JCO.20.03088 33822650

[B17] Finley P. R. Crismon M. L. Rush A. J. (2003). Evaluating the impact of pharmacists in mental health: A systematic review. Pharmacotherapy 23 (12 I), 1634–1644. 10.1592/phco.23.15.1634.31952 14695043

[B18] Gunter M. J. (2023). The role of the ECHO model in outcomes research and clinical practice improvement. Am. J. Manag. Care 5, S217–S224. Available at: https://pubmed.ncbi.nlm.nih.gov/10387542/ (Accessed February 28, 2023). 10387542

[B19] Guyatt G. H. Oxman A. D. Vist G. E. Kunz R. Falck-Ytter Y. Alonso-Coello P. (2008). Grade: An emerging consensus on rating quality of evidence and strength of recommendations. BMJ 336, 924–926. 10.1136/bmj.39489.470347.AD 18436948 PMC2335261

[B20] Hattingh H. L. Scahill S. Fowler J. L. Wheeler A. J. (2016). Exploring an increased role for Australian community pharmacy in mental health professional service delivery: Evaluation of the literature<sup/>. J. Ment. Health 25 (6), 550–559. 10.3109/09638237.2015.1101418 26607639

[B21] Hayes B. D. Zaharna L. Winters M. E. Feemster A. A. Browne B. J. Hirshon J. M. (2012). To-Go medications for decreasing ED return visits. Am. J. Emerg. Med. 30 (9), 2011–2014. 10.1016/j.ajem.2012.01.027 22424997

[B22] Hepler C. D. Strand L. M. (1990). Opportunities and responsibilities in pharmaceutical care. Am. J. Hosp. Pharm. 47 (3), 533–543. 10.1093/ajhp/47.3.533 2316538

[B40] Hastert R. Denni J. , H.R.1-Medicare prescription drug, improvement, and modernization Act of 2003 [Online]. Available at: https://www.congress.gov/bill/108th-congress/house-bill/1 [Accessed 8/30 2022].

[B23] Hill-Taylor B. Sketris I. Hayden J. Byrne S. O'Sullivan D. Christie R. (2013). Application of the STOPP/START criteria: A systematic review of the prevalence of potentially inappropriate prescribing in older adults, and evidence of clinical, humanistic and economic impact. J. Clin. Pharm. Ther. 38 (5), 360–372. 10.1111/jcpt.12059 23550814

[B24] Hohmeier K. C. Wheeler J. S. Turner K. Vick J. S. Marchetti M. L. Crain J. (2019). Targeting adaptability to improve Medication Therapy Management (MTM) implementation in community pharmacy. Implement Sci. 14, 99, 10.1186/s13012-019-0946-7 31775801 PMC6882346

[B25] Isetts B. J. Schondelmeyer S. W. Artz M. B. Lenarz L. A. Heaton A. H. Wadd W. B. (2008). Clinical and economic outcomes of medication therapy management services: The Minnesota experience. 2003 48 (2), 203–214. 10.1331/JAPhA.2008.07108 18359733

[B26] Jeremy H. Iain C. Paul G. Trish G. Carl H. Alessandro L. (2011). Explanation of the 2011 oxford centre for evidence-based medicine (OCEBM) levels of evidence (background document). Oxford centre for evidence-based medicine. Available at: https://www.cebm.ox.ac.uk/resources/levels-of-evidence/ocebm-levels-of-evidence (Assessed 2 28, 2023).

[B27] Johnson M. Jastrzab R. Tate J. Johnson K. Hall-Lipsy E. Martin R. (2018). Evaluation of an academic-community partnership to implement MTM services in rural communities to improve pharmaceutical care for patients with diabetes and/or hypertension. J. Manag. Care Spec. Pharm. 24 (2), 132–141. 10.18553/jmcp.2018.24.2.132 29384026 PMC10397983

[B28] Kang J. E. Yu J. M. Choi J. H. Chung I. M. Pyun W. B. Kim S. A. (2018). Development and clinical application of an evidence-based pharmaceutical care service algorithm in acute coronary syndrome. J. Clin. Pharm. Ther. 43 (3), 366–376. 10.1111/jcpt.12665 29468708

[B29] Khalil H. Bell B. Chambers H. Sheikh A. Avery A. J. (2017). Professional, structural and organisational interventions in primary care for reducing medication errors. Cochrane Database Syst. Rev. 2017. 10.1002/14651858.cd003942.pub3 PMC6485628 28977687

[B30] Kozma C. M. Reeder C. E. Schulz R. M. (1993). Economic, clinical, and humanistic outcomes: A planning model for pharmacoeconomic research. Clin. Ther. 15 (6), 1121–1132. 8111809

[B31] Lea M. Mowé M. Molden E. Kvernrød K. Skovlund E. Mathiesen L. (2020). Effect of medicines management versus standard care on readmissions in multimorbid patients: A randomised controlled trial. BMJ Open 10, e041558. 10.1136/bmjopen-2020-041558 PMC7778779 33376173

[B32] Maria J. L. Anand T. N. Dona B. Prinu J. Prabhakaran D. Jeemon P. (2021). Task-sharing interventions for improving control of diabetes in low-income and middle-income countries: A systematic review and meta-analysis. Lancet Glob. Health 9, e170–e180. 10.1016/S2214-109X(20)30449-6 33242455 PMC8279953

[B33] McFarland M. S. Buck M. L. Crannage E. Armistead L. T. Ourth H. Finks S. W. (2021). Assessing the impact of comprehensive medication management on achievement of the quadruple aim. Am. J. Med. 134 (4), 456–461. 10.1016/j.amjmed.2020.12.008 33472055

[B34] Mekonnen A. B. McLachlan A. J. Brien J. E. (2016). Effectiveness of pharmacist-led medication reconciliation programmes on clinical outcomes at hospital transitions: A systematic review and meta-analysis. BMJ open 6, e010003. 10.1136/bmjopen-2015-010003 PMC4769405 26908524

[B35] Moczygemba L. R. Alshehri A. M. Harlow L. D. 3rd Lawson K. A. Antoon D. A. McDaniel S. M. (2019). Comprehensive health management pharmacist-delivered model: Impact on healthcare utilization and costs. Am. J. Manag. Care 25 (11), 554–560. 31747234

[B36] Morgan S. R. Acquisto N. M. Coralic Z. Basalyga V. Campbell M. Kelly J. J. (2018). Clinical pharmacy services in the emergency department. Am. J. Emerg. Med. 36 (10), 1727–1732. 10.1016/j.ajem.2018.01.056 29475633

[B37] Ni W. Colayco D. Hashimoto J. Komoto K. Gowda C. Wearda B. (2018). Budget impact analysis of a pharmacist-provided transition of care program. J. Manag. Care & Specialty Pharm. 24 (2), 90–96. 10.18553/jmcp.2018.24.2.9 PMC10398153 29384028

[B38] Osterberg L. Blaschke T. (2005). Adherence to medication. N. Engl. J. Med. 353 (5), 487–497. 10.1056/NEJMra050100 16079372

[B39] Page M. J. McKenzie J. E. Bossuyt P. M. Boutron I. Hoffmann T. C. Mulrow C. D. (2021). The PRISMA 2020 statement: An updated guideline for reporting systematic reviews. BMJ 372, n71. 10.1136/bmj.n71 33782057 PMC8005924

[B41] Rabin R. de Charro F. (2001). EQ-5D: A measure of health status from the EuroQol group. Ann. Med. 33 (5), 337–343. 10.3109/07853890109002087 11491192

[B42] Rankin A. Cadogan C. A. Patterson S. M. Kerse N. Cardwell C. R. Bradley M. C. (2018). Interventions to improve the appropriate use of polypharmacy for older people. Cochrane Database Syst. Rev. 9, Cd008165. 10.1002/14651858.CD008165.pub4 30175841 PMC6513645

[B43] Reeder C. E. (1995). Overview of pharmacoeconomics and pharmaceutical outcomes evaluations. Am. J. Health Syst. Pharm. 52, S5–S8. 10.1093/ajhp/52.19_Suppl_4.S5 8846244

[B44] Reeder C. E. Gourley G. A. Wurtzbacher J. D. Reed P. (2000). The impact of angiotensin-converting enzyme inhibitors on managed care: Economic, clinical, and humanistic outcomes. Am. J. Manag. Care 6, S112–S128. 10977441

[B45] Rubio-Valera M. Chen T. F. O’Reilly C. L. (2014). New roles for pharmacists in community mental health care: A narrative review. Int. J. Environ. Res. Public Health 11 (10), 10967–10990. 10.3390/ijerph111010967 25337943 PMC4211017

[B46] Shrestha S. Shrestha R. Ahmed A. Sapkota B. Khatiwada A. P. Christopher C. M. (2022). Impact of pharmacist services on economic, clinical, and humanistic outcome (ECHO) of south asian patients: A systematic review. J. Pharm. Policy Pract. 15, 37. 10.1186/s40545-022-00431-1 35538500 PMC9088065

[B47] Silva S. N. Lima M. G. Ruas C. M. (2018). Pharmaceutical interventions in mental health: A review of the literature to support evidence-informed policymaking. Res. Soc. Adm. Pharm. 14 (10), 891–900. 10.1016/j.sapharm.2017.11.014 29195731

[B48] Singhal P. K. Raisch D. W. Gupchup G. V. (1999). The impact of pharmaceutical services in community and ambulatory care settings: Evidence and recommendations for future research. Ann. Pharmacother. 33 (12), 1336–1355. 10.1345/aph.18440 10630834

[B49] Sterne J. A. C. Savović J. Page M. J. Elbers R. G. Blencowe N. S. Boutron I. (2019). RoB 2: A revised tool for assessing risk of bias in randomised trials. Bmj 366, l4898. 10.1136/bmj.l4898 31462531

[B50] Sterne J. A. Hernán M. A. Reeves B. C. Savović J. Berkman N. D. Viswanathan M. (2016). ROBINS-I: A tool for assessing risk of bias in non-randomised studies of interventions. Bmj 355, i4919. 10.1136/bmj.i4919 27733354 PMC5062054

[B51] Thompson H. Swander L. Cohen R. Lukazewski A. Bartholow T. Pesik M. (2020). Hypertension-focused medication therapy management: A collaborative Pilot program uniting pharmacists, public health, and health insurers in Wisconsin. Prev. Chronic Dis. 17 (E105), E105. 10.5888/pcd17.200058 32915128 PMC7553213

[B52] Viswanathan M. Kahwati L. C. Golin C. E. Blalock S. J. Coker-Schwimmer E. Posey R. (2015). Medication therapy management interventions in outpatient settings: A systematic review and meta-analysis. JAMA Intern Med. 175 (1), 76–87. 10.1001/jamainternmed.2014.5841 25401788

[B53] Wang X. Wang S. Yu X. Ma Z. Wang H. Yang J. (2021). Impact of pharmacist-led medication therapy management in ambulatory elderly patients with chronic diseases. Br. J. Clin. Pharmacol. 87 (7), 2937–2944. 10.1111/bcp.14709 33474758

[B54] Zhao X. Shah D. Gandhi K. Wei W. Dwibedi N. Webster L. (2019). Clinical, humanistic, and economic burden of osteoarthritis among noninstitutionalized adults in the United States. Osteoarthr. Cartil. 27 (11), 1618–1626. 10.1016/j.joca.2019.07.002 31299387

