# Supplementary material for: Clinical, economic and humanistic outcomes of medication therapy management services: A systematic review and meta-analysis
Source: Front Pharmacol. 2023 Apr 5;14:1143444. doi: 10.3389/fphar.2023.1143444 (PMC10113465; doi:10.3389/fphar.2023.1143444)
Supplement: Supplementary file 1 [file Table1.DOCX]

Supplementary Material

Clinical, economic and humanistic outcomes of medication therapy management services: A systematic review and meta-analysis

**Zhi-Jie Deng, Lin Gui, Jing Chen, Shun-Shun Peng, Yu-Feng Ding^*^, An-Hua Wei^*^**

*** Correspondence:**An-Hua Wei: ahwei0716@163.com

Yu-Feng Ding: yfding463@163.com

# Supplementary Figures and Tables

## Supplementary Tables

| **No.** | **Year** | **Author** | **Type** | **Location** | **Sample Size, n, I/C** | **Age** | **Description** | **Disease** | **Disease Classification** | **Intervention** | **Comparison** | **Duration of Follow-up** |
| --- | --- | --- | --- | --- | --- | --- | --- | --- | --- | --- | --- | --- |
| 1(Dumlu et al., 2021) | 2022 | Dumlu | NRCT | TR | 100/97 | Median, IQR IG: 78.5 (72-85.8) CG: 79 (70-86) | Patients aged 65 years and older, admitted to the hospital for any reason and ordered to receive for at least one PPI dose within 48 h of admission |  | Undefined | Clinical pharmacist-led stewardship program for the appropriate use of acid suppression therapy (including medication reconciliation and medication review) during the hospital stay | Usual care | 180d |
| 2(Schnipper et al., 2021) | 2021 | Schnipper | RCT | US | 978/679 | 18+ | Admitted to designated hospital within a pioneer accountable care organization |  | Undefined | Multicomponent intervention including inpatient pharmacist-led medication reconciliation, coordination of care between an inpatient “discharge advocate” and a primary care “responsible outpatient clinician,” postdischarge phone calls, and postdischarge primary care visit. | Usual care | 30±5d |
| 3(Charra et al., 2021) | 2021 | Charra | NRCT | FR | 26/35 | Mean, Range IG: 51.8 (29-66) CG: 50.3 (23-70) | All patients who received allogeneic hematopoietic stem cell transplant (HSCT) | Allogeneic Hematopoietic Stem Cell Transplant | Non-Chronic Disease | The first consultation was performed while the patient was in the care unit, the day before discharge. The next consultations were performed during day-care follow-up, at weeks 2 and 4 after discharge, and then once a month until day 100 post-transplantation. | Usual care | 100d |
| 4(Bouchand et al., 2021) | 2021 | Bouchand | RCT | FR | 60/60 | Median, IQR IG: 68.5 (51.75-77) CG: 69 (56.25-74.75) | Hospitalisation in the infectious disease department with at least one chronic disease, current medical prescription at admission and discharged from hospital to home or nursing home. | Chronic Disease | Chronic Disease | A discharge care bundle including medication reconciliation, counselling session and documentation transfer to primary care physician (PCP) | Standard of care | 30d |
| 5(Freeman et al., 2021) | 2021 | Freeman | RCT | AU | 129/177 | Mean±SD IG: 70.8±12.4 CG: 69.3±13.7 | Adults discharged from one of seven study hospitals during the seven days preceding recruitment (22 May 2017 ‒ 14 March 2018) and prescribed five or more long term medicines, or having a primary discharge diagnosis of congestive heart failure or exacerbation of chronic obstructive pulmonary disease. | Chronic Disease | Chronic Disease | Comprehensive face-to-face medicine management consultation with an integrated practice pharmacist within seven days of discharge, followed by a consultation with their general practitioner and further pharmacist consultations as needed. | Usual care | 360d |
| 6(Nymberg et al., 2021) | 2021 | Nymberg | RCT | SE | 182/187 | Mean±SD IG: 87±5.8 CG: 87.7±5.5 | Users of the multi-dose drug dispensing system aged 75 years or older, living in nursing homes or community dwelling with municipally provided home care. |  | Undefined | Pharmacists performed a systematic MR based on a symptom assessment made by a nurse with good knowledge of the patient. The MR consisted of a structured assessment in three steps.3 First, a nurse conducted a specific symptom evaluation and health status check including blood pressure, pulse, weight, tendency to fall and confusion and other symptoms using a validated symptom assessment form. | Usual care | 360d |
| 7(Kempen et al., 2021) | 2021 | Kempen | RCT | SE | 1145 (922+823)/892 | Median, IQR CMR: 81 (74-87) CMR-FUP: 81 (74-87) UC: 80 (74-87) | Age 65 years or older and admission to one of the participating wards for at least one 24-hour period on regular weekdays. |  | Undefined | Comprehensive medication review, consisted of a medication reconciliation by a clinical pharmacist with the patient, next-of-kin, or caregiver within 24 hours after hospital admission to ensure a correct list of medications. | Usual care | 360d |
| 8(Liu et al., 2021) | 2021 | Liu | RCT | CN | 96/97 | Mean±SD IG: 75.13±8.03 CG: 73.25±7.45 | Patient a) aged over 40 years old, b) treated by the Department of Respiratory and Critical Care Medicine for COPD, c) met the COPD diagnostic criteria given in the Global Initiative for Chronic Obstructive Lung Disease (GOLD) guidelines and free of hearing defect, disorders of speech and language or cognitive impairment, d) voluntary to participate in this study with informed consent, and e) able to maintain good communication with researchers to complete this study as required. | COPD | Chronic Disease | The experimental group was provided with MTM services combined with the conventional treatment. | The control group was treated with conventional medication, including antibacterials, bronchodilators, inhaled hormones, and expectorants. | 180d |
| 9(Herawati et al., 2021) | 2021 | Herawati | NRCT | ID | 30/33 | Mean±SD IG: 72.5±9.2 CG: 67.8±4.8 | Older adults (>60 years old) who were hospitalized with a non-emergency degenerative disease or non-acute infection sepsis diagnosis, received polypharmacy (with 5-7 drugs), and used national or district health financing coverage. |  | Undefined | In the intervention group, the pharmacist and the physicians applied the STOPP/START criteria in a collaborative fashion. | The control group received standard care. | 90d |
| 10(Ceschi et al., 2021) | 2021 | Ceschi | RCT | CH | 866/836 | Median, IQR IG: 86 (79-89) CG: 86 (79.8-90) | Aged 85 years or older, with more than 10 medications at hospital admission (ie, home medications recorded by the attending physician in an unstructured and nonsystematic manner), or meeting both conditions. |  | Undefined | Medication Reconciliation | Stamdard care | 500d |
| 11(Lu et al., 2021) | 2021 | Lu | RCT | CN | 60/59 | Mean±SD IG: 56.75±12.05 CG: 53.17±11.44 | (1) T2DM confirmed for more than 1 year and HbA1c 7–10.0%; (2) age between 18 and 75 years; and (3) adjusting the dosage or switching to a different medication. | T2DM | Chronic Disease | In-hospital medication evaluation, drug reorganization, medical monitoring, and advice via telemedicine | Standard care | 180d |
| 12(Östbring et al., 2021) | 2021 | Östbring | RCT | SE | 159/157 | Mean±SD IG: 68.3±8.9 CG: 68.6±8.6 | Patients with angiographically verified CHD who were scheduled for follow-up at the out-patient clinic were eligible to participate if they spoke Swedish. | CHD | Chronic Disease | A follow-up program and MTM services run by two clinical pharmacists in addition to standard care. | Standard care | 450d |
| 13(Turakhia et al., 2021) | 2021 | Turakhia | RCT | US | 72/67 | Mean±SD IG: 64.72±9.05 CG: 65.36±10.28 | Participants age ≥18 years with documented atrial fibrillation who were taking or initiating newly-prescribed rivaroxaban (≤90 days of consumption or a prescription dated in the last 90 days) | Atrial Fibrillation | Chronic Disease | The study intervention was a centralized, blended process with several personalized components: (1) medication and refill tracking smartphone application (Care4Today version 2.0 or higher, Janssen Health Innovation, La Jolla, CA); (2) semiautomated text messaging platform; (3) coach-to-patient phone call system. | Usual care by their primary AF clinician, who was the DOAC-prescribing clinician. | 180d |
| 14(Song et al., 2021) | 2021 | Song | RCT | KR | 50/50 | Mean±SD IG: 51±16.6 CG: 54±17.4 | Patients were eligible if they were (1) new admissions in the nephrology ward, (2) over 18 years of age, (3) diagnosed with CKD by their nephrologist previously, (4) confirmed to have either an eGFR of less than 60 mL/min/1.73 m2 or kidney damage, as shown by laboratory results in SNUH, within 3 months prior to the hospitalization, and (5) found to have one or more complications of CKD, such as hypertension, diabetes, dyslipidemia, acid-base and electrolyte disorders, anemia, mineral bone disorder, and hyperuricemia, or use one or more drugs for the treatment of these complications. | CKD | Chronic Disease | The collaborative multidisciplinary drug therapy evaluation and management (DrugTEAM) service based on the internal guidelines for inpatients | Usual care from pharmacists and physicians, without implementing DrugTEAM service model. | 90d |
| 15(Liou et al., 2021) | 2021 | Liou | RCT | TW | 50/50 | Mean±SD IG: 86.7±5.6 CG: 85.7±3.6 | Age 65 years or older, prescriptions for at least 5 oral medicines daily, and ≥2 chronic diseases, for the period May 2013 to October 2014. |  | Undefined | A unification intervention approach, which included completing the medication administration record, assessing medication appropriateness of prescriptions, surveying the utilization of healthcare resources, and identifying drug-related problems | Usual care, without a medication review by a visiting pharmacist | 540d |
| 16(Dürr et al., 2021) | 2021 | Dürr | RCT | DE | 98/104 | Mean, Range IG: 65.5 (32-91) CG: 66.5 (36-88) | Eligible patients were ≥ 18 years and were scheduled by the treating physician for start of treatment with a new oral antitumor drug. | Cancer | Non-Chronic Disease | Intensified clinical pharmacological/pharmaceutical care, which included medication management and structured patient counseling and standard care | Standard care | 84d (12wk) |
| 17(Lea et al., 2020) | 2020 | Lea | RCT | NO | 193/193 | Median, Range IG: 78 (25.7-95.6) CG: 80.7 (23.1-96.4) | Using minimum four regular drugs from minimum two therapeutic classes. |  | Undefined | Pharmacist-led medicines management | Standard care | 360d |
| 18(Yin et al., 2020) | 2020 | Yin | RCT | CN | 31/30 | Mean±SD IG: 47.5±18.3 CG: 46.3±19 | Older than 18 years, with NS identified by 24-h urinary protein≥3.5 g and serum albumin≤30 g/L. | Nephrotic Syndrome | Chronic Disease | A pharmacist-delivered multifaceted intervention in addition to usual care. | Usual care | 180d |
| 19(Romskaug et al., 2020) | 2020 | Romskaug | RCT | NO | 87/87 | Mean±SD IG: 82.2±7.6 CG: 84.4±6.9 | Patients were home-dwelling individuals, were 70 years or older, used at least 7 systemic medications taken regularly, and had their medications administered by the home nursing service. |  | Undefined | The intervention consisted of 3 main parts: (1) clinical geriatric assessment of the patients combined with a thorough review of their medications; (2) a meeting between the geriatrician and the FP; and (3) clinical follow-up. | Usual care | 167d (24wk) |
| 20(Auvinen et al., 2020) | 2020 | Auvinen | RCT | FI | 258/254 | Mean±SD IG: 84.41±6.68 CG: 84.67±6.17 | Age ≥ 65 years and registration to public home care services, and at least one of the following: ≥ 6 medicines in use, dizziness, orthostatic hypotension or a recent fall. | Chronic Disease | Chronic Disease | The structured medication assessment included a review of medication, an assessment of the clinical information, and a meeting of an interprofessional team consisting of a pharmacist, physician and registered nurse working regularly in home care; they conducted the medication assessment within two weeks after the baseline measurements. | Usual care | 180d |
| 21(Desborough et al., 2020) | 2020 | Desborough | RCT | UK | 381/445 | Mean±SD IG: 88.4±6.5 CG: 86±8.5 | Providing care for residents with an average age >65 years |  | Undefined | Intervention homes received a MPMR at the care home, from a team consisting of a clinical pharmacist (with a postgraduate diploma in clinical or general pharmacy practice), GP and care home member of staff responsible for medication, with preparation undertaken by a pharmacy technician | Usual care | 360d |
| 22(AbuNaba'a and Basheti, 2020) | 2020 | AbuNaba'a | RCT | JO | 36/37 | Mean±SD IG: 43.28±16.3 CG: 40.38±16.2 | Jordanian females or residents in Jordan for the last 12 months and planning to stay in Jordan for 6 months (the study follow-up period), above the age of 18 years, diagnosed with depression and/or anxiety for at least 4 weeks duration, and taking medications for depression and/or anxiety. | Depression/Anxiety | Chronic Disease | Medication Management Review service (MMR): pharmacist-delivered counseling and a letter with recommended changes in the patient's treatment plan were sent to the patient's psychiatrist to be applied. | Usual care | 90d |
| 23(Manley et al., 2020) | 2020 | Manley | Cohort | US | 866/586 | Mean±SD IG: 61.5±14.8 CG: 63±15 | Discharges to home were identified in Medicare Part A claims data. To account for patients with multiple discharges, “frequent” hospitalization was defined as 3 or more discharges in the previous 6 months before the first discharge within the study period. |  | Undefined | MTM Services | Usual care |  |
| 24(Heaton et al., 2019) | 2019 | Heaton | RCT | US | 213/187 | Mean±SD IG: 60.3±12.9 CG: 63.2±12.3 | Patients discharged from a participating hospital with acute myocardial infarction, pneumonia, congestive heart failure, chronic obstructive pulmonary disease, or diabetes. | Chronic Disease | Chronic Disease | MTM Services | Usual care | 30d |
| 25(Bloodworth et al., 2019) | 2019 | Bloodworth | RCT | US | 96/160 | Mean±SD IG: 52.63±13.6 CG: 57.54±15.1 | Admitted to UMMC for inpatient treatment of one of the following: AMI, PNA, HF, or COPD | Chronic Disease | Chronic Disease | Pharmacist transitions coordinator; telephonic and face-to-face interventions at discharge | Usual care | 180d |
| 26(Brühwiler et al., 2019) | 2019 | Brühwiler | RCT | SZ | 76/76 | Median, IQR IG: 72 (61-79) CG: 71 (57-79) | Patients of the internal medicine department, ≥18 years, without isolation due to infection, with standard or semi-private insurance, without cognitive impairment (e.g. acute delirium or severe dementia) that hinders patients from giving consent. | Chronic Disease | Chronic Disease | Medication reconciliation at discharge | Usual care | 30d |
| 27(Graabaek et al., 2019) | 2019 | Graabaek | RCT | DK | 400 (200+200)/200 | Median, IQR ED: 74 (69-80) STAY: 74 (69-80) CG: 75 (70-82) | 65 years of age or above, acutely admitted, medical patients (not surgical), able to give informed consent, able to speak and understand Danish, and holding a Danish personal registration number. |  | Undefined | ED: Basic Intervention STAY: Extended Intervention Both the ED group and the STAY group received a pharmacist-led medication review (including patient interview and medication reconciliation) on admission. Furthermore, patients in the STAY group transferred to a specialized ward received a medication review during inpatient stay together with patient counselling and a medication report at discharge. | usual care including medication history, medication reconciliation, and medication review by a physician without any structured instrument | 180d |
| 28(Tamblyn et al., 2019) | 2019 | Tamblyn | RCT | CA | 1690/1877 | Mean±SD IG: 70.6±13.6 CG: 69±15.9 | All patients who were covered by provincial drug insurance, which includes seniors, welfare recipients, and those without access to employer-based private drug insurance, and who were discharged to the community or a long-term care facility from the 2 internal medicine units, the cardiac surgery unit, or the thoracic surgery unit. |  | Undefined | The RightRx intervention electronically retrieved community drugs from the provincial insurer and aligned them with in-hospital drugs to facilitate reconciliation and communication at care transitions. | Usual care | 90d |
| 29(Verdoorn et al., 2019) | 2019 | Verdoorn | RCT | NL | 315/314 | Median, IQR IG: 80 (76-83) CG: 78 (74-82) | Aged ≥70 years and using ≥7 long-term medications. |  | Undefined | CMR review focused on personal goals, including 1) a patient interview performed by the community pharmacist; 2) summary of DRPs and recommendations were proposed to attain goals and to solve DRPs; 3) a face-to-face meeting with the patient’s GP to discuss all health-related goals and other identified DRPs; 4) the pharmaceutical care plan was then discussed with the patient to reach agreement about implementation; 5) Two follow-up appointments were scheduled. | Usual care | 360d |
| 30(Sloeserwij et al., 2019) | 2019 | Sloeserwij | NRCT | NL | 3879/7732 (3941+3791) | Mean±SD IG: 75±8 CG: 75±8 | Availability of a consultation room for the NDP; access to the GPs' electronic medical records; a minimum of 5000 registered patients; at least 1 practice nurse working on chronic disease management programmes. |  | Undefined | Non‐dispensing pharmacists (NDP)-led care, including clinical medication reviews for patients with polypharmacy, medication reconciliations for patients discharged from the hospital and individual patient consultations for patients with specific drug therapy problems. | Usual care and usual care plus | 360d |
| 31(Pevnick et al., 2018) | 2018 | Pevnick | RCT | US | 205 (103+102)/101 | Mean±SD UC+PI: 72±16 UC+PSPT: 71±16 UC: 71±18 | ≥10 active chronic prescription medications in the electronic health record (EHR), history of acute myocardial infarction or congestive heart failure in the EHR problem list, admission from a skilled nursing facility (SNF), history of transplant, or active anticoagulant, insulin or narrow therapeutic index medications | Chronic Disease | Chronic Disease | Admission medication history (AMH) intervention. Obtaining the initial AMH usually began with reviewing the medication regimen present in the EHR if one was available from a prior encounter. Next, patients, families, and caregivers present in the ED were interviewed. Pill bottles, medication lists, and SNF medication administration records were also reviewed. | Usual AMH care | 30d |
| 32(Kang et al., 2018) | 2018 | Kang | NRCT | KR | 41/38 | Mean±SD IG: 56.8±12.3 CG: 59.2±11.5 | Patients aged 18 years and older who were admitted for acute coronary syndrome (ACS) regardless of the time of diagnosis. | Acute Coronary Syndrome | Non-Chronic Disease | Multidisciplinary team care (MTC) service. The MTC group received medication reconciliation (MedRec) and medication optimization (MedOpt) services during hospitalization, while receiving transition of care (TOC) upon a unit transfer or discharge. After discharge, the MTC group received MedRec and MedOpt in an ambulatory care setting. | Usual care | 180d |
| 33(Jones et al., 2018) | 2018 | Jones | RCT | US | 34/34 | Mean±SD IG: 50.9±17.1 CG: 54.1±13.5 | Patients identified as high risk for readmission in a transitions-of-care (TOC) pilot |  | Undefined | Pharmacist-led TOC pilot program Medication reconciliation, medication counseling, case management or social work evaluation, a postdischarge telephone call, and an expedited primary care follow-up appointment. | Usual care | 90d |
| 34(Al-Hashar et al., 2018) | 2018 | Al-Hashar | RCT | Oman | 286/301 | Mean±SD IG: 56±17 CG: 57±17 | Admitted to medical wards, on at least one medication prior to admission, admitted for at least 24 h, had not been included in this study during a previous admission, and they or their caregiver spoke Arabic or English and could be interviewed for medication history. |  | Undefined | Medication reconciliation | Standard Care | 30d |
| 35(Chiu et al., 2018) | 2018 | Chiu | NRCT | HK | 108/104 | Mean±SD IG: 83.3±5.7 CG: 83.3±5.6 | People aged 65 years or above who are transferred from an acute hospital after initial stabilisation of medical and/or geriatric problems. |  | Undefined | Pharmaceutical care: Medication Reconciliation; Medication review; Pharmacist counselling on admission | Routine clinical services | 90d |
| 36(Lisby et al., 2018) | 2018 | Lisby | RCT | DK | 53/55 | Mean 95%CI IG: 80.4 (78.2-82.6) CG: 80.5 (78.2-82.8) | 65 years or older were eligible for inclusion if they were nonelective, treated with at least 4 drugs at the time of admission, and had an expected in-hospital length of stay (LOS) of a minimum of 24 hours. |  | Undefined | The clinical pharmacist reviewed the participants' medication after completion of the usual medication routine. | Usual medication routine | 90d |
| 37(Lin et al., 2018) | 2018 | Lin | RCT | CN | 87/91 | Mean±SD IG: 77.9±6.1 CG: 78.4±6 | Elderly patients age 65 and older who had three or more chronic diseases (identified by diagnosed IChronic Disease9 codes), more than six prescription items, and had made more than four outpatients visits or visited two or more different specialties in CMUH during an assessment period from November 2007 to October 2008 | Chronic Disease | Chronic Disease | A collaborative pharmacist-physician MTM program | Usual care with follow-up assessment | 360d |
| 38(Tuttle et al., 2018) | 2018 | Tuttle | RCT | US | 72/69 | Mean±SD IG: 70±12 CG: 69±10 | Adult patients with CKD stages 3–5 ND hospitalized for acute illness | CKD | Chronic Disease | The intervention included these fundamental elements: comprehensive medication review, medication action plan, and a personal medication list. | Usual care | 90d |
| 39(Persell et al., 2018) | 2018 | Persell | RCT | US | 540 (262+278)/254 | Mean±SD IG: 52.57±9.64 IG: 53±9.5 | (1) 18 years or older; (2) self-report of 3 or more medications prescribed (for any purpose); (3) systolic blood pressure at enrollment of at least 130 mm Hg or diastolic blood pressure of at least 80 mm Hg with diabetes or systolic blood pressure of at least 135 mmHg or diastolic blood pressure of at least 85 mm Hg without diabetes (the criteria for participants without diabetes was lowered by 5 mmHg during the study); (4) a Mini-Cog examination score of at least 3 (scores range from 0-5, with lower scores indicating a greater chance of dementia); (5) self-report that no one else was responsible for administering medication; (6) no intention to change the source of care during the next year; and (7) ability to communicate in English. | Hypertension | Chronic Disease | EHR Tools, including (1) review medicines, (2) strike out medicines not taken, (3) identify whether medications were taken as described, (4) identify concerns about medications, and (5) add medicines or supplements not included on the list. EHR Tools Plus Nurse-Led Medication Therapy Management, included assessment of medication comprehension, review of the pattern of medication use, reconciliation with the EHR, assistance with regimen dosing consolidation when feasible, and development of a medication table for complex regimens. | Usual care | 360d |
| 40(Wu et al., 2018) | 2018 | Wu | RCT | US | 117/133 | Mean±SD IG: 65.8±8.7 CG: 65±9.8 | All patients >18 years old with documented Type-2 DM in the medical record, last recorded hemoglobin A1c of >7.0% and at least one of the following: being a smoker (any cigarette smoking < 30 days), having an LDL >100 mg/dl in the last blood draw or a blood pressure >130/80 mm Hg documented on at least two occasions within the last 6 months, and able to participate in group discussions for diabetes and cardiovascular risk factors. | T2DM | Chronic Disease | Group Medical Visits: Coordinated and led by clinical pharmacists, consisted of education on diabetes self-care during the first hour followed by behavioral counseling and comprehensive medication management for hyper- (and hypo-) glycemia, hypertension, and dyslipidemia during the second hour. The educational component included interactive lectures that were based on the American Diabetes Association (ADA) Standards of Diabetes Self-Management. | Standard care | 390d (13m) |
| 41(Quintana-Bárcena et al., 2018) | 2018 | Bárcena | RCT | CA | 304/138 | Mean±SD IG: 71.9±12 CG: 71.2±12.5 | (a) aged ≥ 18 years; (b) an eGFR of 30-59 mL/min/1.73m2 (stage 3 CKD) or an eGFR of 15-29 mL/min/1.73m2 (stage 4 CKD), as determined by the most recent laboratory result available in the CKD clinic; (c) speaking English or French; (d) followed by an eligible community pharmacy; and (e) agreeing to be followed by the same community pharmacy for the duration of the study. | CKD | Chronic Disease | The systematic approach was based on the analysis of all relevant information available to ProFiL pharmacists, including the clinical summary. The clinical summary was completed for each patient by staff at the CKD clinic and sent to the community pharmacists to facilitate the detection of DRPs. It included a list of patients’ health problems, their eGFRs according to the Chronic Kidney Disease Epidemiology Collaboration equation,15 and a list of their medications as recorded in the CKD clinic chart. | Usual care | 360d |
| 42(Gustafsson et al., 2017) | 2017 | Gustafsson | RCT | SE | 212/217 | Mean±SD IG: 83.1±6.6 CG: 83.1±6.6 | Aged 65 years or older and had dementia or cognitive impairment. | Dementia/Cognitive Impairment | Chronic Disease | Medication reconciliation, Medication review | Usual care | 180d |
| 43(Campins et al., 2017) | 2017 | Campins | RCT | ES | 252/251 | Mean±SD IG: 79.16±5.5 CG: 78.78±5.46 | Community-dwelling elderly people (non-institutionalized) aged 70 years and older, receiving 8 or more drugs and resident in the municipalities of Mataró and Argentona (Barcelona, Spain). |  | Undefined | Pharmacist review of all medication according to the Good Palliative–Geriatric Practice algorithm and the Screening Tool of Older Person’s Prescriptions-Screening Tool to Alert Doctors to the Right Treatment criteria and recommendations to the patient’s physician. | Routine clinical practice. | 360d |
| 44(Karapinar-Çarkıt et al., 2017) | 2017 | Çarkıt | NRCT | NL | 168/151 | Mean±SD IG: 64.5 (16.5) CG: 64 (15.5) | All admitted patients at the Internal Medicine department using at least one prescribed drug for chronic use at hospital admission. | Chronic Disease | Chronic Disease | The COACH program consisted of medication reconciliation, patient counselling at discharge, and communication to healthcare providers in primary care. | Usual care | 90d |
| 45(Van der Linden et al., 2017) | 2017 | Linden | NRCT | BE | 91/81 | Mean±SD IG: 84.5±4.69 CG: 84.5±4.97 | Dutch-speaking patients admitted from home or from a nursing home |  | Undefined | Intervention consisted of trained clinical pharmacists (EB, SD, KW, LD, LVDL) performing medication reconciliation with a subsequent two-stage medication review. | Usual care | 90d |
| 46(Komagamine and Hagane, 2017) | 2017 | Komagamine | Cohort | JP | 32/132 | Mean±SD IG: 83.8±6.2 CG: 85.1±6.7 | Patients aged 65 years or older who were admitted to the hospital for a hip fracture and who were prescribed five or more medications at admission between January 2015 and December 2016. | Fracture | Non-Chronic Disease | The intervention consisted of an assessment by internal medicine physicians of the appropriateness of polypharmacy and the de-prescription of any unnecessary medications during the patients’ hospital stay. | Usual care | 180d |
| 47(Erku et al., 2017) | 2017 | Erku | RCT | ET | 54/53 | Mean±SD IG: 61.3±11.4 CG: 59.8±13.5 | Patients who were at least 18 years of age, with a diagnosis of type 2 diabetes; taking at least one long-term antidiabetic medication for a at least 3 months and having regular visits (every month) to the hospital for follow-up and medication refill | T2DM | Chronic Disease | The usual care followed by MTM services. | Usual care | 180d |
| 48(Nielsen et al., 2017) | 2017 | Nielsen | RCT | DK | 158/340 | Mean±SD IG: 74.1±11.5 CG: 72.7±13.2 | Medical patients aged 18 years or older taking four or more medications a day (including over-the-counter medicines and dietary supplements) |  | Undefined | The clinical pharmacist intervention comprised medication history, medication reconciliation, medication review and entry of proposed prescriptions into the electronic prescribing system. | Usual care | 360d |
| 49(Hayward et al., 2017) | 2017 | Hayward | RCT | AU | 57/59 | Mean±SD IG: 58.1±10 CG: 58.9±10.7 | Ambulatory patients with cirrhosis who had experienced a decompensation event (ascites, hepatic encephalopathy [HE], or variceal bleed) within the preceding 2 years | Decompensated Cirrhosis | Non-Chronic Disease | Patients received additional review by the pharmacist to obtain a complete reconciled list of current medications and identify MRPs. | Patients received education and clinical review by a hepatologist (or gastroenterology trainee) in a dedicated hepatology clinic. | 360d |
| 50(Moga et al., 2017) | 2017 | Moga | RCT | US | 25/25 | Mean±SD IG: 76.3±6.2 CG: 79.1±6.9 | Actively enrolled in the (Alzheimer’s Disease Center) ADC cohort; 65 years of age and older; reporting at least one drug with anticholinergic properties at their annual ADC visit; and willing to participate in our intervention study. |  | Undefined | A targeted patient-centered pharmacist–physician team MTM intervention | Usual care | 56d (8wk) |
| 51(Haag et al., 2016) | 2016 | Haag | RCT | US | 13/12 | Median, IQR IG: 81 (78-85) CG: 86 (79.5-87) | Independent-living elderly adults (aged ≥60 years) who were enrolled in the local care transitions program (CTP). |  | Undefined | Pharmacist-provided MTM program via telephone | Usual care within an existing outpatient CTP | 30d |
| 52(Phatak et al., 2016) | 2016 | Phatak | RCT | US | 137/141 | Mean IG: 55.4 CG: 55.8 | Patients who discharged to home and either discharged on greater than 3 scheduled prescription medications or discharged with at least 1 high-risk medication. |  | Undefined | On admission: face-to-face medication reconciliation; At discharge: a personalized medication plan was created by the pharmacist and discussed with the physician. | Usual care | 30d |
| 53(O'Sullivan et al., 2016) | 2016 | Sullivan | RCT | IE | 361/376 | Median, IQR IG: 77 (71-83) CG: 78 (72-84) | All patients aged ≥65 years admitted under the care of the medical or surgical services through the emergency department |  | Undefined | The patients in the intervention group received the Chronic DiseaseSS-supported SPRM intervention alongside usual medical and pharmaceutical care. | Usual care | 390d |
| 54(Basheti et al., 2016) | 2016 | Basheti | RCT | JO | 48/49 | Mean±SD IG: 63.13±7.99 CG: 58.39±12.8 | (1) Jordanian or a resident in Jordan for the last 12 months and intending to stay in the country for the 3 months study period; (2) 18 years or older; (3) patients with either of the following: at least one chronic condition, taking 5 or more medications, taking more than 12 doses of medication per day, recently discharged from hospital (in the last 4 weeks), had significant changes made to their medication regimen in the last 3 months (preset criteria included: ceasing and/or starting new medication/s for chronic condition/s), had symptoms suggestive of an adverse drug reaction, or showed sub-therapeutic response to medication treatment. | Chronic Disease | Chronic Disease | All patients were visited at home by the pharmacist who delivered only for intervention group counseling regarding self-reported adherence, frequency of monitoring and education regarding pharmacological and non-pharmacological therapy. | Usual care | 90d |
| 55(Elliott et al., 2016) | 2016 | Elliott | RCT | UK | 251/253 | Mean±SD IG: 59.5±15.3 CG: 59.3±15 | Eligible for NMS, community-dwelling, aged 14 years or over, able to consent to the NMS and the study and willing to provide written consent (parental consent for 14-year-olds and 15- year-olds). |  | Undefined | NMS intervention: One consultation 7–14 days after presentation of prescription followed by another 14–21 days thereafter to identify problems with treatment and provide support if needed. | Normal practice | 70d (10wk) |
| 56(Bajorek et al., 2016) | 2016 | Bajorek | RCT | AU | 10/11 | Mean±SD IG: 69.7±13.2 CG: 71.6±16.1 | Aged ≥18 years of age able to provide written informed consent to participate in study able to return for all follow-up visits and were accessible by telephone had a diagnosis of essential hypertension (new diagnosis or established diagnosis) which was not controlled and meeting criteria for therapy as defined by current Australian guidelines. |  | Undefined | A pharmacist-led service, based on the Health Collaboration Model (HCM) | Usual care | 360d |
| 57(Malet-Larrea et al., 2016) | 2016 | Larrea | RCT | ES | 688/715 |  | Aged patients (65 years or older), using polypharmacy (five or more medications for at least 6 months) and with the ability to complete the EuroQol 5D questionnaire. |  | Undefined | The MRF service starts with a comprehensive interview undertaken in a private area of the pharmacy. After performing a comprehensive medication review, the pharmacist identifies negative clinical outcomes related to medicines and DRPs. Subsequently, an action plan is agreed upon by the patient and the physician if required. This MRF service is focused on both patients' outcomes and medication use process and requires a commitment to follow‐up. | Usual care | 180d |
| 58(Bell et al., 2015) | 2015 | Bell | RCT | US | 430/432 | Mean±SD IG: 61±14 CG: 59±14 | Adults hospitalized with a diagnosis of ACS and/or ADHF | Acute Coronary Syndrome (ACS)/Acute Decompensated Heart Failure (ADHF) | Non-Chronic Disease | Pharmacist-assisted medication reconciliation, inpatient pharmacist counseling, low-literacy adherence aids, and individualized telephone follow-up after discharge | Usual care | 30d |
| 59(NCT02482025) | 2015 | NCT | RCT | US | 118/122 | Mean±SD IG: 63.4±11.7 CG: 66.1±11.8 | Veterans age 18 years or older Having a VA primary care provider (PCP) at any VA facility in VISN-1 Planned discharge home (as opposed to another facility) Computer and internet access Anticipated to be discharged with at least 5 medications. |  | Undefined | Secure Messaging for Medication Reconciliation Tool (SMMRT) Includes My HealtheVet registration and enrollment | Usual care | 30d |
| 60(2015) | 2015 | SUREPILL | RCT | NL | 547/547 | 17+ | Consecutive patients admitted for elective surgery with expected hospital stay longer than 48h |  | Undefined | Received care by a ward-based pharmacy team, consisting of pharmacy practitioners (specialized pharmacy technicians) and hospital pharmacists | Standard pharmaceutical care from a pharmacy team in their traditional role of taking responsibility for the appropriate, safe and cost-effective use of medication from a central pharmacy. | 90d |
| 61(Hohl et al., 2015) | 2015 | Hohl | RCT | CA | 6416/4391 | Mean±SD IG: 71±31 CG: 69±33 | Consecutive high-risk patients aged 19 years or older presenting when a medication review pharmacist was on duty |  | Undefined | The intervention, early in-hospital pharmacist-led medication review, consisted of obtaining a best-possible medication history, discussing the goals of therapy with the patient or caregiver, and reviewing the patient’s medications to identify and resolve medication-related problems, including adverse drug events, unintended and harmful events related to medication use, and optimize medication effectiveness and safety. | Usual care | 360d |
| 62(Briggs et al., 2015) | 2015 | Briggs | RCT | AU | 525/496 | Mean±SD IG: 82±6 CG: 81±6 | Patients lived at home and reported taking more than five medications daily |  | Undefined | The pharmacist obtained informed consent to participate in the trial and undertook initial data collection and stratification of the older person by completing the ISAR questionnaire. | Usual care | 360d |
| 63(Basger et al., 2015) | 2015 | Basger | RCT | AU | 114/102 | Mean±SD IG: 82.7±7.3 CG: 80.2±6.7 | Age ≥65 years, English-speaking, taking five or more medications and living within a 15 km radius of the hospital. |  | Undefined | Discharge medication counselling and a medication review by a clinical pharmacist. | Usual care | 90d |
| 64(Westberg et al., 2014) | 2014 | Westberg | NRCT | US | 135/270 | Mean±SD IG: 75.9±7 CG: 76.2±7.6 | Aged 65 years or older and discharged from the hospital after being admitted for heart failure, ischemic heart disease, dysrhythmias, genitourinary conditions, or digestive disorders. | Chronic Disease | Chronic Disease | An initial comprehensive medication management visit following hospital discharge and prior to their hospital follow-up appointments with their primary care providers. | Usual care | 180d |
| 65(Farris et al., 2014) | 2014 | Farris | RCT | US | 623 (311+312)/313 | 18+ | English or Spanish speaker, 18 years or older, admitted with diagnosis of hypertension, hyperlipidemia, heart failure, coronary artery disease, myocardial infarction, stroke, transient ischemic attack, asthma, chronic obstructive pulmonary disease or receiving oral anticoagulation. | Chronic Disease | Chronic Disease | Intervention groups received admission medication reconciliation, pharmacist visits every 2–3 days for patient education during inpatient stay, discharge counseling and discharge medication list. | Usual care | 90d |
| 66(Zillich et al., 2014) | 2014 | Zillich | RCT | US | 415/480 | Mean±SD IG: 73±13 CG: 73±13 | All new patients within each care center who were admitted into Medicare’s defined 60-day home health care episode were eligible, including skilled nursing care and “therapy only” patients (i.e., those receiving physical/occupational therapy services only). |  | Undefined | The MTM intervention consisted of the following: (1) initial phone call by a pharmacy technician to verify active medications; (2) pharmacist-provided medication regimen review by telephone; and (3) follow-up pharmacist phone calls at day seven and as needed for 30 days. | Usual care | 60d |
| 67(Ho et al., 2014) | 2014 | Ho | RCT | US | 122/119 | Mean±SD IG: 63.8±9.25 CG: 64±8.57 | Patients admitted with ACS as the primary reason for hospital admission and used the Veterans Affairs (VA) for their usual care | Acute Coronary Syndrome | Non-Chronic Disease | The INT lasted for 1 year following discharge and comprised (1) pharmacist-led medication reconciliation and tailoring; (2) patient education; (3) collaborative care between pharmacist and a patient’s primary care clinician and/or cardiologist; and (4) 2 types of voice messaging (educational and medication refill reminder calls). | Usual care | 360d |
| 68(Wittayanukorn et al., 2013) | 2013 | Wittayanukorn | Cohort | US | 63/62 | Mean±SD IG: 56.8±9.3 CG: 56.9±9.6 | Patients were required to have at least 1 diagnosis code for CVD conditions, including hypertension, hyperlipidemia, coronary heart disease, heart failure, stroke, and other form of heart disease. |  | Chronic Disease | MTM Services | Usual care | 100d |
| 69(Touchette et al., 2012) | 2012 | Touchette | RCT | US | 429/208 | Mean±SD IG: 74.7±6.7 CG: 74.6±6.8 | Individuals aged 65 years or older with three or more chronic illnesses, six or more prescription medications, and at risk for a DRP. |  | Undefined | At 0 and 3 months, pharmacists conducted comprehensive medication reviews and screened for and resolved DRPs through patient education and recommendations to physicians. | Usual care | 180d |
| 70(Moczygemba et al., 2012) | 2012 | Moczygemba | NRCT | US | 60/60 | Mean±SD IG: 71.2±7.5 CG: 73.9±8 | Medicare Part D beneficiaries who were MTM eligible |  | Undefined | Pharmacist-provided telephone MTM consultation | Usual care | 360d |
| 71(Pai et al., 2009) | 2009 | Pai | RCT | US | 57/47 | Mean±SD IG: 56.3±15 CG: 60.5±14.7 | Patients had to speak English, and be older than 18 years and undergoing a stable hemodialysis regimen for at least 3 months | CKD | Chronic Disease | Medication reviews conducted by a nephrology-trained clinical pharmacist or one of two pharmacists completing postdoctoral training in nephrology pharmacotherapy. These patients were asked to bring in their medications every 8 weeks during the 2-year study period. At each PC session, the clinical pharmacist would conduct a one-on-one patient interview, generate a current medication profile, identify and address various MRPs through review of medication, chart, and laboratory data, and provide health care provider and patient education. | Standard of care | 780d |
| 72(Welch et al., 2009) | 2009 | Welch | Cohort | US | 459/336 | Mean±SD IG: 68.8±10.7 CG: 68.9±11.3 | (I)had 2 or more chronic conditions, one of which was considered to be high risk (eg, asthma, atrial fibrillation, cerebrovascular disease, coronary artery disease, chronic kidney disease, diabetes, heart failure, hyperlipidemia); (2)were receiving 5 or more Part Dv-covered medications; (3) were likely to incur at least $4000 in total costs for Part D-covered medications in 2006. |  | Chronic Disease | MTM Services | Usual care | 180d |
| 73(Sellors et al., 2003) | 2003 | Sellors | RCT | CA | 431/458 | Mean±SD IG: 74±6.1 CG: 74±6 | Patients were eligible for inclusion in our study if they were aged 65 years or more, taking 5 or more medications, had been seen by their physician within the past 12 months, had no evidence of cognitive impairment and could understand English. |  | Undefined | Face-to-face medication reviews with the patients and then gave written recommendations to the physicians to resolve any drug-related problems. | Usual care | 150d |
| 74(Taylor et al., 2003) | 2003 | Taylor | RCT | US | 33/36 | Mean±SD IG: 64.4±13.7 CG: 66.7±12.3 | Adult patients(18 years or older) who received care at the participating clinics and were identified as being at high risk for medication-related adverse events were enrolled after giving informed. |  | Undefined | Pharmacists joined to provide pharmaceutical care at the clinics two or three after noons per week. Since the clinics did not have a pharmacy, interventions were limited to clinical services and patient education and did not include dispensing. However, patients were asked to bring all their current medications on follow-up visits, and the pharmacists contacted local pharmacies for dispensing information as necessary. | Standard medical care | 360d |
| 75(Chrischilles et al., 2004) | 2003 | Chrischilles | Cohort | US | 524/1687 | Mean±SE IG: 54.1±0.8 CG: 48.4±0.5 | Noninstitutionalized Iowa Medicaid patients taking four or more long-term medications, including at least one medication representing 1 of specified 12 diseases (n = 3,037), were eligible (the 12 diseases were congestive heart disease, ischemic heart disease, diabetes mellitus, hypertension, hyperlipidemia, asthma, depression, atrial fibrillation, osteoarthritis, gastroesophageal reflux, pepticulcer disease, and chronic obstructive pulmonary disease). |  | Chronic Disease | Pharmaceutical case management, included initial patient assessment, written recommendations to physician, follow-up assessments and communication of progress and new problems to physician. | Usual care |  |
| 76(Krska et al., 2001) | 2001 | Krska | RCT | UK | 168/164 | Mean±SD IG: 74.8±6.2 CG: 75.2±6.6 | All Grampian medical practices that had at least 500 patients aged 65 years or over were stratified into three levels by the deprivation status (Jarman index) of their practice population and by fundholding status (yes/no). |  | Undefined | A pharmaceutical care plan was drawn up for each intervention group patient, listing all potential and actual PCIs, together with the desired outputs, the actions planned to achieve the outputs and the outcomes of any potential PCIs already resolved by the pharmacist. The pharmacists implemented all remaining agreed actions, assisted by other practice staff where appropriate. | Usual care | 90d |
| 77(Grymonpre et al., 2011) | 2001 | Grymonpre | RCT | CA | 69/66 | Mean±SD IG: 76.9±8.4 CG: 77.2±8.8 | 65 years or older, non-institutionalised and taking  two or more prescribed or non-prescribed medications. |  | Undefined | A pharmacist conducted a comprehensive drug therapy review on test clients, then addressed issues with the client and/or the client’s physician, with follow-up as required. | Usual care |  |
| 78(Malone et al., 2000) | 2000 | Malone | RCT | US | 523/531 | Mean±SD IG: 66.8±10.2 CG: 66.6±10 | Patients were considered at high risk for drug-related problems if they met ≥3 of the following criteria: (1) were taking ≥5 medications, (2) were taking ≥12 doses per day, (3) had ≥3 chronic medical conditions, (4) had ≥4 changes to their drug regimen over the past year, (5) were taking <80% of their prescribed chronic scheduled medicines based on pharmacy refill records, or (6) were taking a medication requiring therapeutic drug monitoring |  | Undefined | Each patient should have at least three visits with the clinical pharmacist during the study, but patients could be seen as frequently as deemed necessary to ensure appropriate care. Visits were to occur between or concurrent with appointments with the primary care provider or other physicians. Each contact was recorded on a standard data collection form that contained information about the method of contact, estimated time spent with the patient, medical problems addressed, drug-related problems addressed, and drug-related problems resolved. | Usual medical care | 360d |
| 79(Gattis et al., 1999) | 1999 | Gattis | RCT | US | 90/91 | Mean, IQR IG: 71.5 (60-77) CG: 63 (55-72) | Patients with diabetes (n = 127) who participated in an MTM demonstration project and had MTM visits to any Fairview clinic offering MTM services between January 1, 2007, and December 31, 2007 were identified in the Fairview electronic medical record system. | Diabetes | Chronic Disease | Clinical pharmacist evaluation, including medication evaluation, therapeutic recommendations to the attending physician, patient education and follow-up telemonitoring | Usual care | 360d |
| 80(Cowper et al., 1998) | 1998 | Cowper | RCT | US | 105/103 | Mean±SD IG: 69.7±3.5 CG: 69.9±4.1 | Patients were eligible for study if they were 65 years or older, had evidence of polypharmacy (operationally defined as a patient who was prescribed five or more regularly scheduled medications by a VA physician), and received primary care in the GMC. |  | Undefined | The pharmacist followed a protocol based on principles of pharmaceutical care and shown to influence physician behavior. Using the protocol described for the randomization visit, the pharmacist developed written, rank-ordered, patient-specific drug therapy recommendations for the GMC physician. She then discussed these recommendations in a manner designed to be brief (no more than 3 suggestions or 10 minutes), interactive, and noncritical. | Usual care | 360d |
| 81(Hanlon et al., 1996) | 1996 | Hanlon | RCT | US | 105/103 | Mean±SD IG: 69.7±3.5 CG: 69.9±4.1 | Patients were eligible for study if they were 65 years or older, had evidence of polypharmacy (operationally defined as a patient who was prescribed five or more regularly scheduled medications by a VA physician), and received primary care in the GMC. |  | Undefined | The clinical pharmacist intervention was based on the principles of pharmaceutical care, “a process through which pharmacists cooperate with patients and other health care professionals in designing, implementing,  and monitoring a therapeutic plan that will produce specific therapeutic outcomes for the patient.“ | Usual care | 360d |

**Supplementary Table 1.** Baseline of included studies

| **Clinical outcomes** | **Egger's test** |
| --- | --- |
| Readmission | 0.351 |
| ED visit | 0.022 |
| Mortality | 0.24 |
| ADE-AC | 0.11 |
| ADE-SAE | 0.209 |
| LoS | 0.239 |
| DRP | 0.071 |
| **Economic outcomes** |  |
| Total cost | 0.568 |
| Cost of hospitalization | 0.723 |
| Medication cost | 0.01 |
| **Humanistic outcomes** |  |
| EQ-5D | 0.472 |
| SF-Physical outcomes | 0.214 |
| SF-Mental outcomes | 0.27 |
| MAI | 0.212 |
| Adherence | 0.443 |

**Supplementary Table 2.** Results of Egger’s test.

## Supplementary Figures

### Clinical outcomes


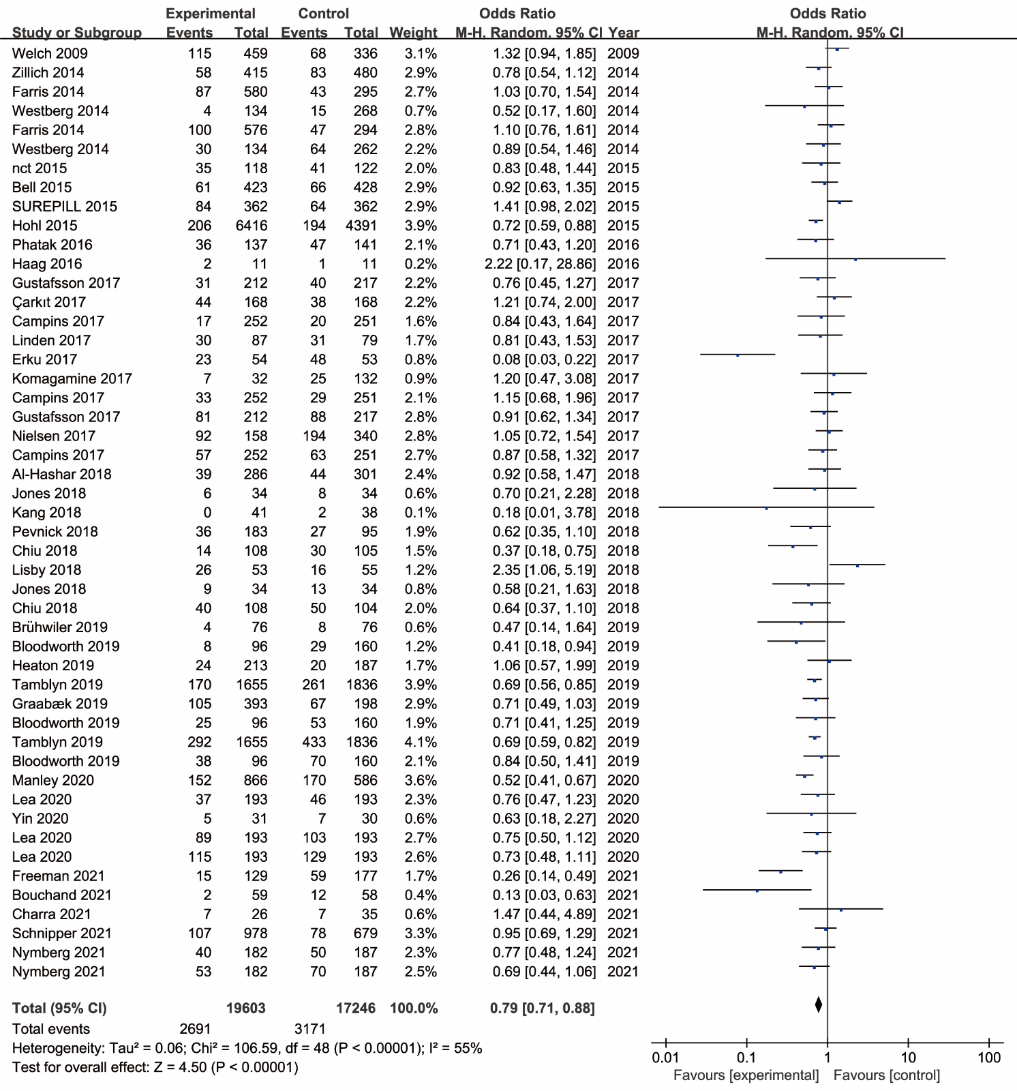


**Supplementary Fig 1.** Forest plot of rate of readmission.


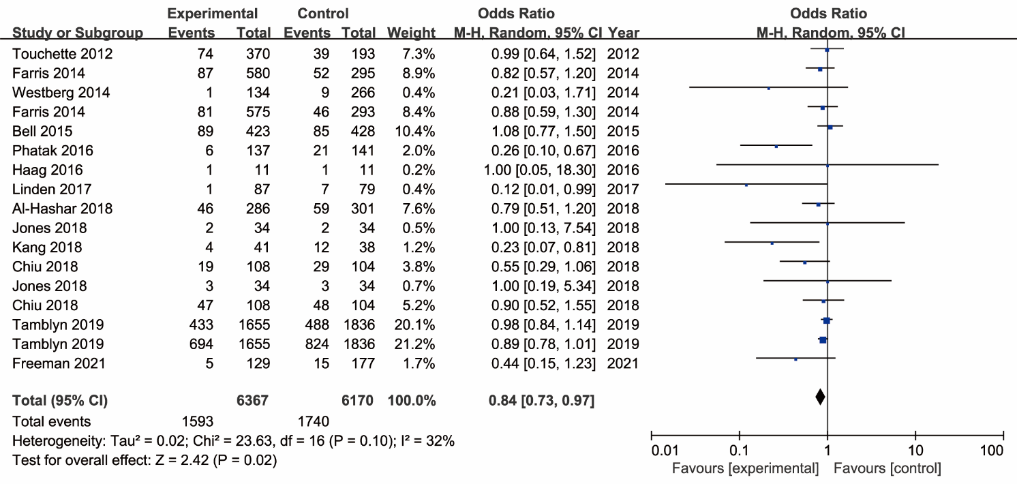


**Supplementary Fig 2.** Forest plot of rate of ED visit.


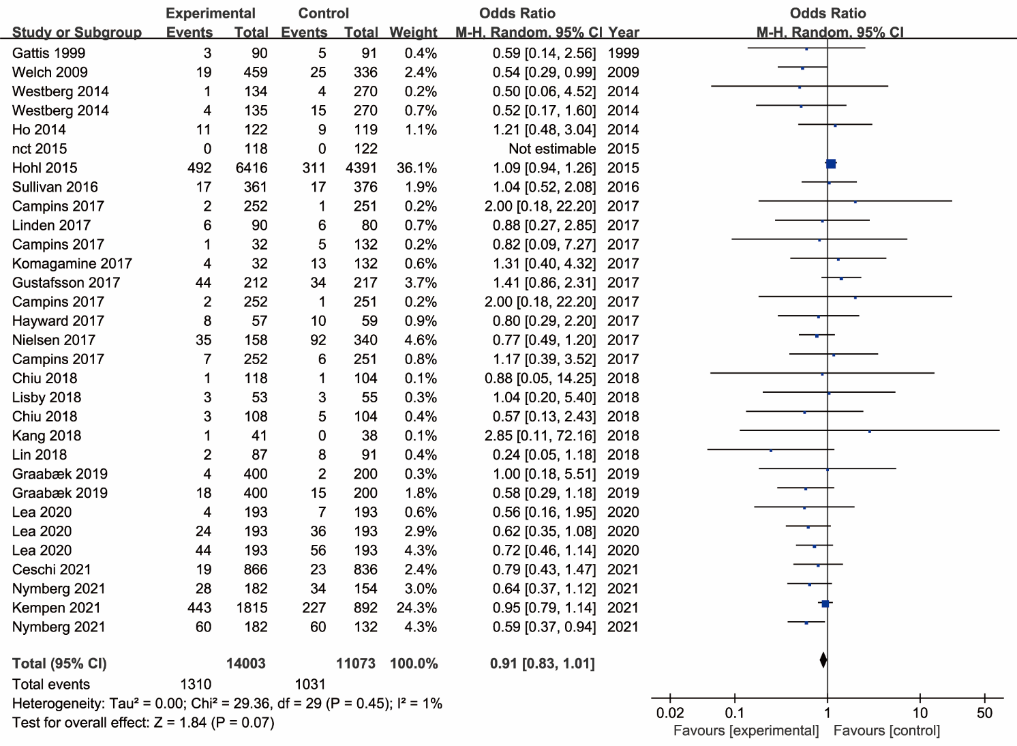


**Supplementary Fig 3.** Forest plot of rate of mortality.


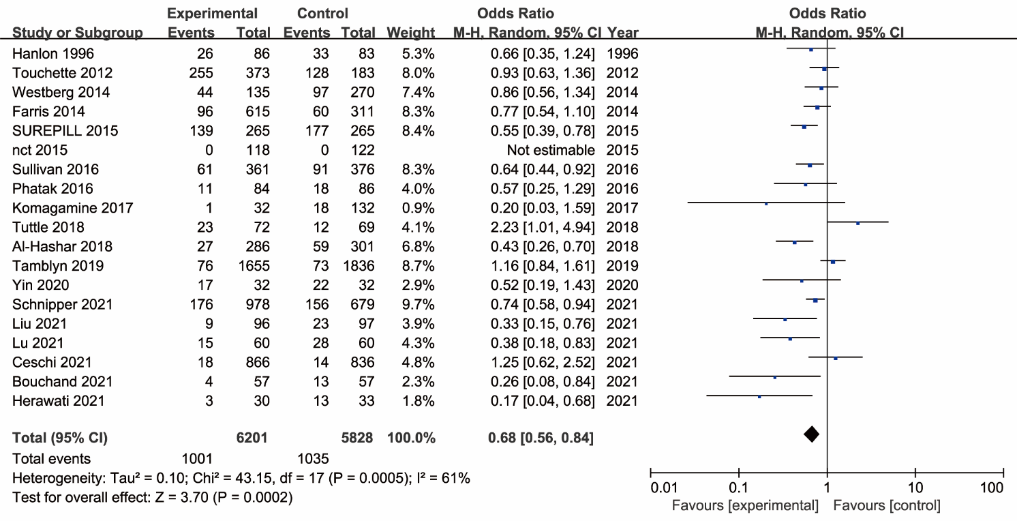


**Supplementary Fig 4.** Forest plot of rate of all cause ADE.


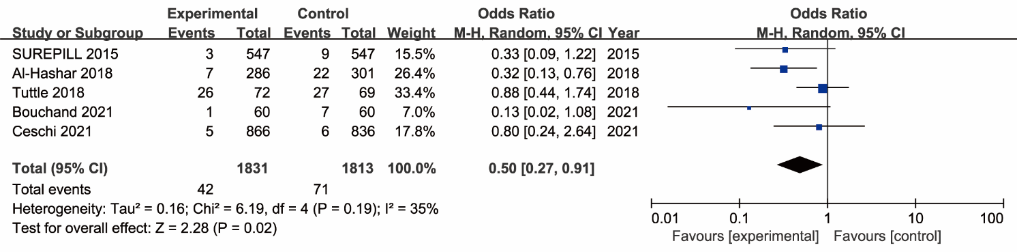


**Supplementary Fig 5.** Forest plot of rate of SAE.


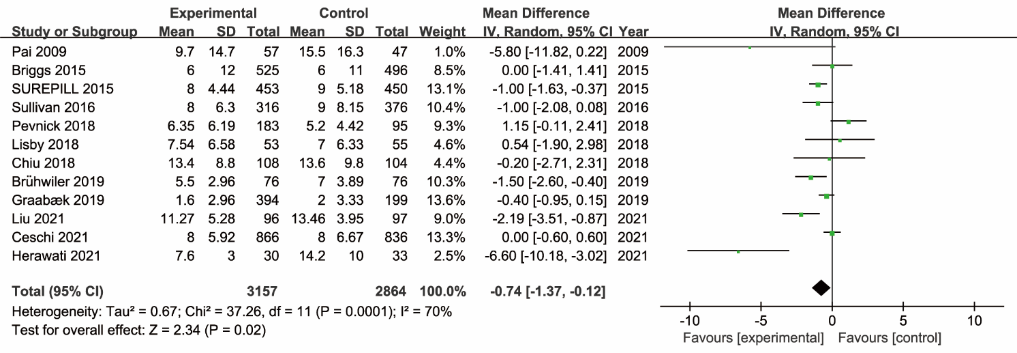


**Supplementary Fig 6.** Forest plot of LoS in hospital.


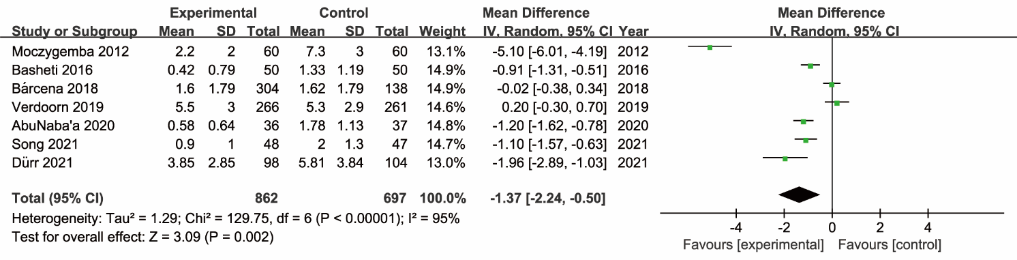


**Supplementary Fig 7.** Forest plot of DRPs.


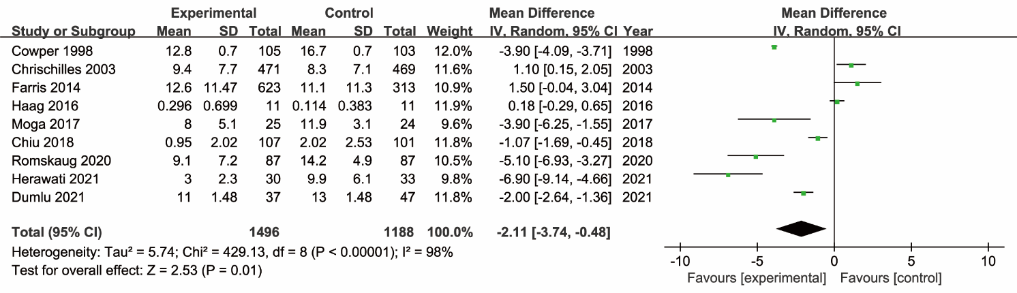


**Supplementary Fig 8.** Forest plot of MAI.

### Economic outcomes


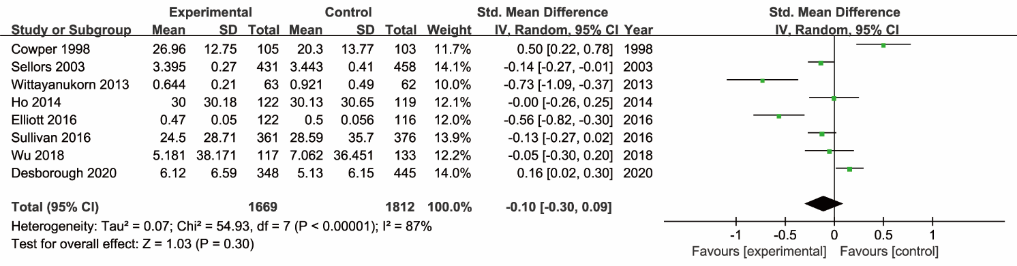


**Supplementary Fig 9.** Forest plot of total cost.


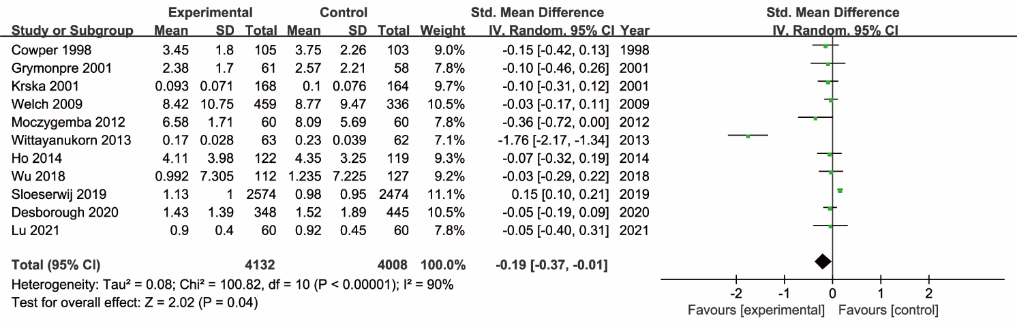


**Supplementary Fig 10.** Forest plot of medication cost.


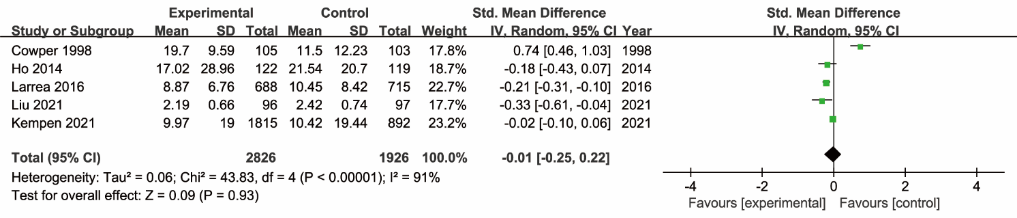


**Supplementary Fig 11.** Forest plot of cost of hospitalization.

### Humanistic outcomes


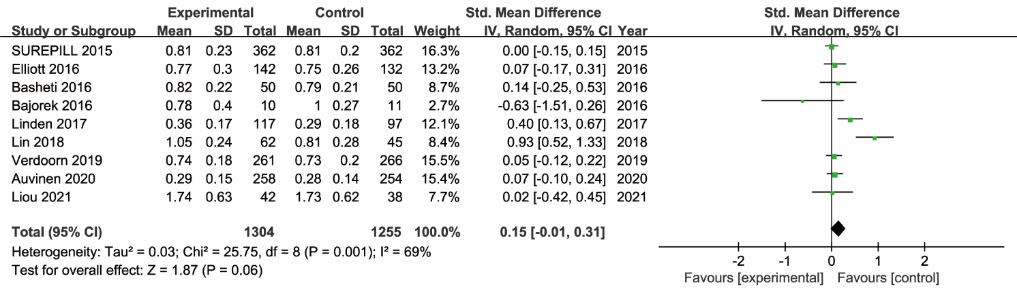


**Supplementary Fig 12.** Forest plot of EQ-5D.


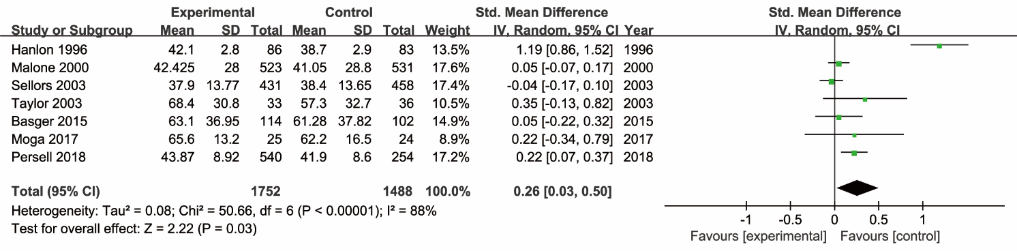


**Supplementary Fig 13.** Forest plot of SF-Physical outcomes.


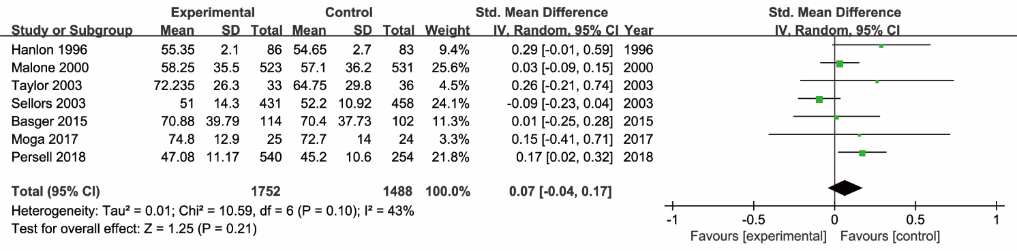


**Supplementary Fig 14.** Forest plot of SF-Mental outcomes.


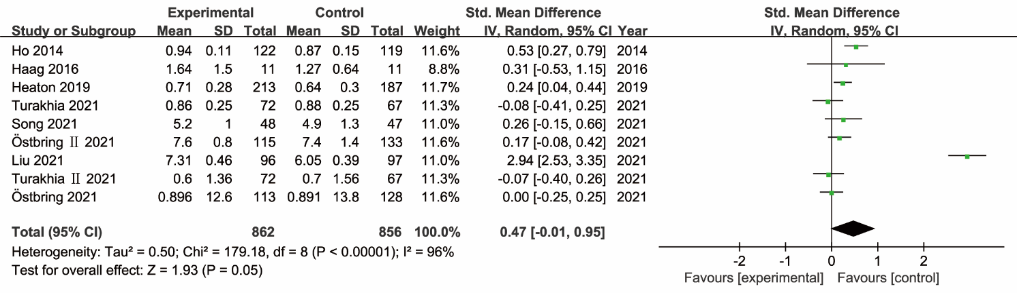


**Supplementary Fig 15.** Forest plot of Adherence.

### Results of subgroup analysis

#### Clinical outcomes


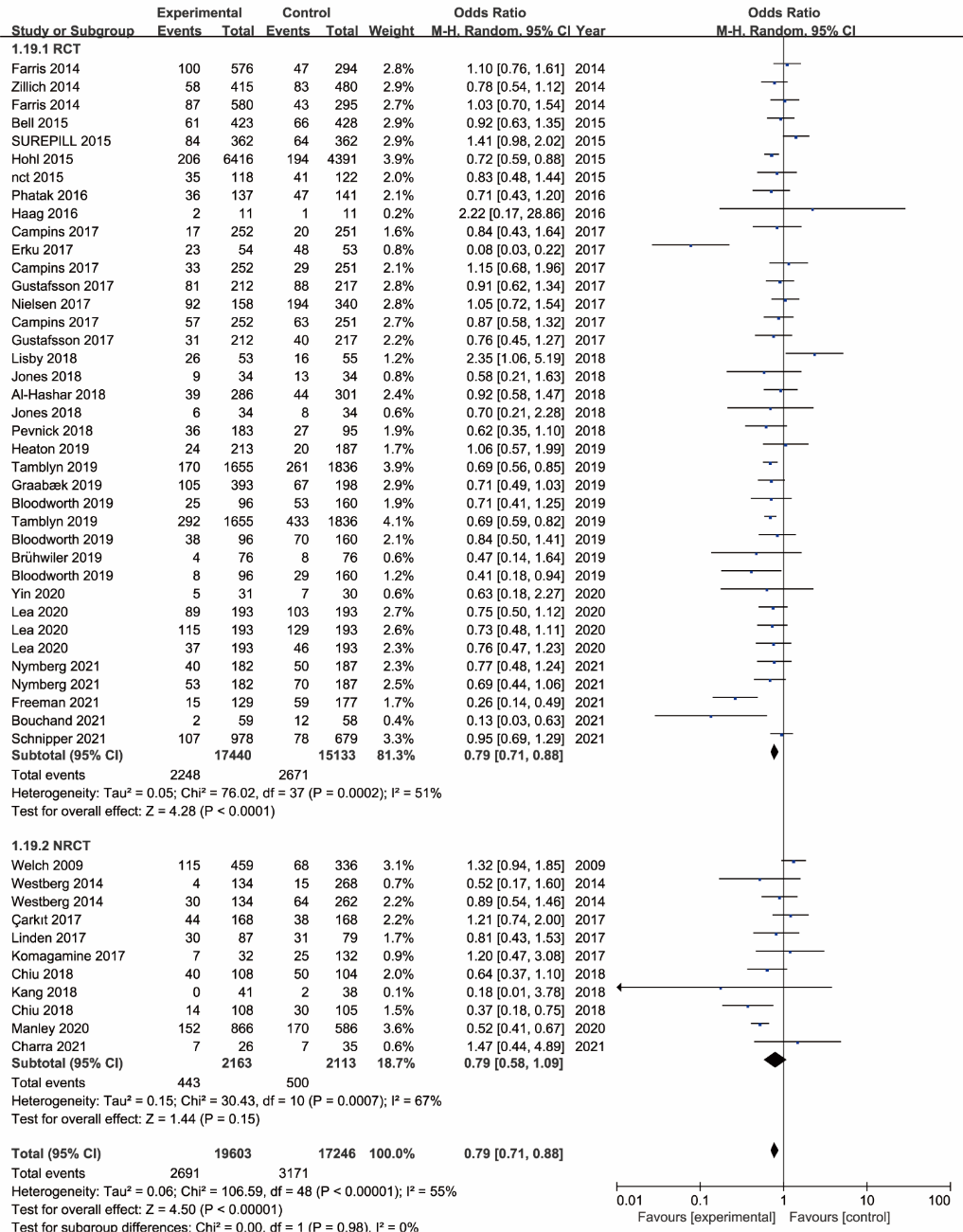


**Supplementary Fig 16.** Subgroup analysis of readmission: Type of studies.


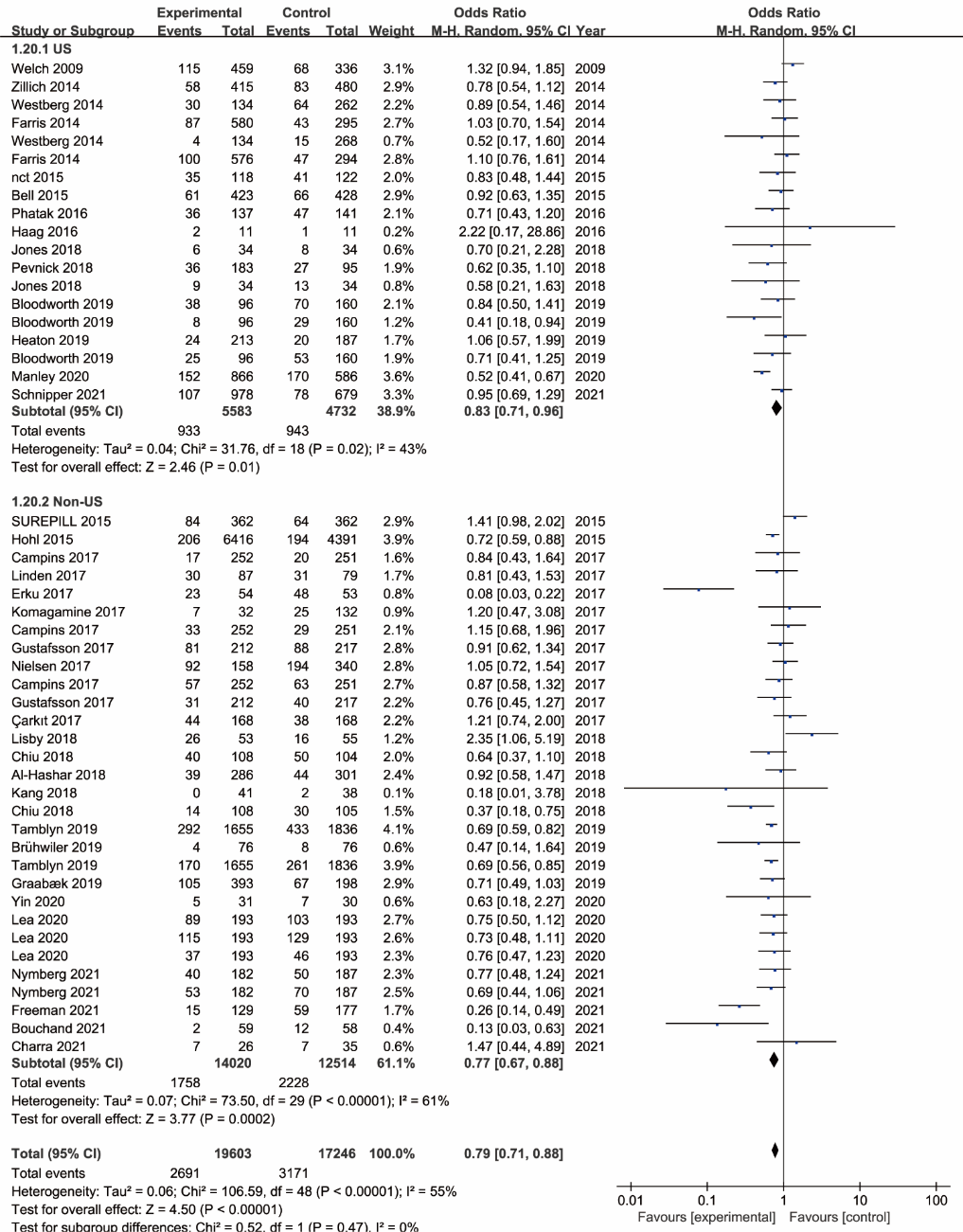


**Supplementary Fig 17.** Subgroup analysis of readmission: District of studies.


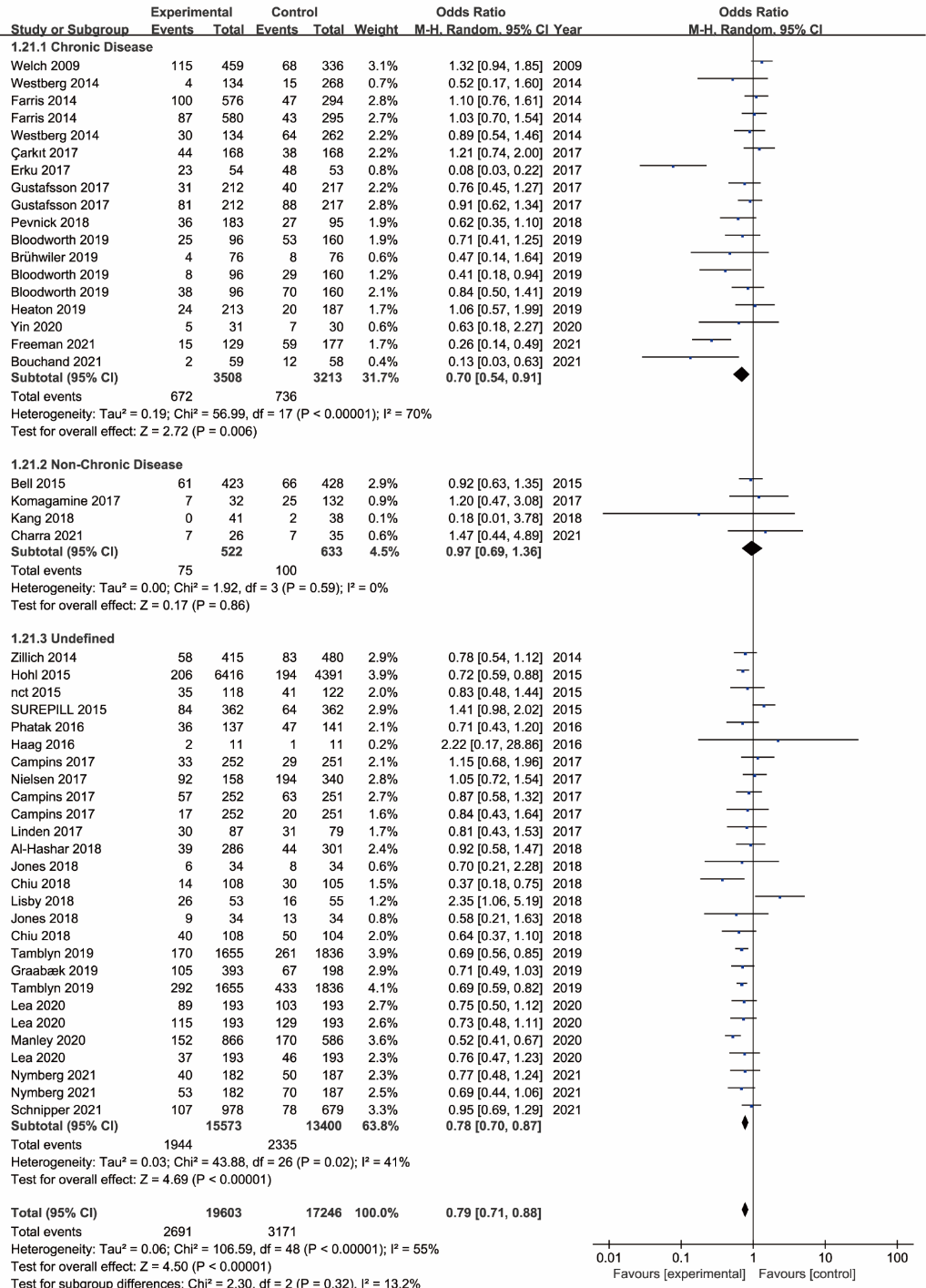


**Supplementary Fig 18.** Subgroup analysis of readmission: Type of diseases.


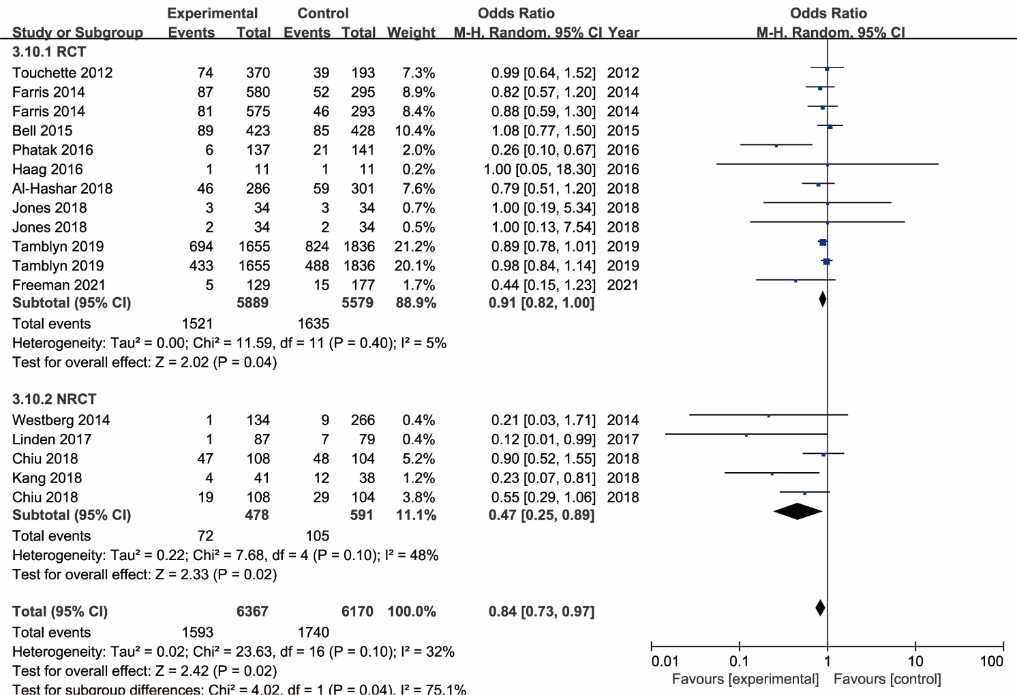


**Supplementary Fig 19.** Subgroup analysis of ED visit: Type of studies.


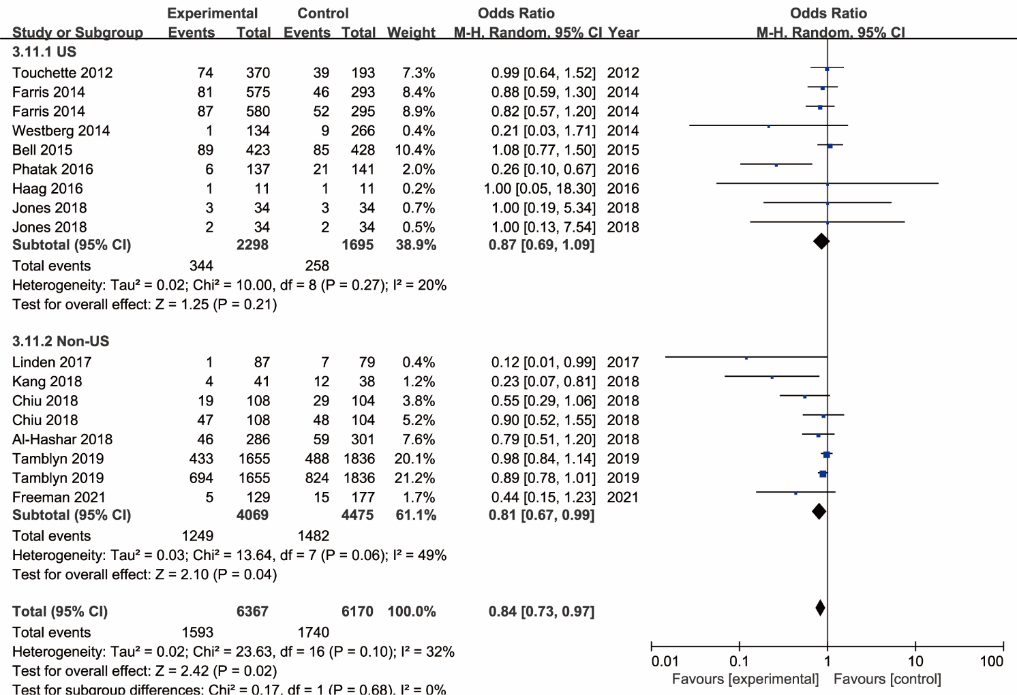


**Supplementary Fig 20.** Subgroup analysis of ED visit: District of studies.


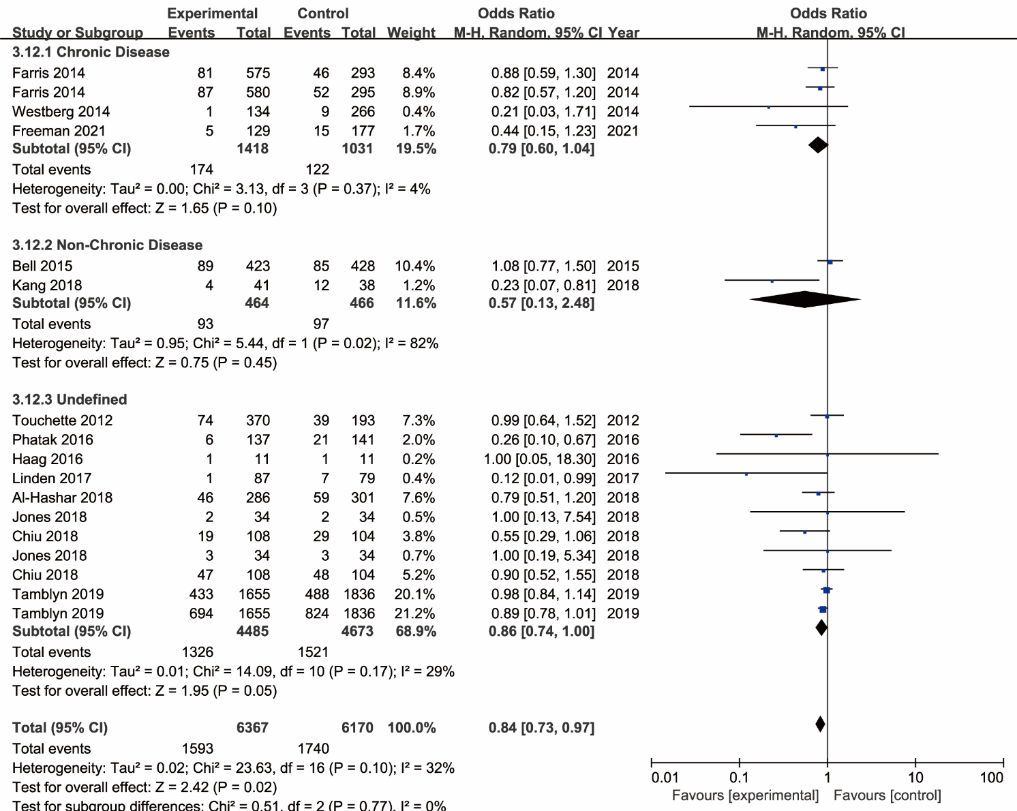


**Supplementary Fig 21.** Subgroup analysis of ED visit: Type of diseases.


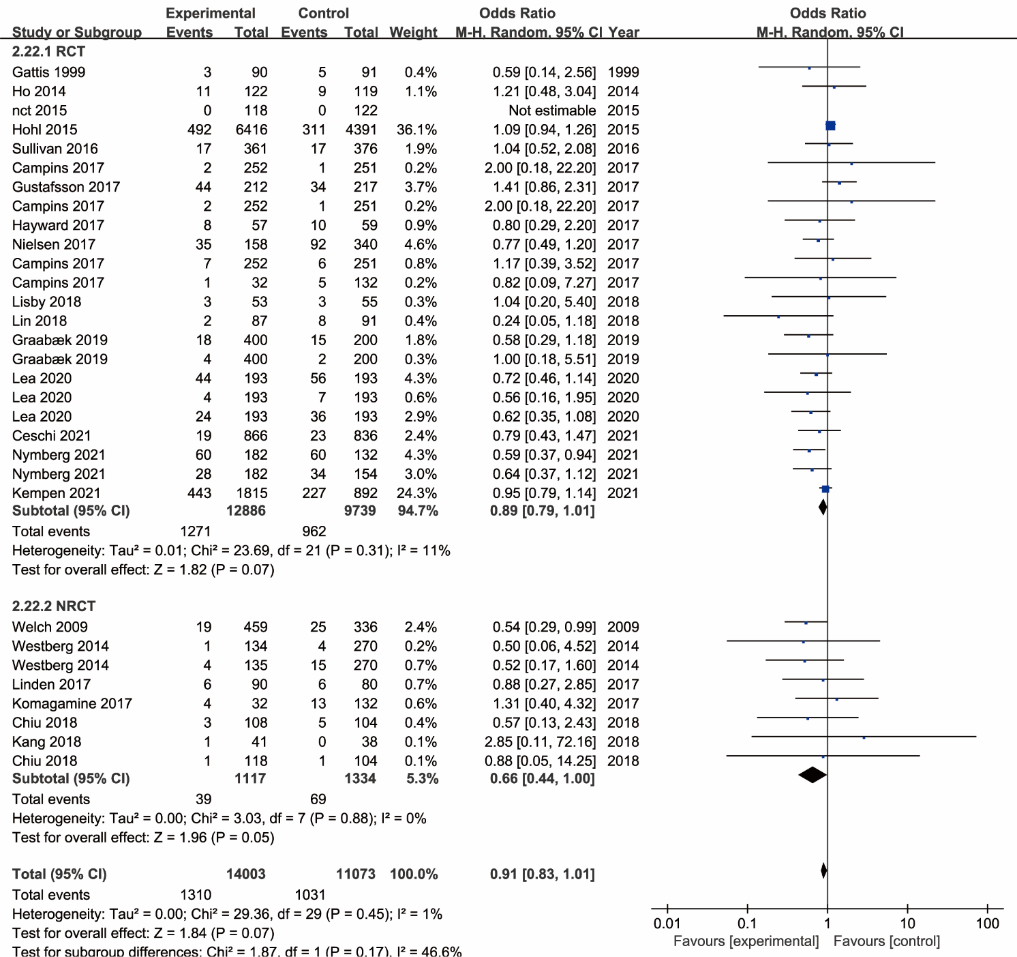


**Supplementary Fig 22.** Subgroup analysis of mortality: Type of studies.


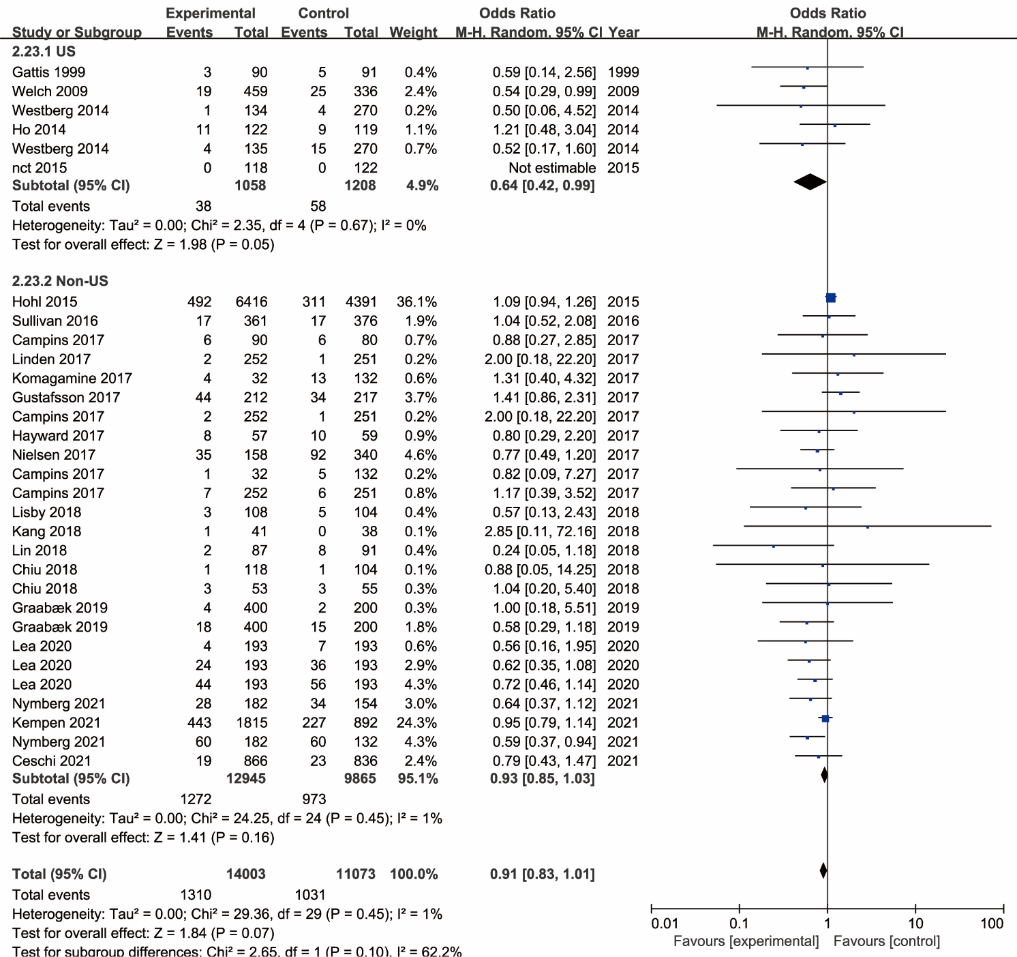


**Supplementary Fig 23.** Subgroup analysis of mortality: District of studies.


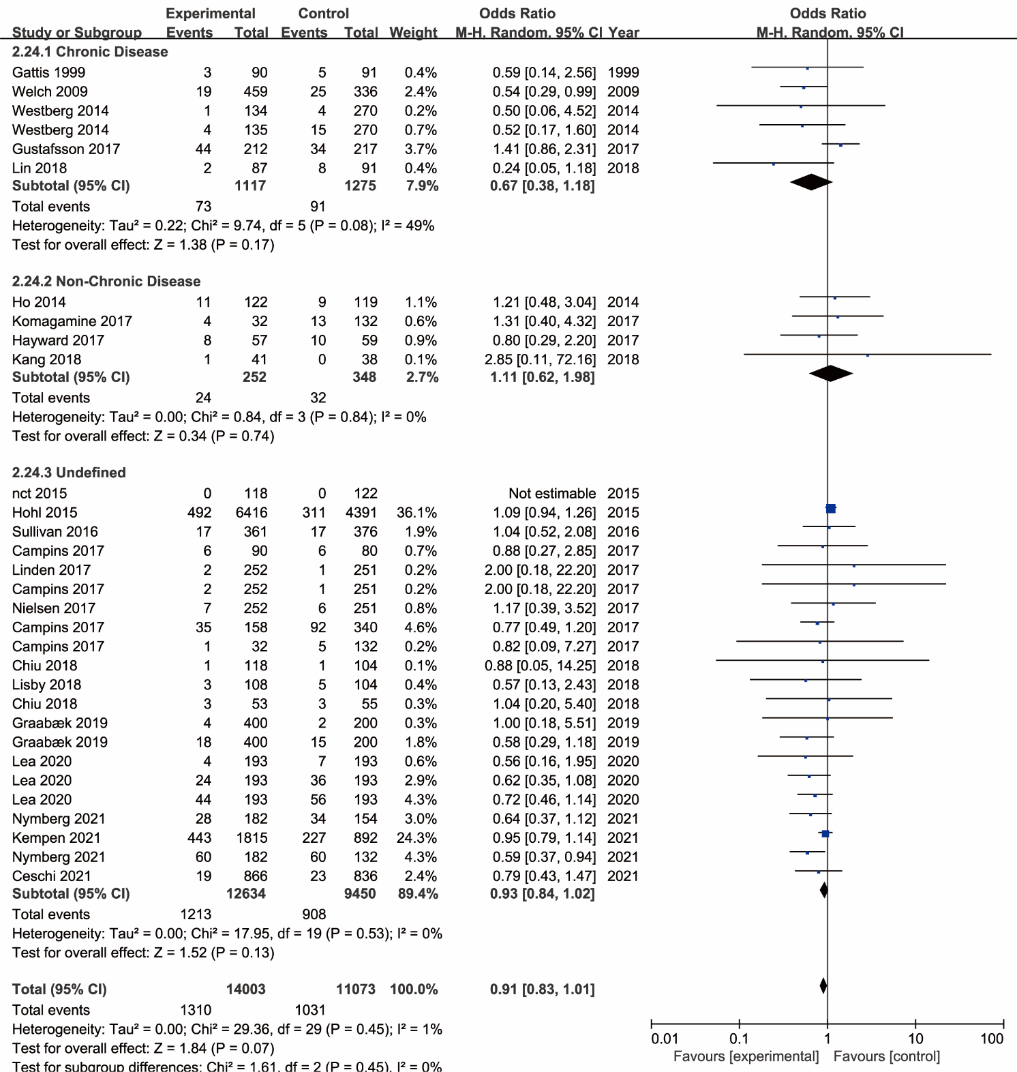


**Supplementary Fig 24.** Subgroup analysis of mortality: Type of diseases.


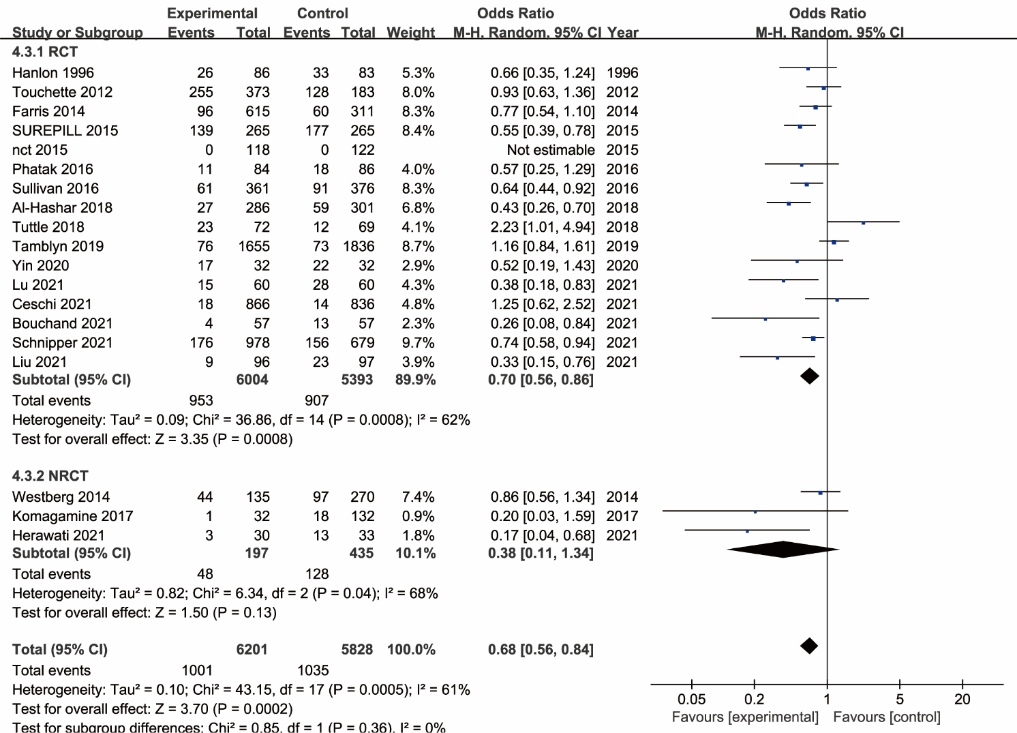


**Supplementary Fig 25.** Subgroup analysis of all cause ADE: Type of studies.


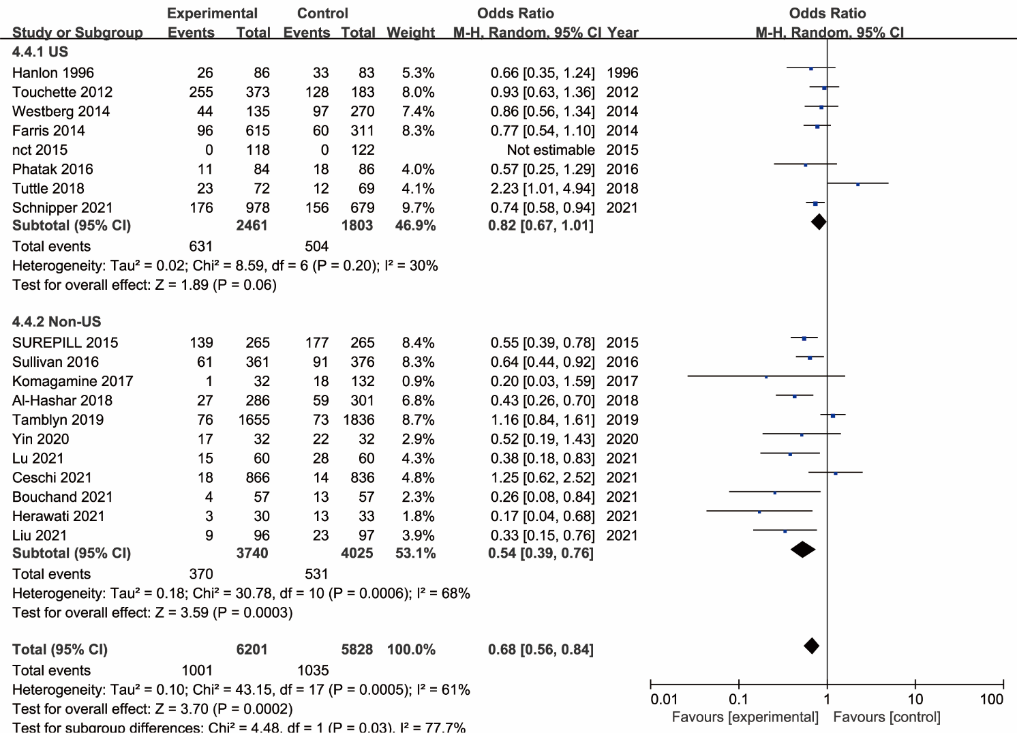


**Supplementary Fig 26.** Subgroup analysis of all cause ADE: District of studies.


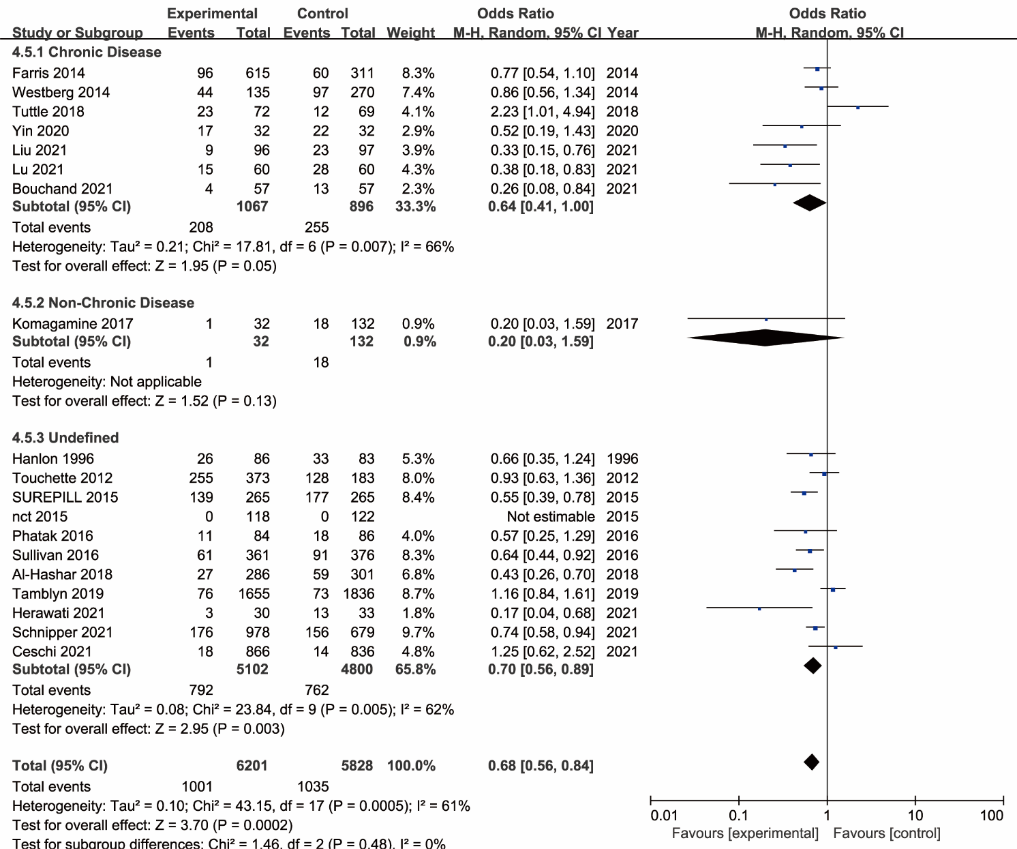


**Supplementary Fig 27.** Subgroup analysis of all cause ADE: Type of diseases.


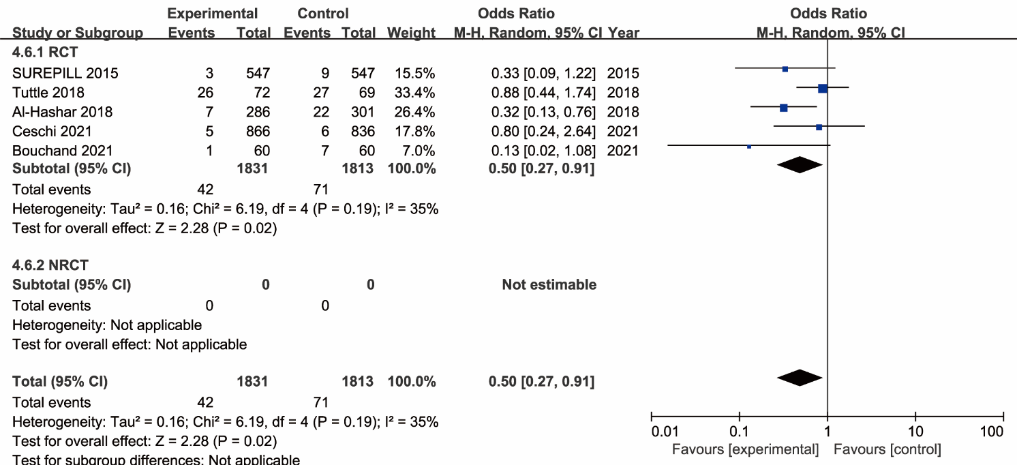


**Supplementary Fig 28.** Subgroup analysis of SAE: Type of studies.


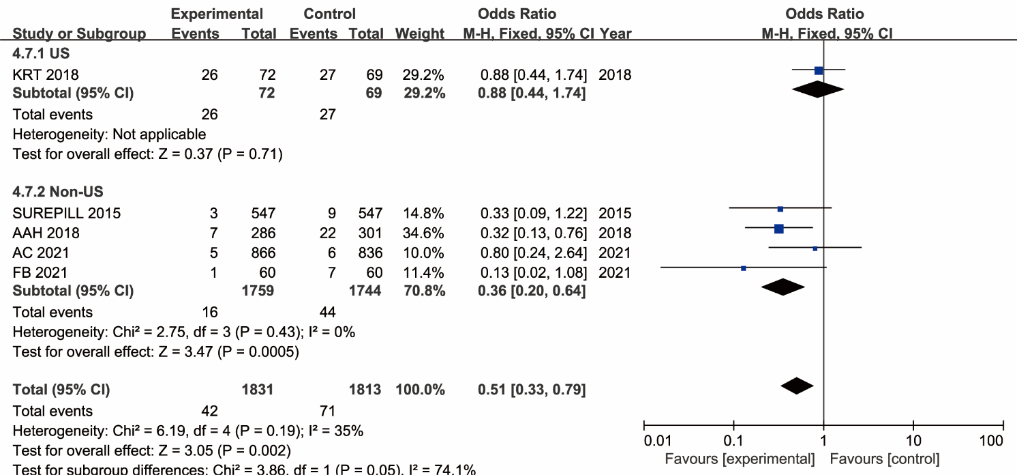


**Supplementary Fig 29.** Subgroup analysis of SAE: District of studies.


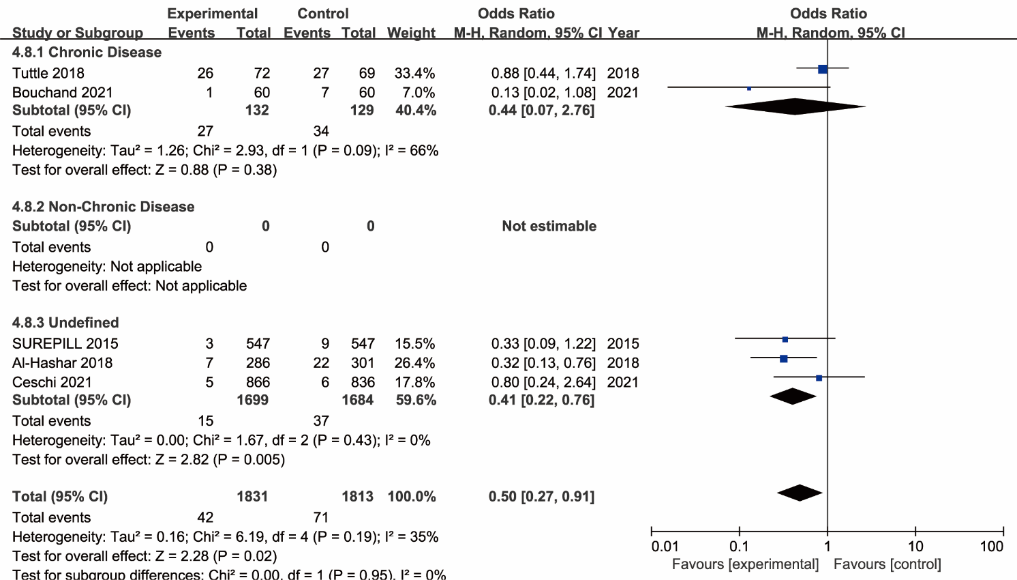


**Supplementary Fig 30.** Subgroup analysis of SAE: Type of diseases.


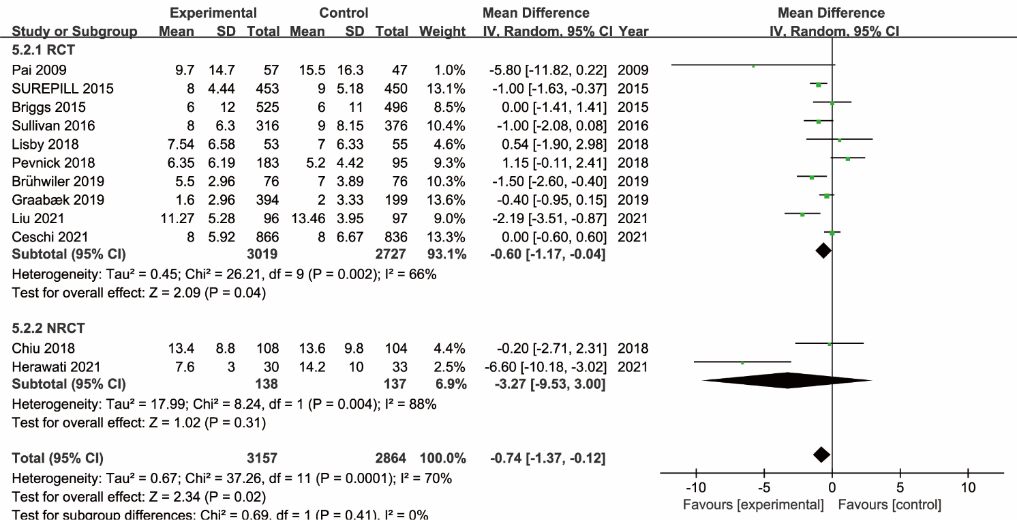


**Supplementary Fig 31.** Subgroup analysis of LoS: Type of studies.


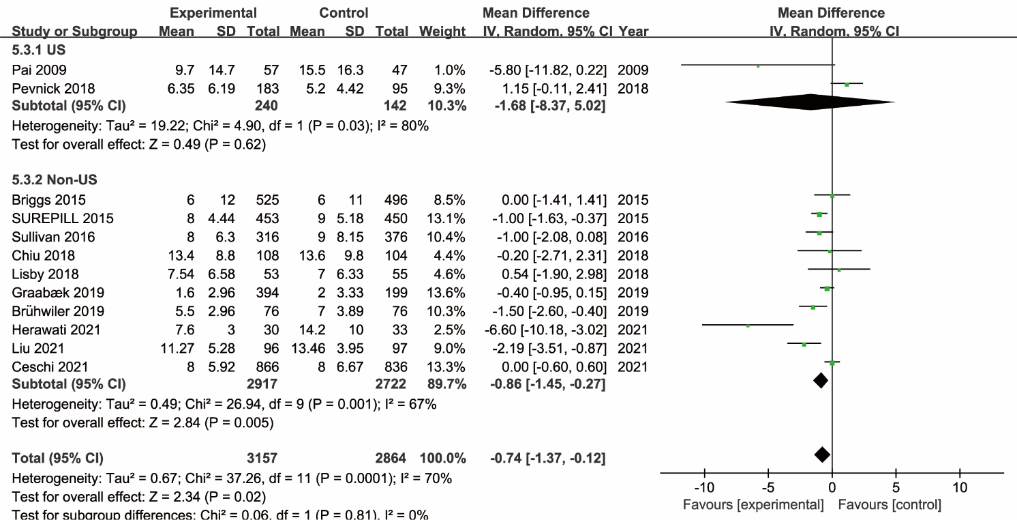


**Supplementary Fig 32.** Subgroup analysis of LoS: District of studies.


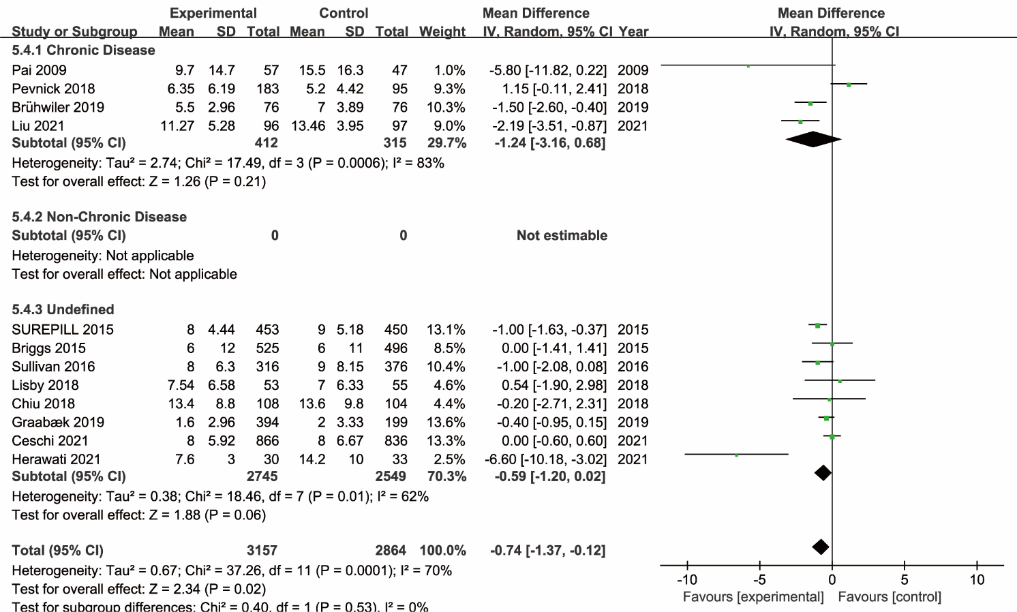


**Supplementary Fig 33.** Subgroup analysis of LoS: Type of diseases.


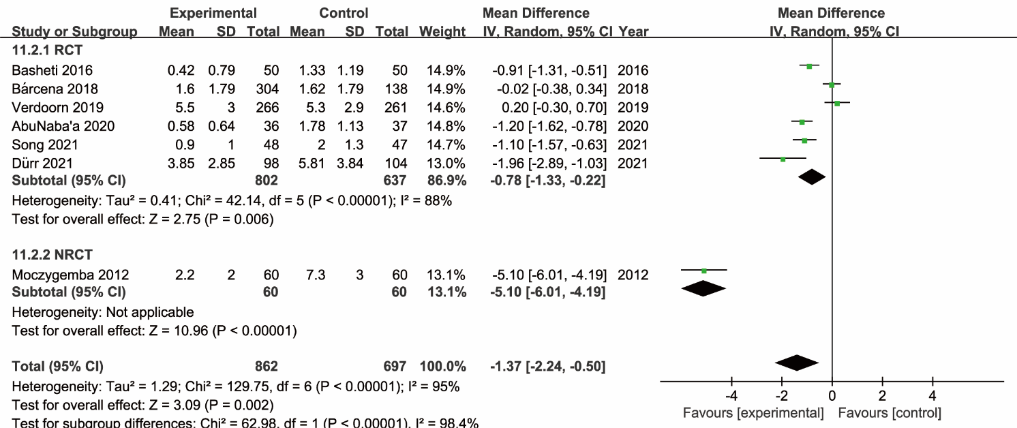


**Supplementary Fig 34.** Subgroup analysis of DRPs: Type of studies.


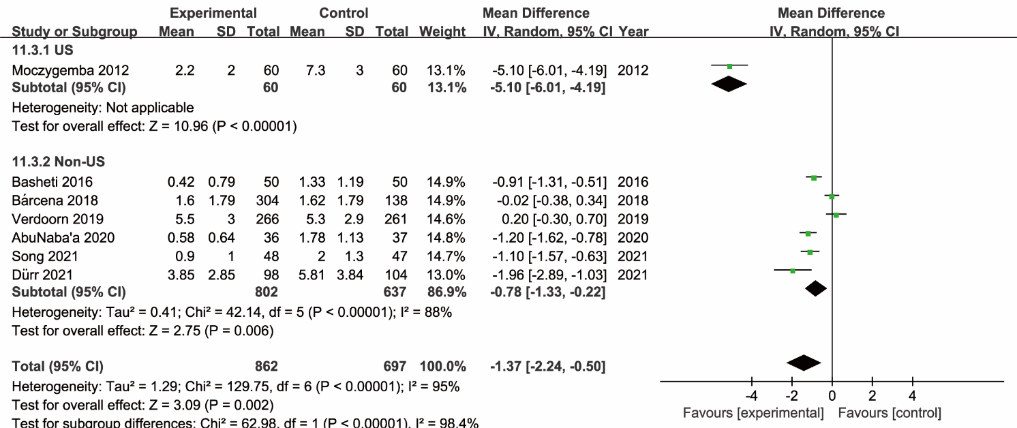


**Supplementary Fig 35.** Subgroup analysis of DRPs: District of studies.


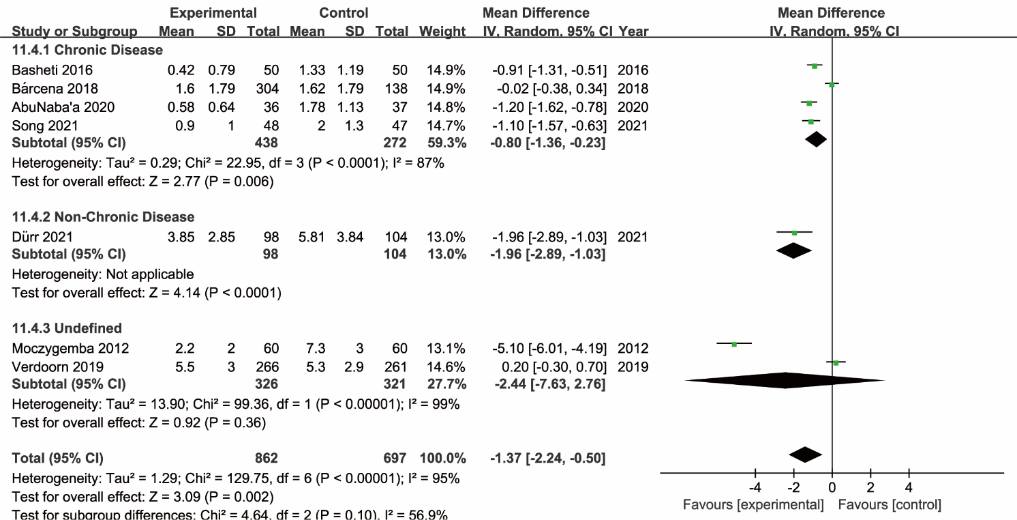


**Supplementary Fig 36.** Subgroup analysis of DRPs: Type of diseases.


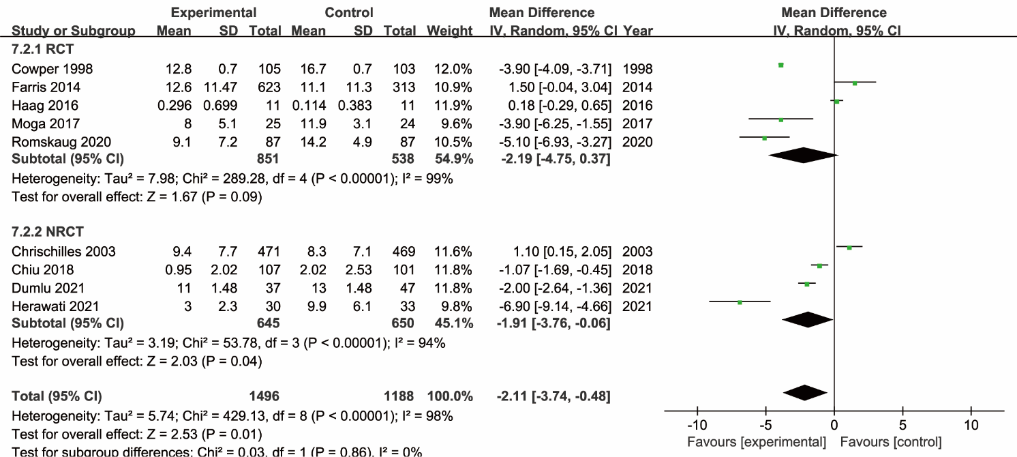


**Supplementary Fig 37.** Subgroup analysis of MAI: Type of studies.


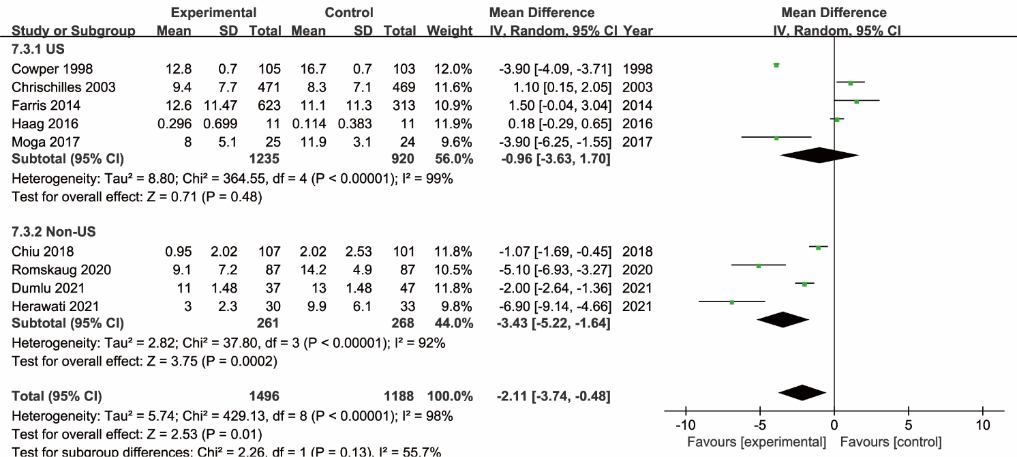


**Supplementary Fig 38.** Subgroup analysis of MAI: District of studies.


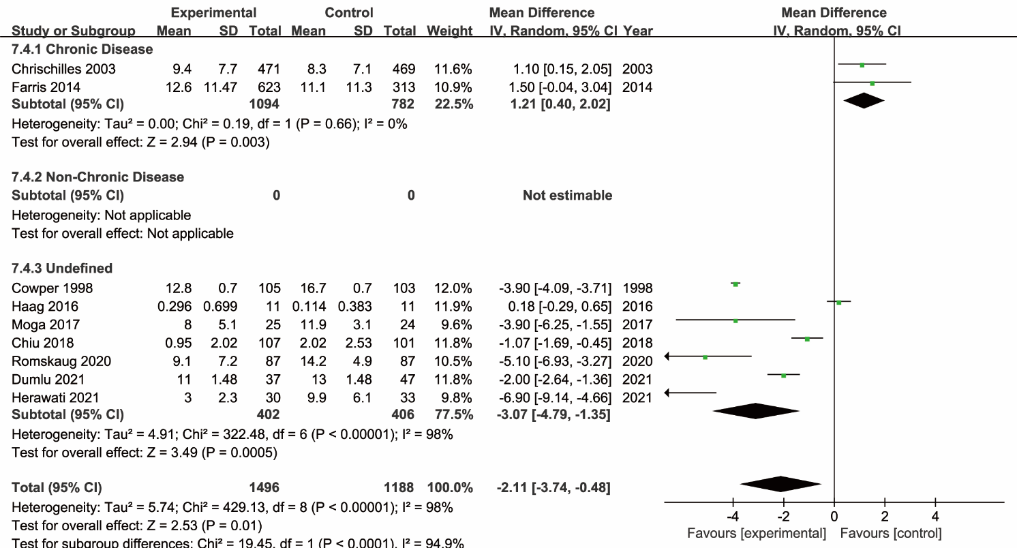


**Supplementary Fig 39.** Subgroup analysis of MAI: Type of diseases.

#### Economic outcomes


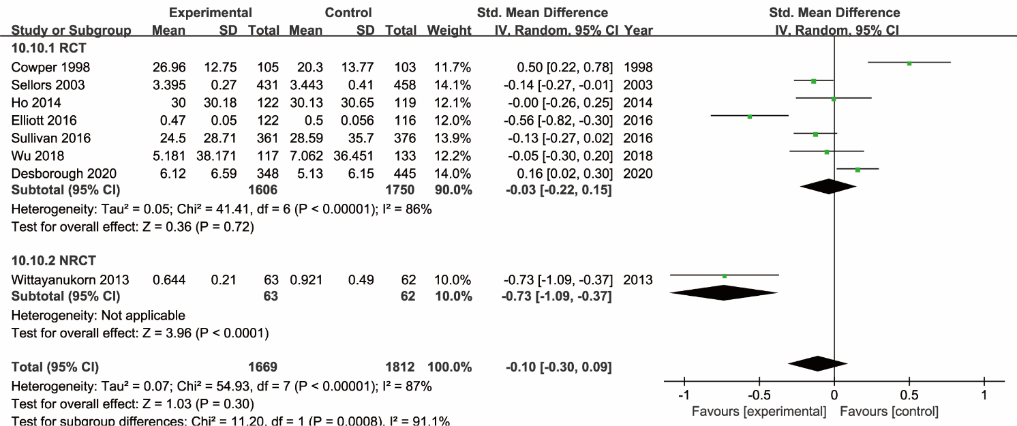


**Supplementary Fig 40.** Subgroup analysis of total cost: Type of studies.


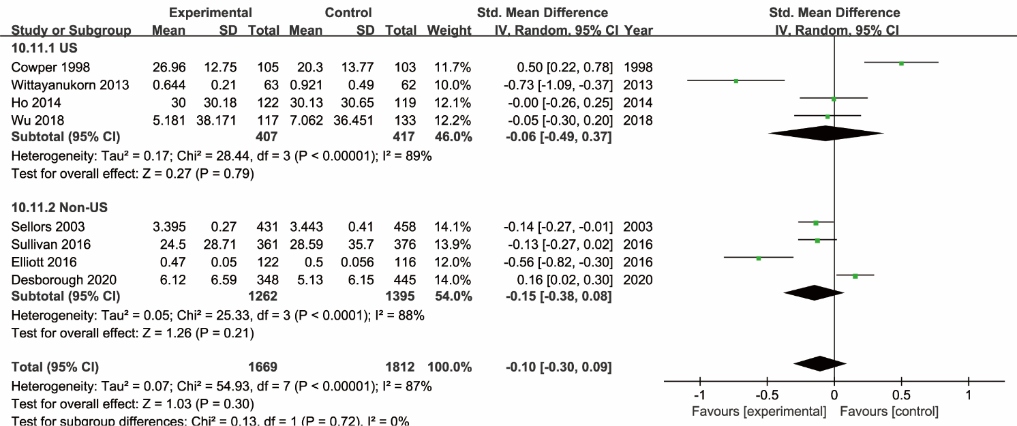


**Supplementary Fig 41.** Subgroup analysis of total cost: District of studies.


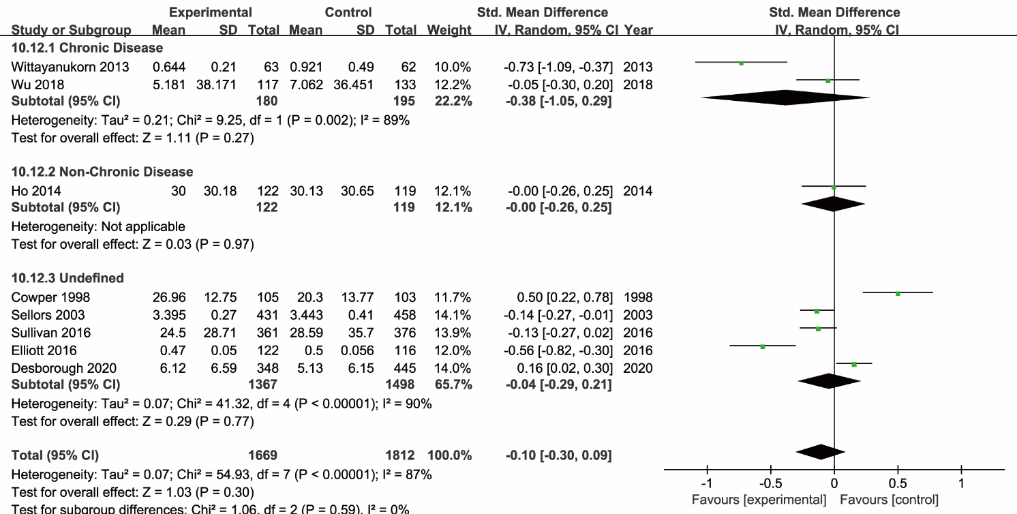


**Supplementary Fig 42.** Subgroup analysis of total cost: Type of diseases.


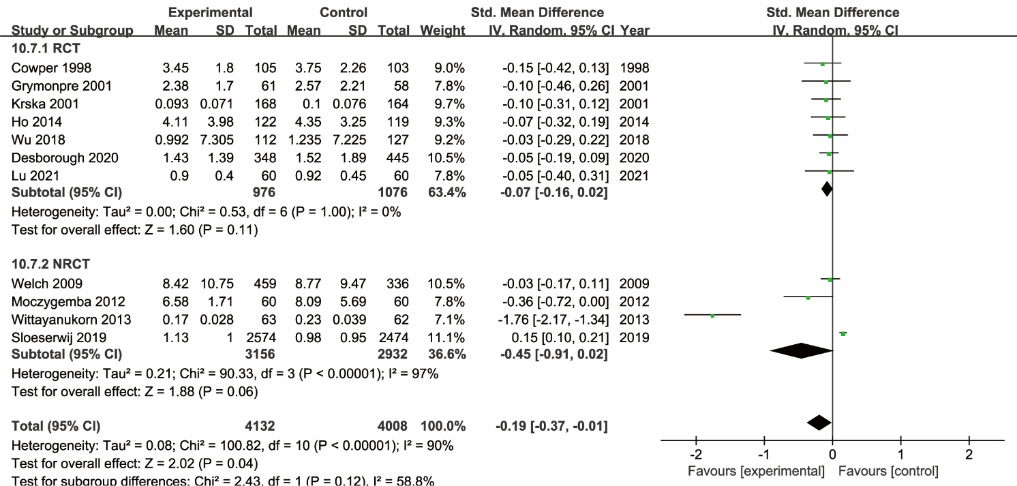


**Supplementary Fig 43.** Subgroup analysis of medication cost: Type of studies.


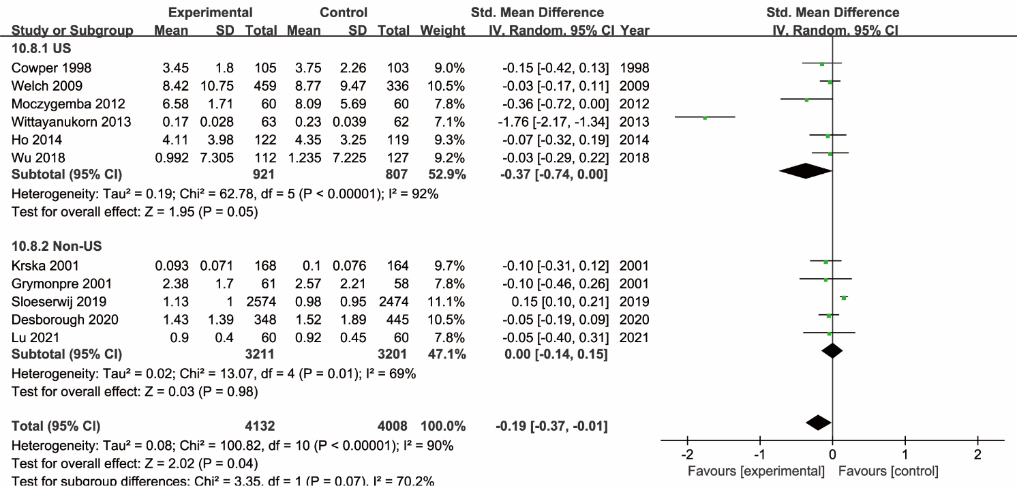


**Supplementary Fig 44.** Subgroup analysis of medication cost: District of studies.


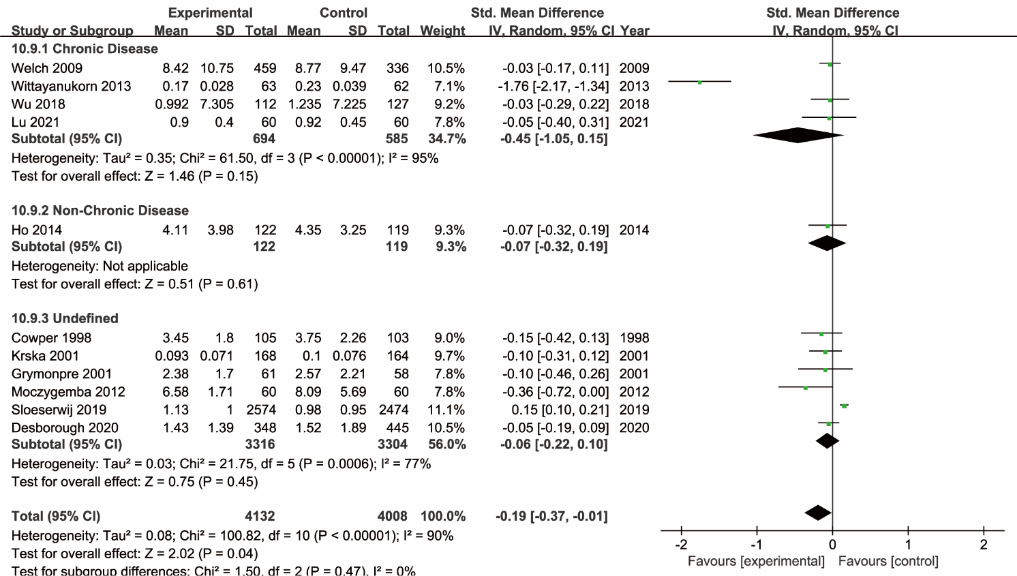


**Supplementary Fig 45.** Subgroup analysis of medication cost: Type of diseases.


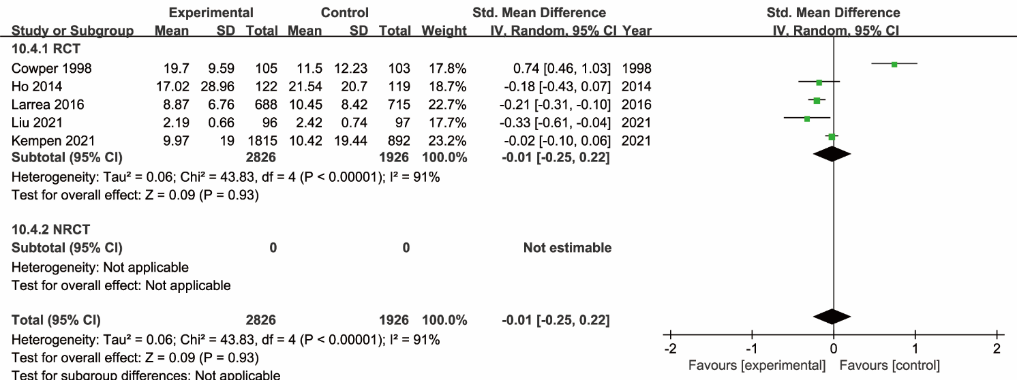


**Supplementary Fig 46.** Subgroup analysis of cost of hospitalization: Type of studies.


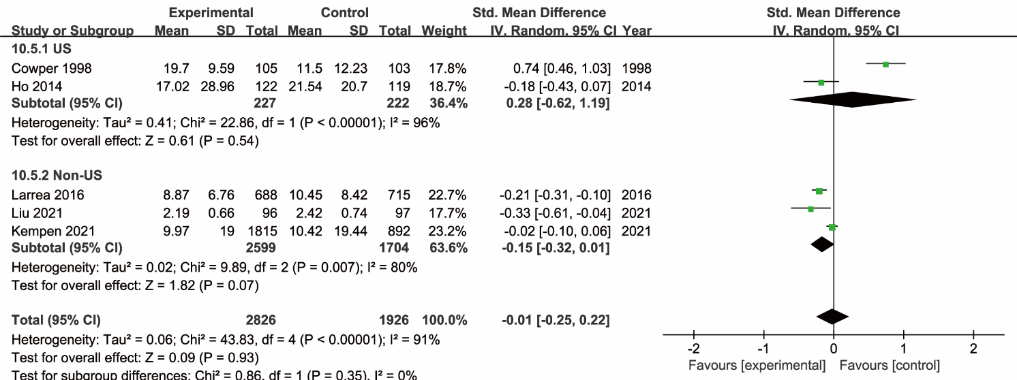


**Supplementary Fig 47.** Subgroup analysis of cost of hospitalization: District of studies.


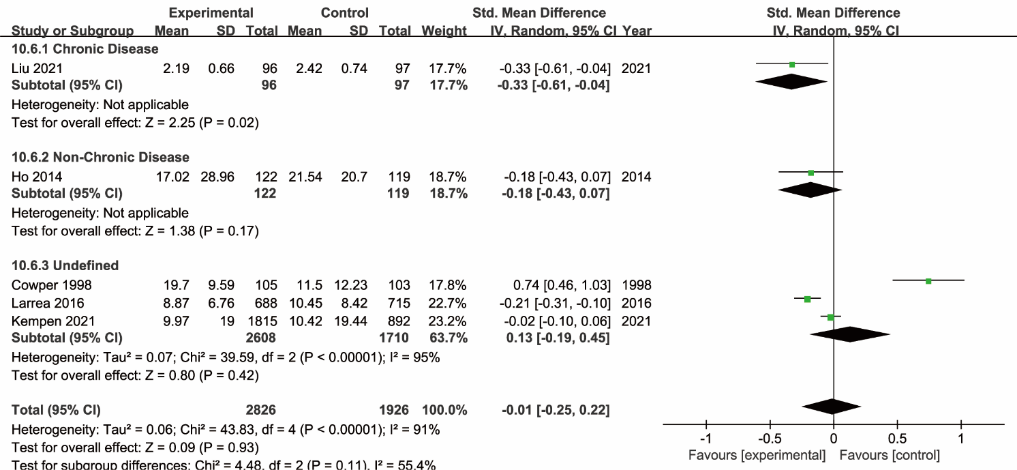


**Supplementary Fig 48.** Subgroup analysis of cost of hospitalization: Type of diseases.

#### Humanistic outcomes


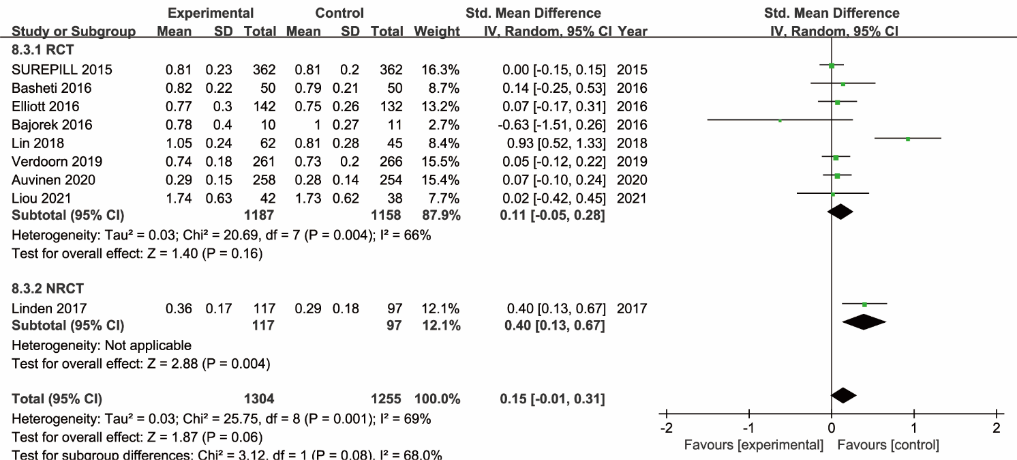


**Supplementary Fig 49.** Subgroup analysis of EQ-5D: Type of studies.


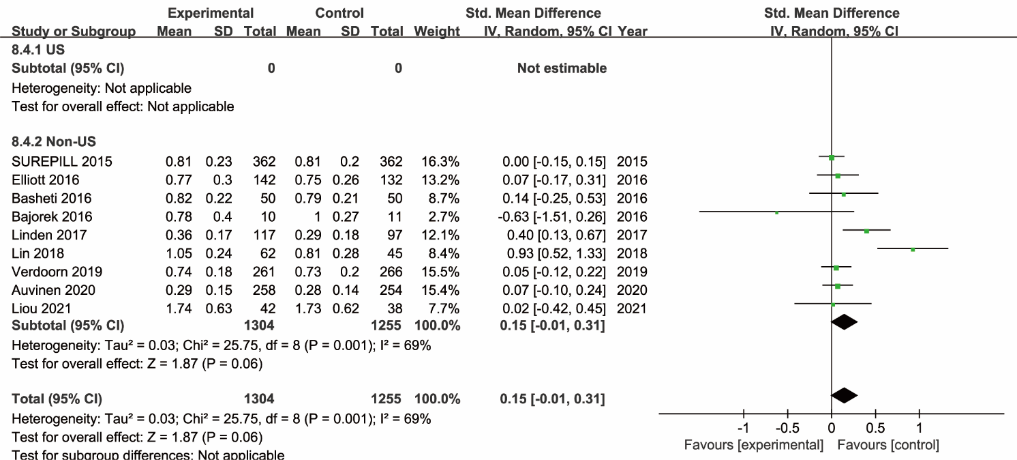


**Supplementary Fig 50.** Subgroup analysis of EQ-5D: District of studies.


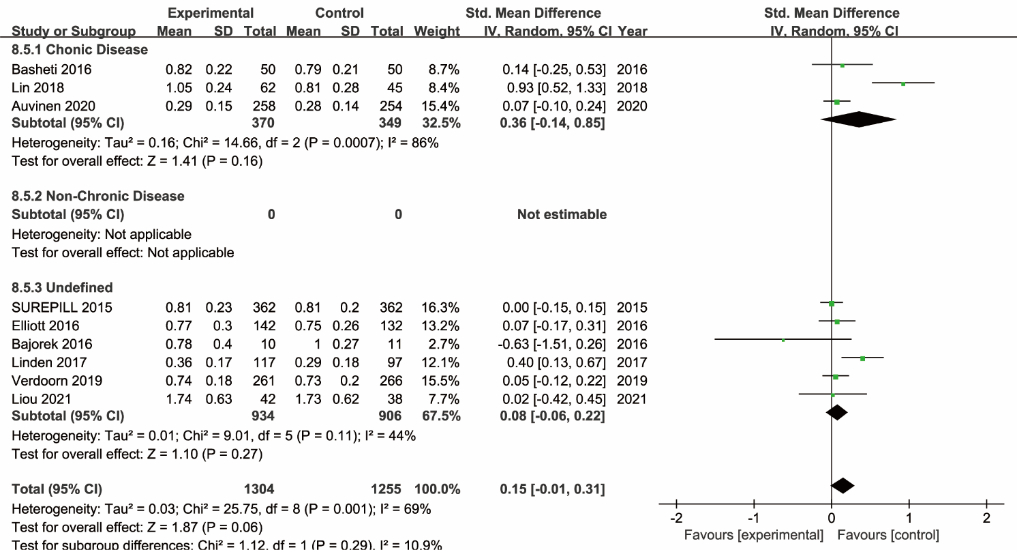


**Supplementary Fig 51.** Subgroup analysis of EQ-5D: Type of diseases.


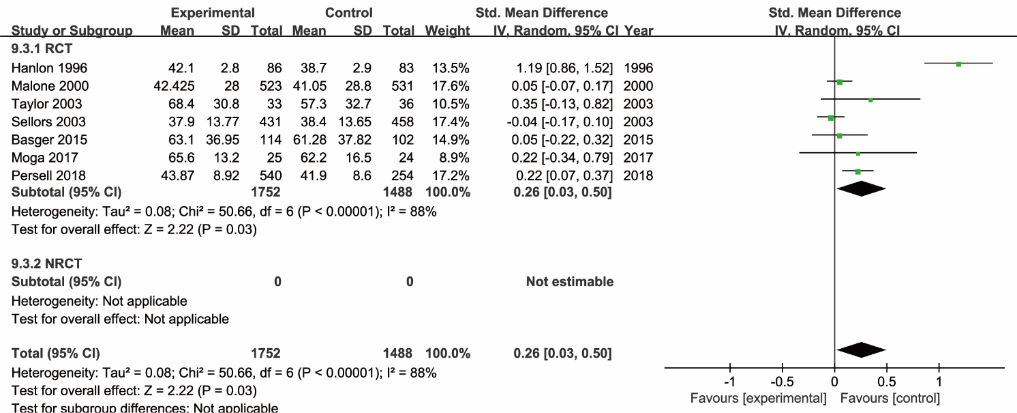


**Supplementary Fig 52.** Subgroup analysis of SF-Physical outcomes: Type of studies.


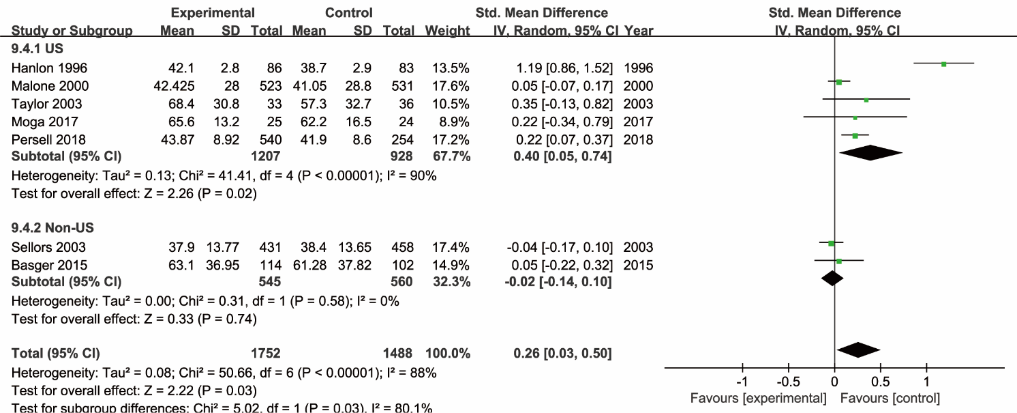


**Supplementary Fig 53.** Subgroup analysis of SF-Physical outcomes: District of studies.


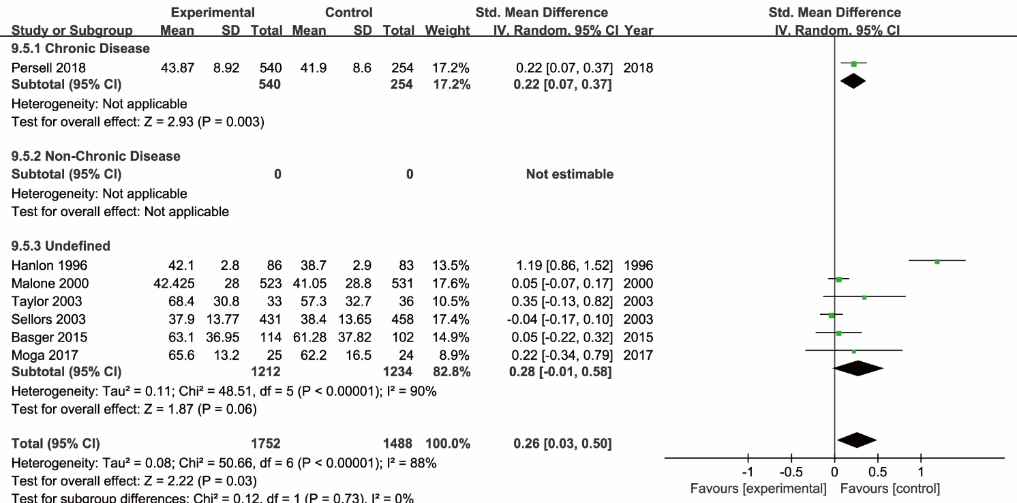


**Supplementary Fig 54.** Subgroup analysis of SF-Physical outcomes: Type of diseases.


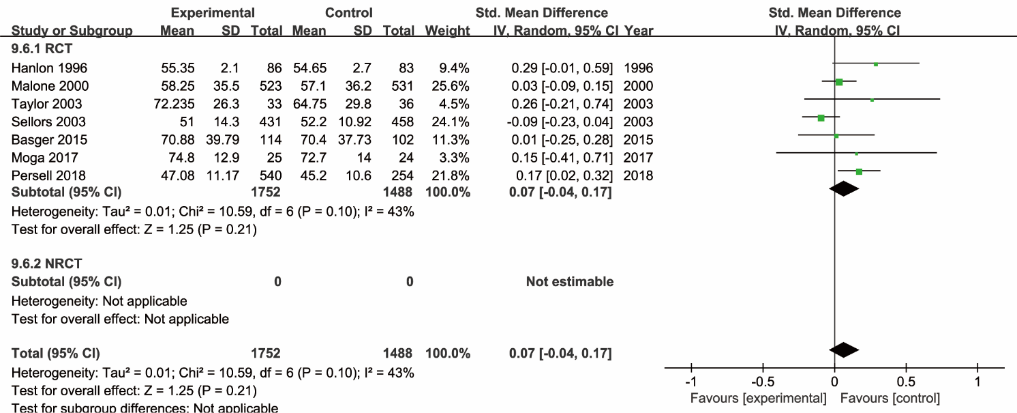


**Supplementary Fig 55.** Subgroup analysis of SF-Mental outcomes: Type of studies.


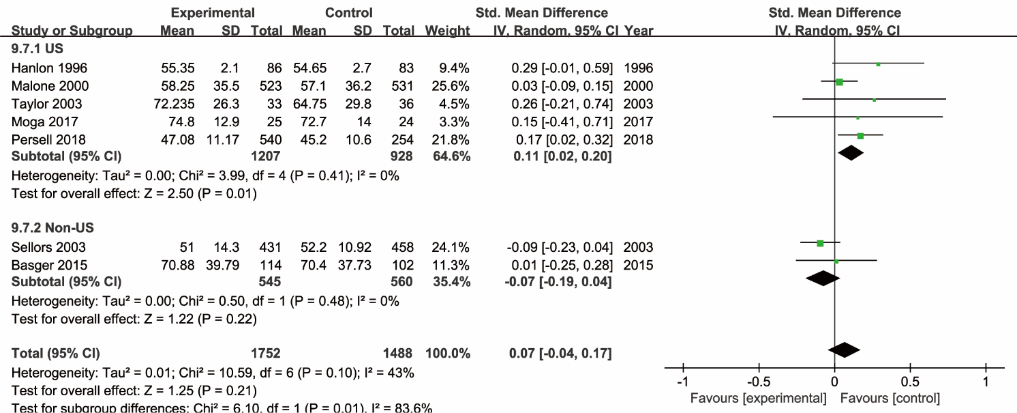


**Supplementary Fig 56.** Subgroup analysis of SF-Mental outcomes: District of studies.


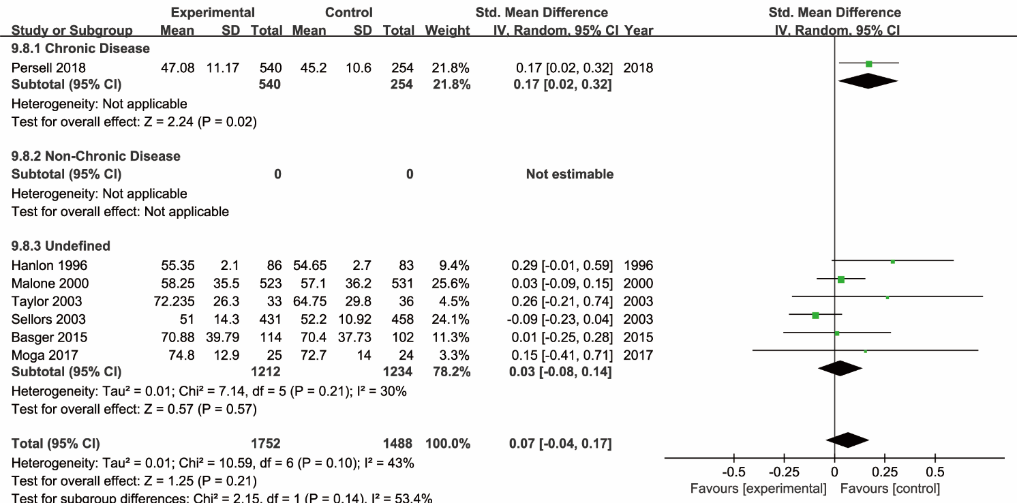


**Supplementary Fig 57.** Subgroup analysis of SF-Mental outcomes: Type of diseases.


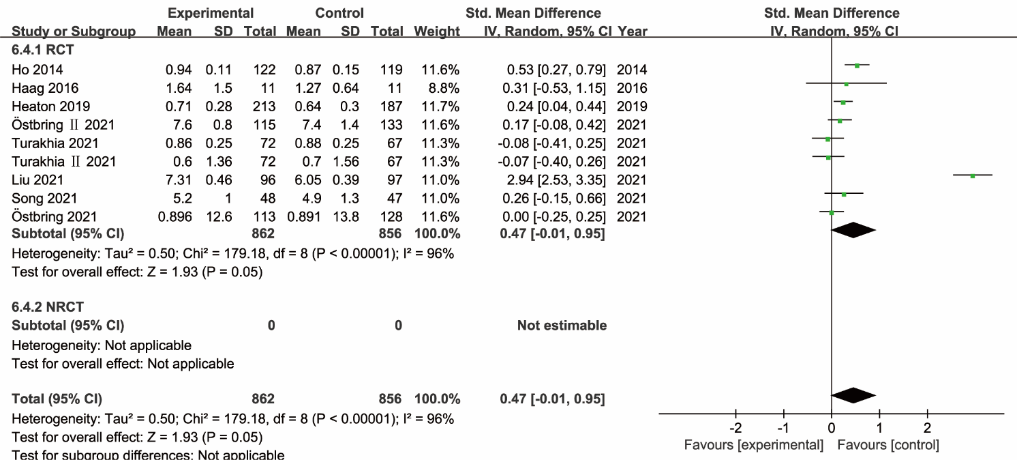


**Supplementary Fig 58.** Subgroup analysis of adherence: Type of studies.


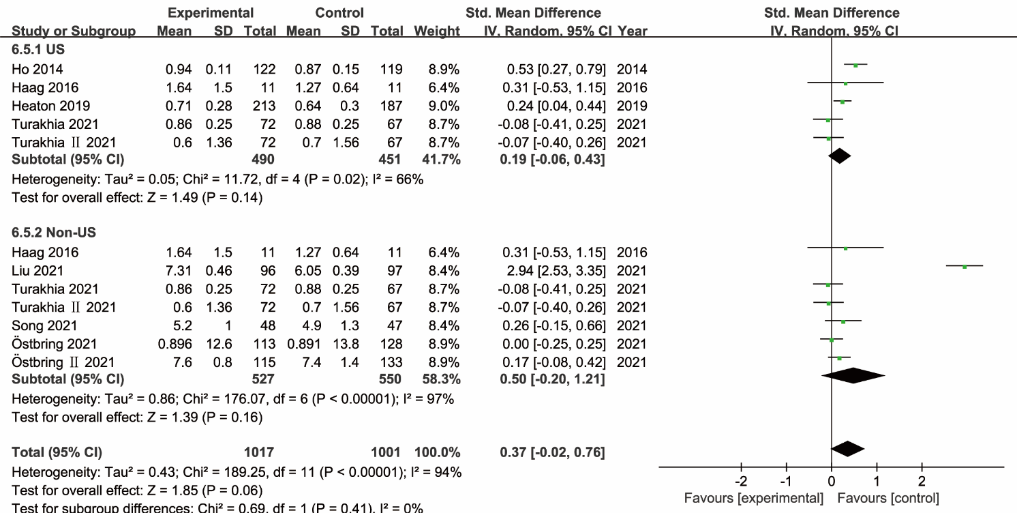


**Supplementary Fig 59.** Subgroup analysis of adherence: District of studies.


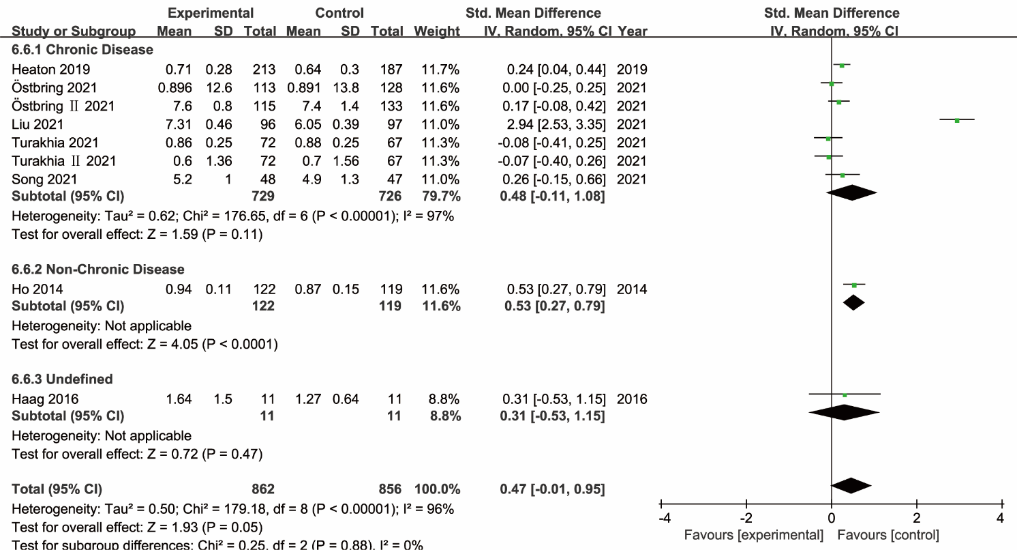


**Supplementary Fig 60.** Subgroup analysis of adherence: Type of diseases.

### Results of publication bias (Funnel plots)

#### Clinical outcomes


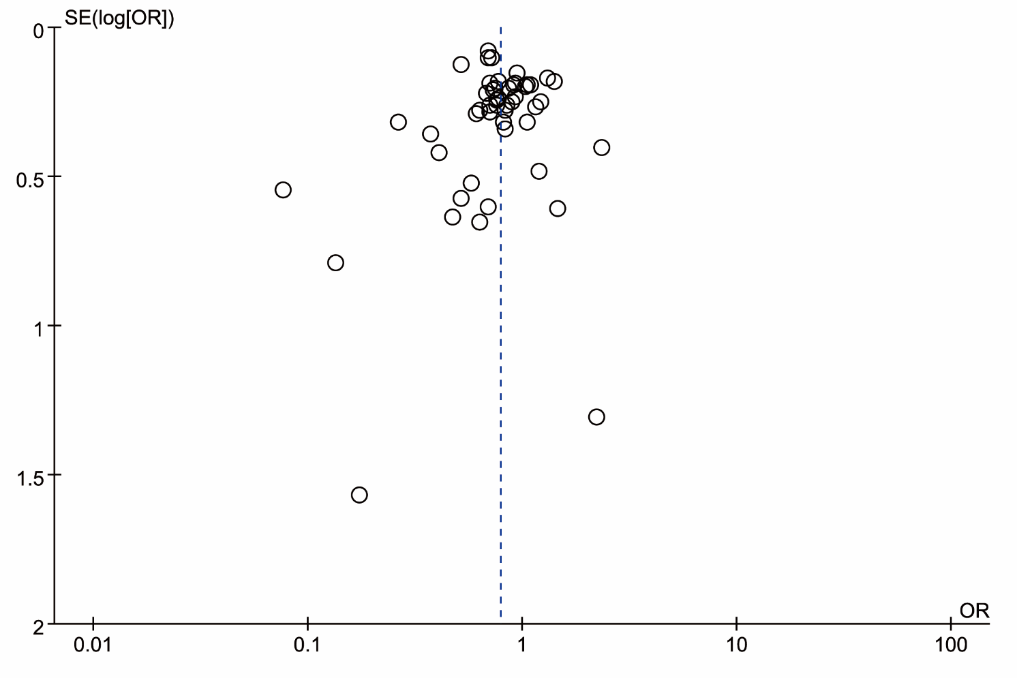


**Supplementary Fig 61.** Funnel plots of readmission.


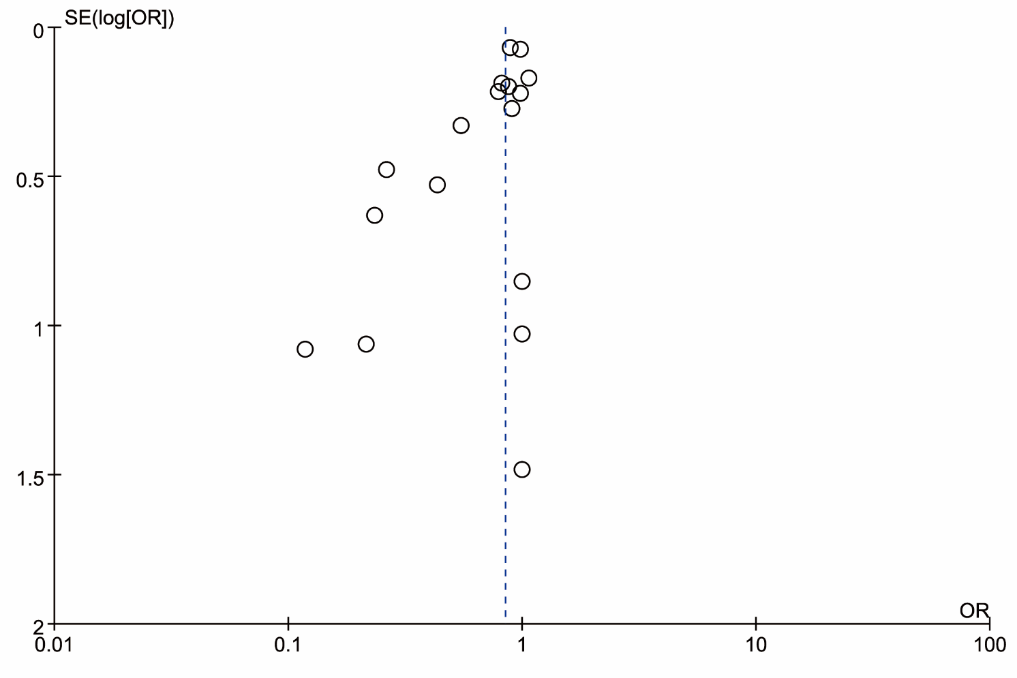


**Supplementary Fig 62.** Funnel plots of ED visit.


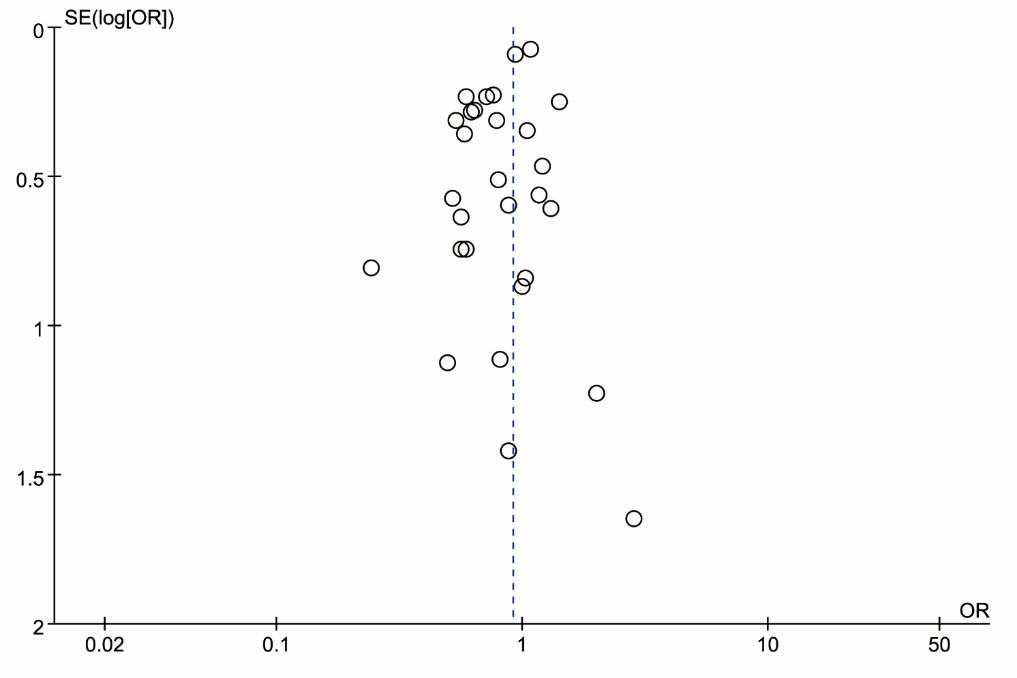


**Supplementary Fig 63.** Funnel plots of mortality.


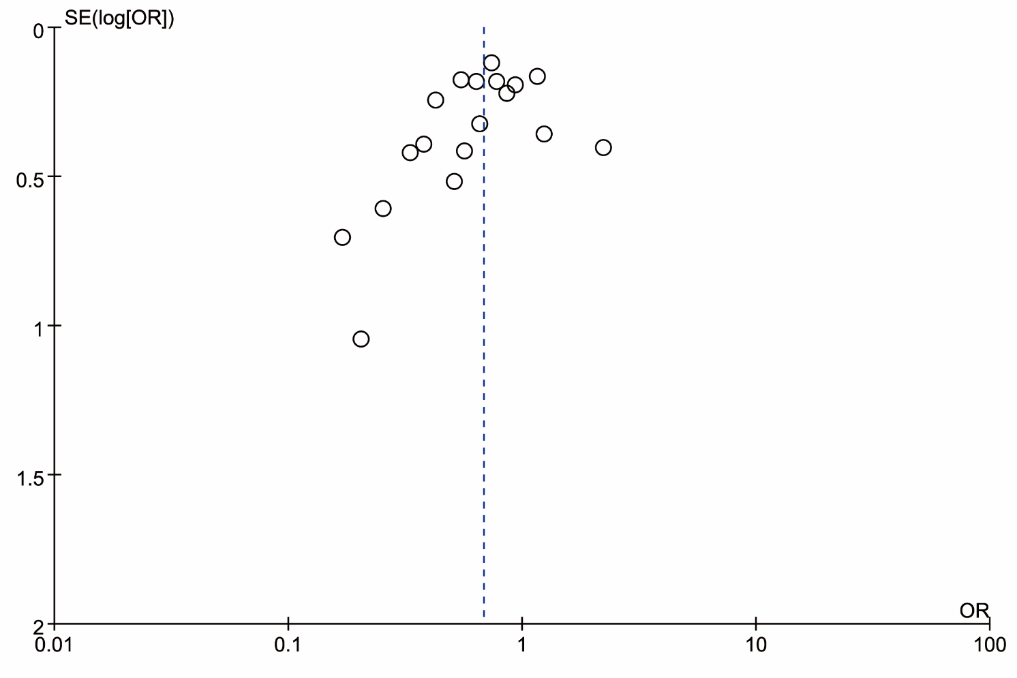


**Supplementary Fig 64.** Funnel plots of all cause ADE.


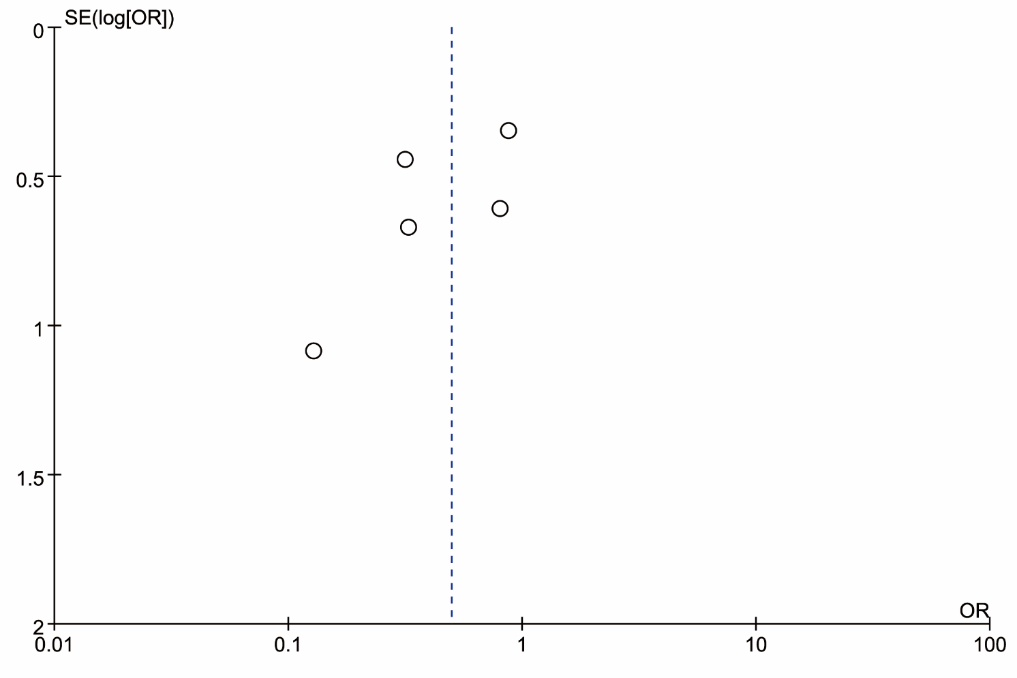


**Supplementary Fig 65.** Funnel plots of SAE.


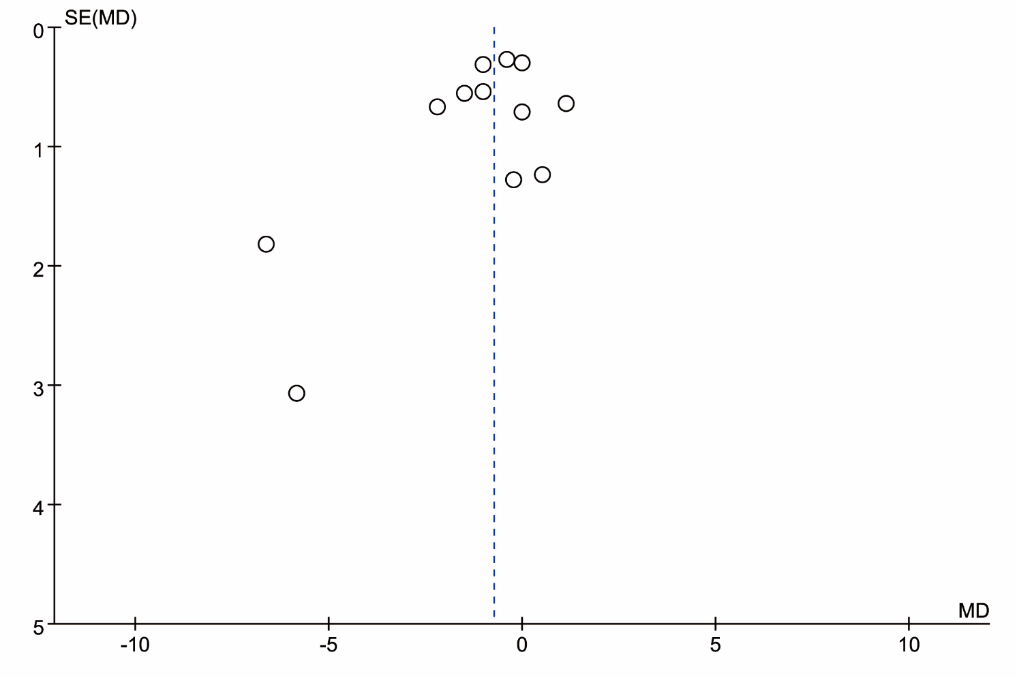


**Supplementary Fig 66.** Funnel plots of LoS.


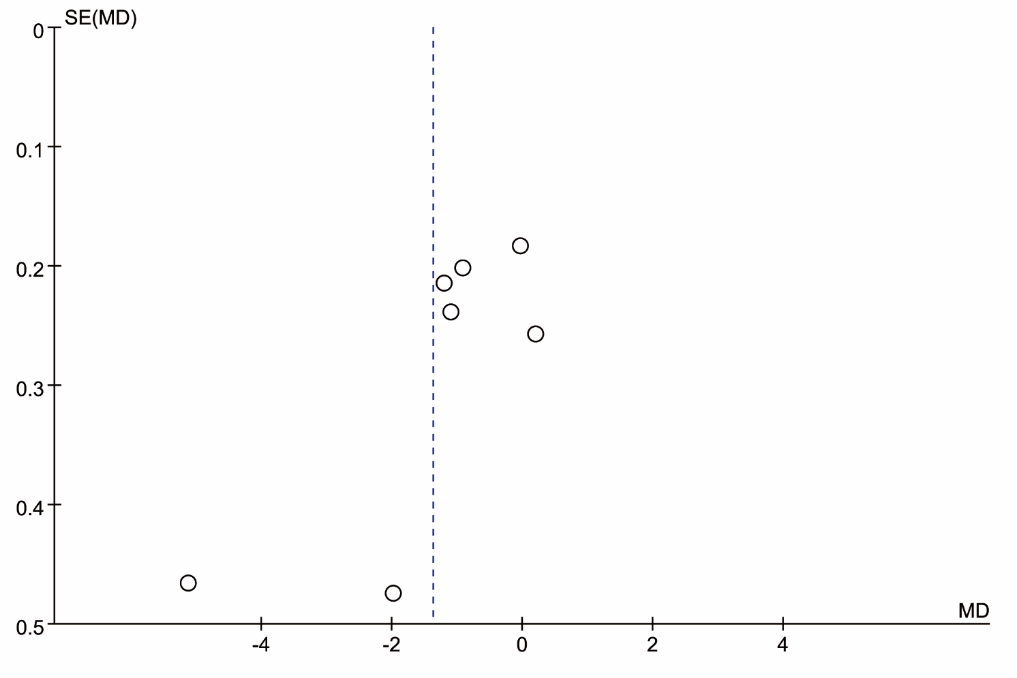


**Supplementary Fig 67.** Funnel plots of DRPs.


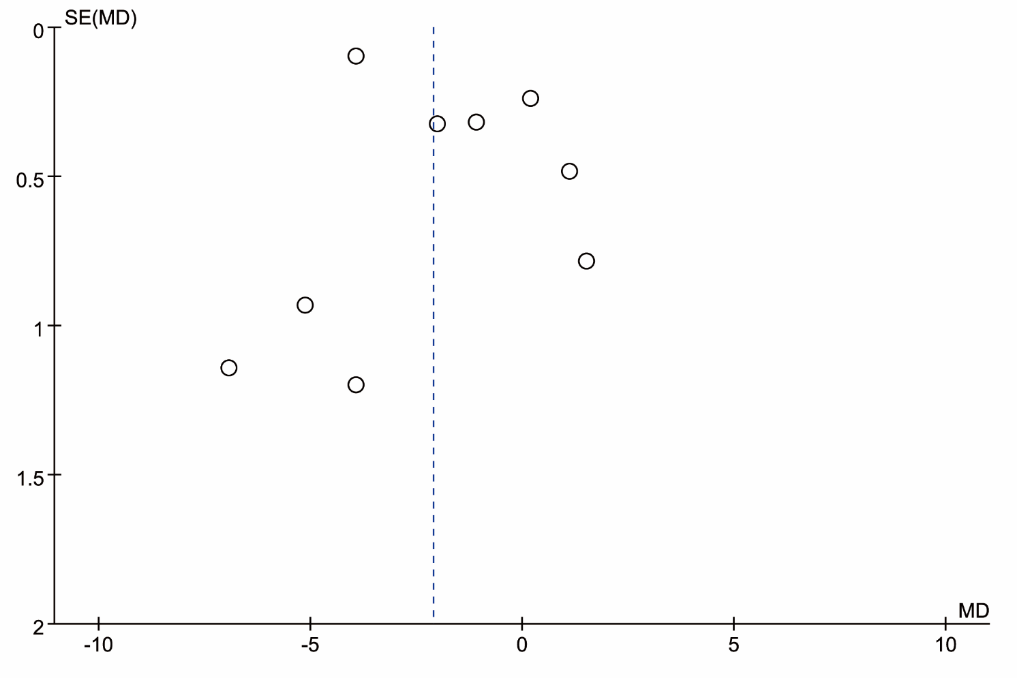


**Supplementary Fig 68.** Funnel plots of MAI.

#### Economic outcomes


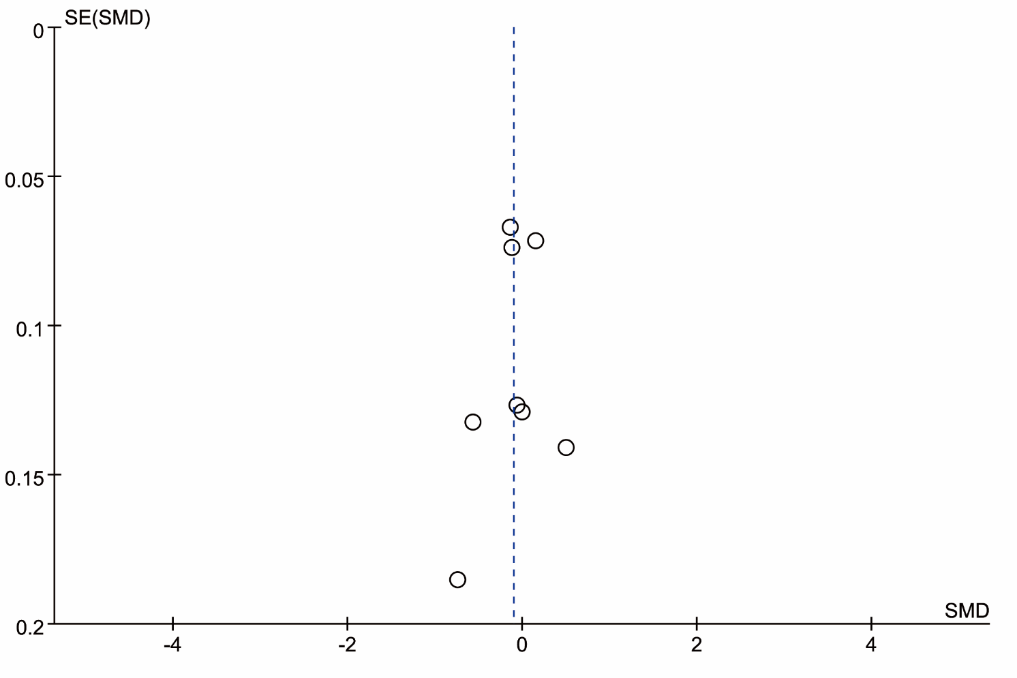


**Supplementary Fig 69.** Funnel plots of total cost.


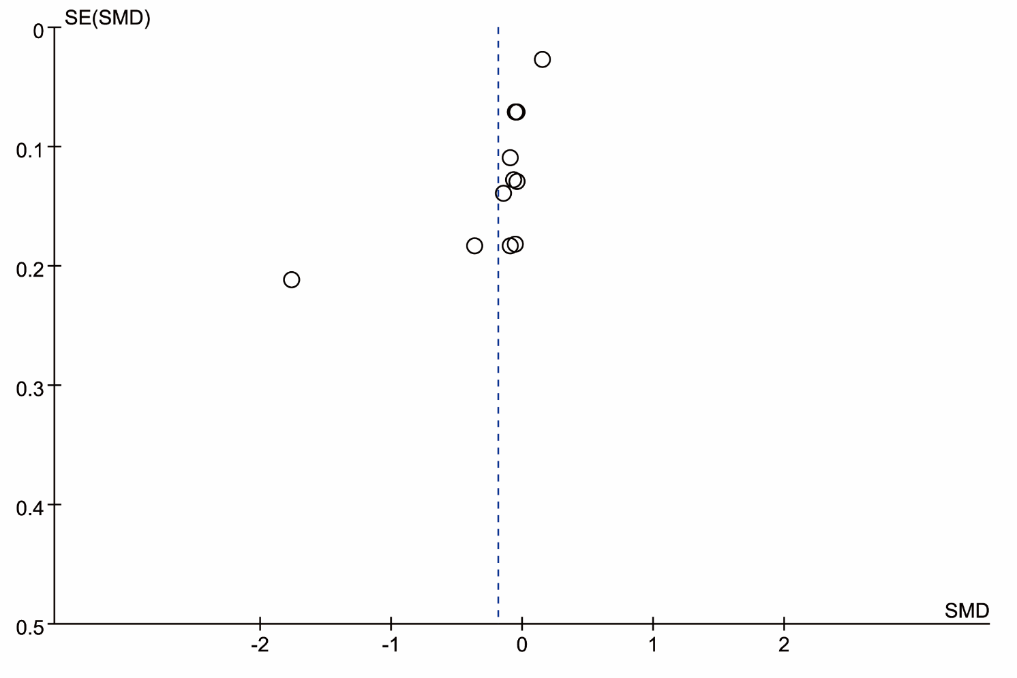


**Supplementary Fig 70.** Funnel plots of medication cost.


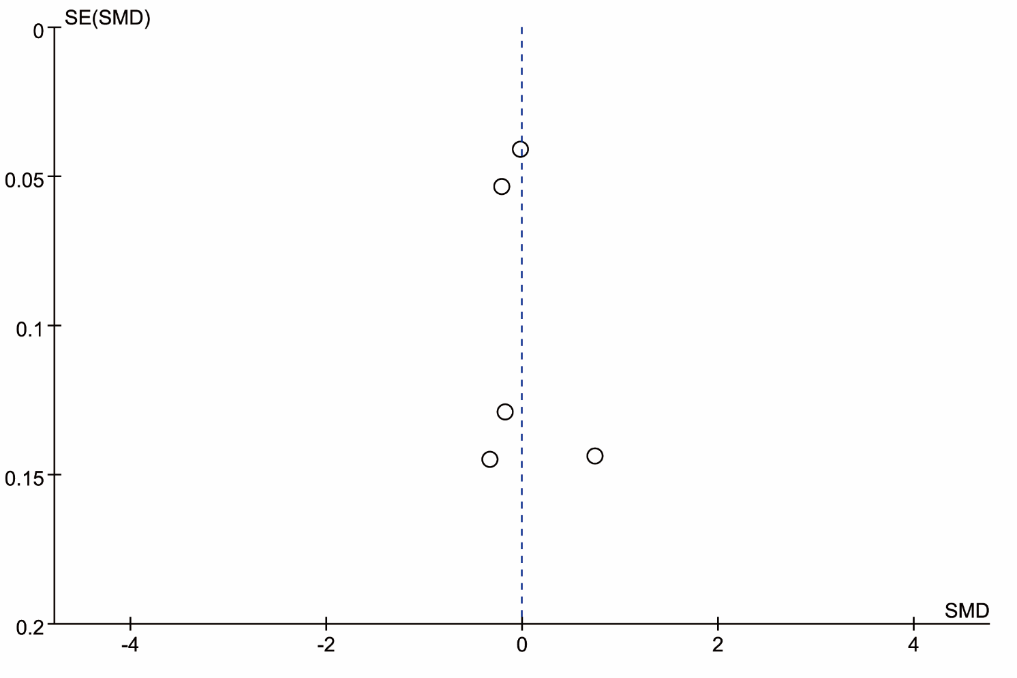


**Supplementary Fig 71.** Funnel plots of cost of hospitalization.

#### Humanistic outcomes


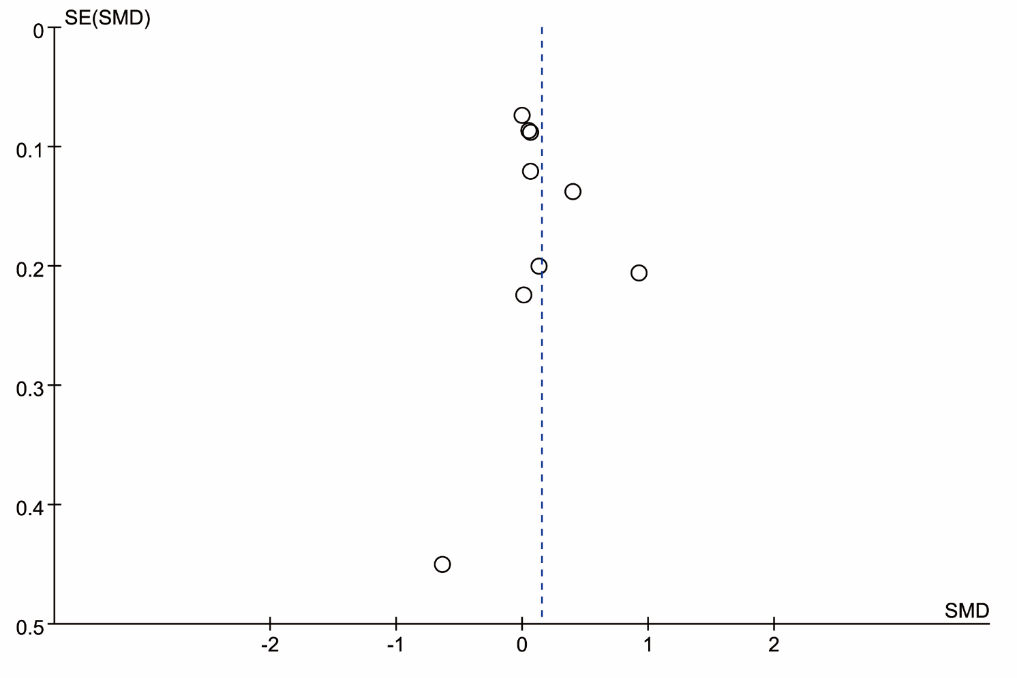


**Supplementary Fig 72.** Funnel plots of EQ-5D.


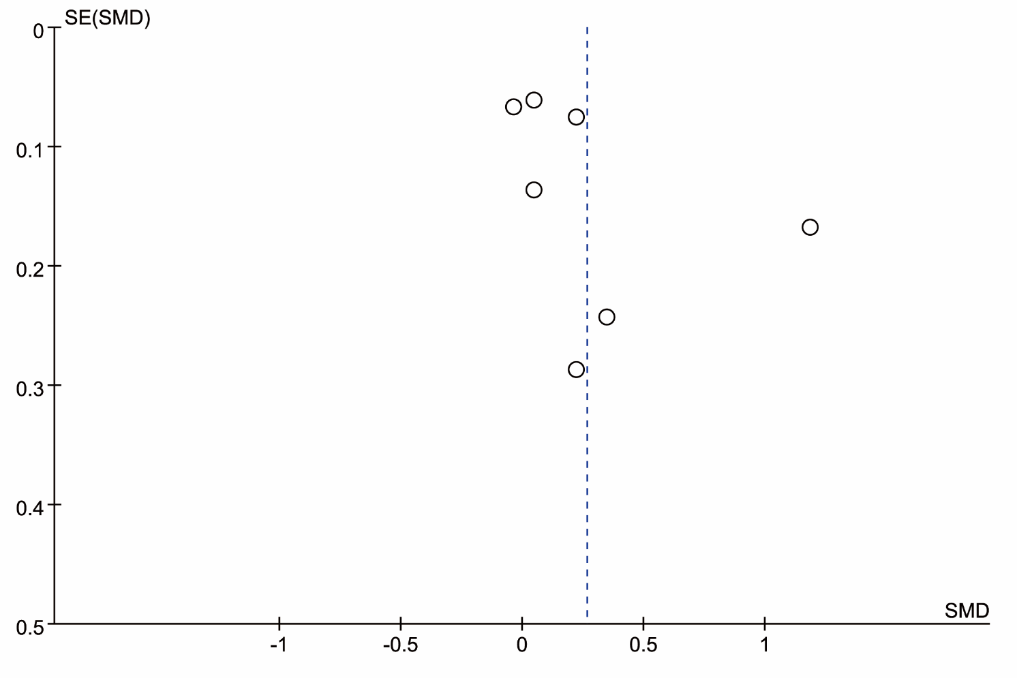


**Supplementary Fig 73.** Funnel plots of SF-Physical outcomes.


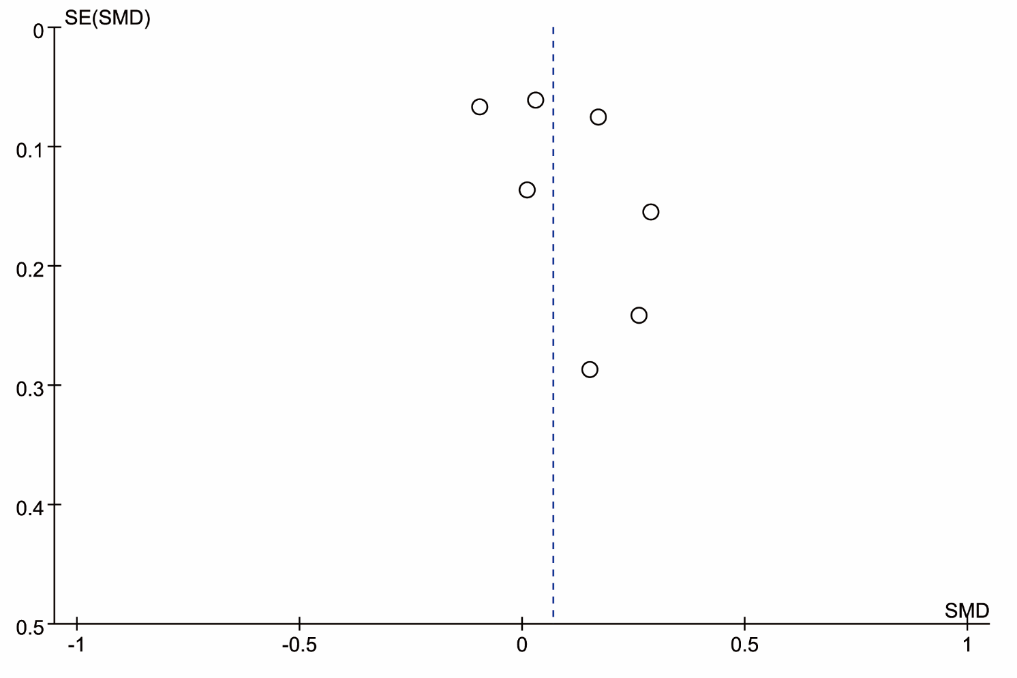


**Supplementary Fig 74.** Funnel plots of SF-Mental outcome.


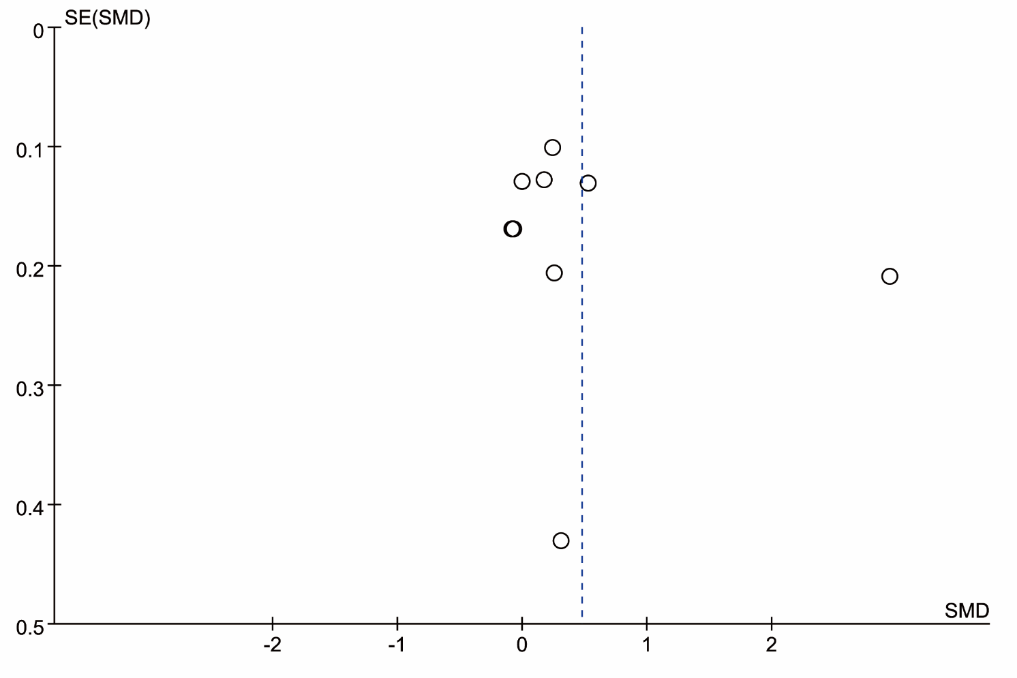


**Supplementary Fig 75.** Funnel plots of adherence.

### Results of sensitivity analysis

#### Clinical outcomes


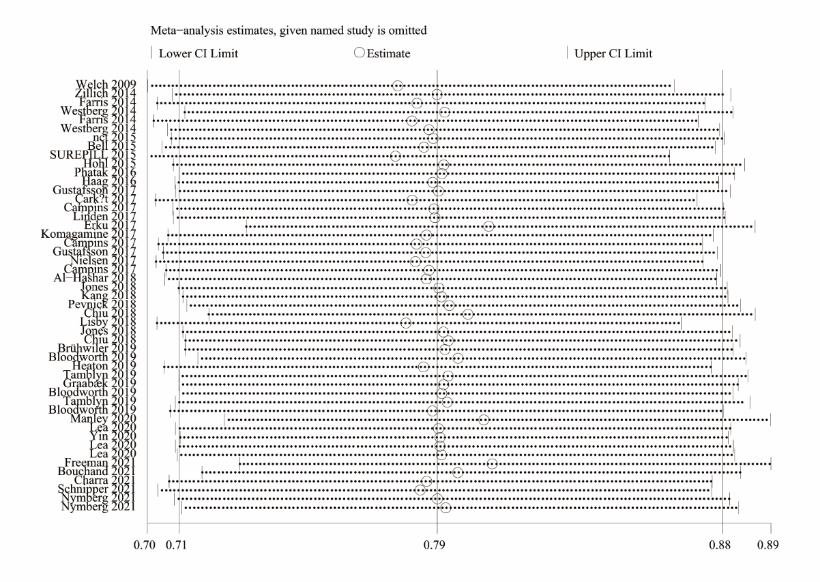


**Supplementary Fig 76.** Result of readmission.


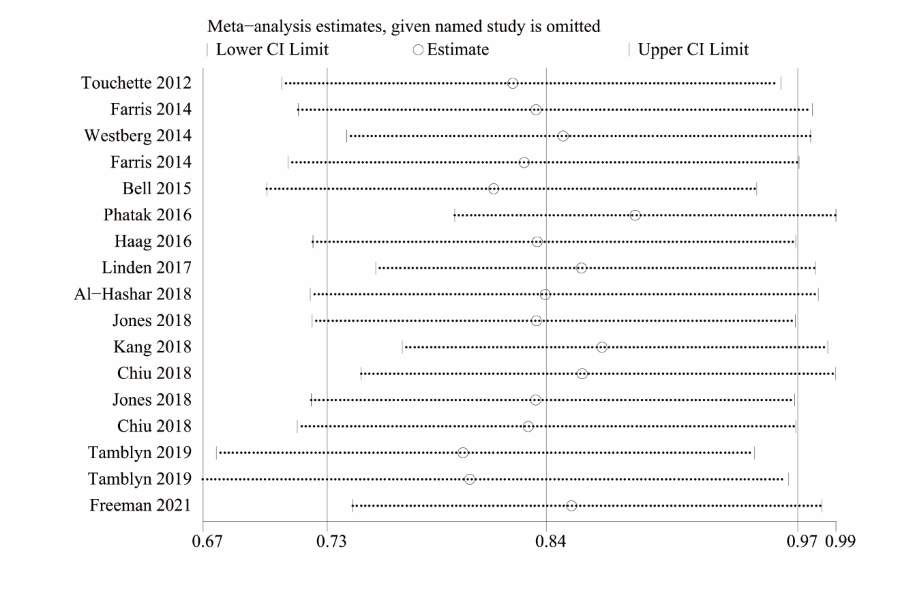


**Supplementary Fig 77.** Result of ED visit.


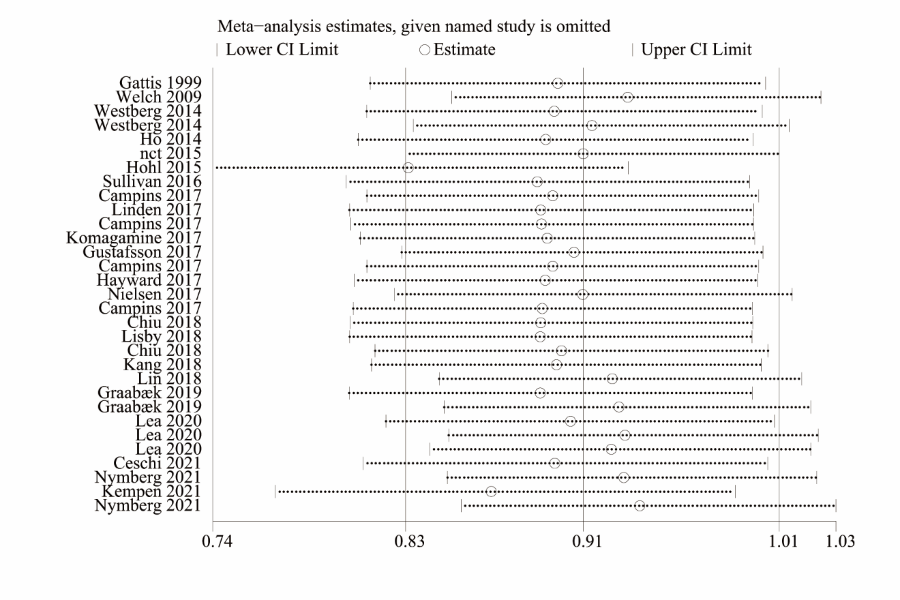


**Supplementary Fig 78.** Result of mortality.


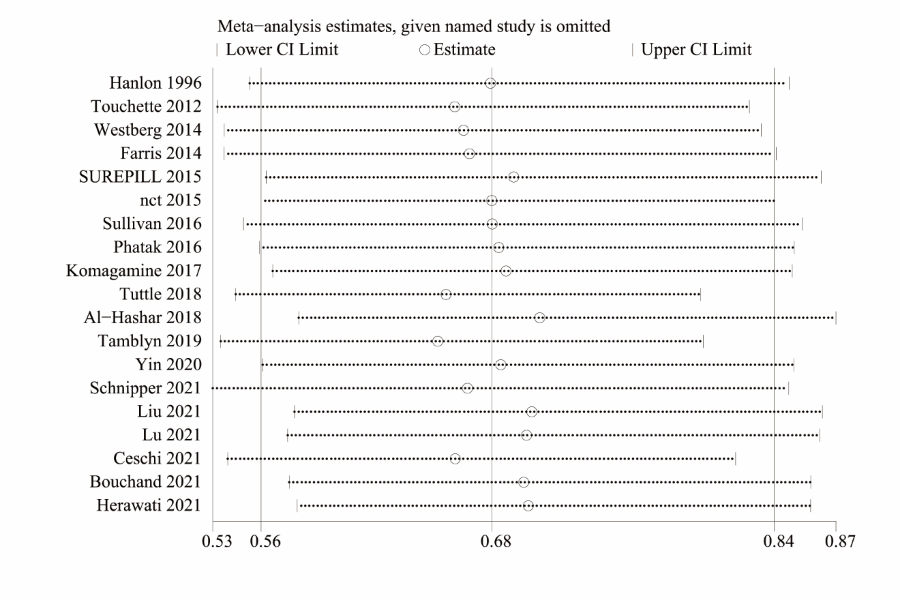


**Supplementary Fig 79.** Result of all cause ADE.


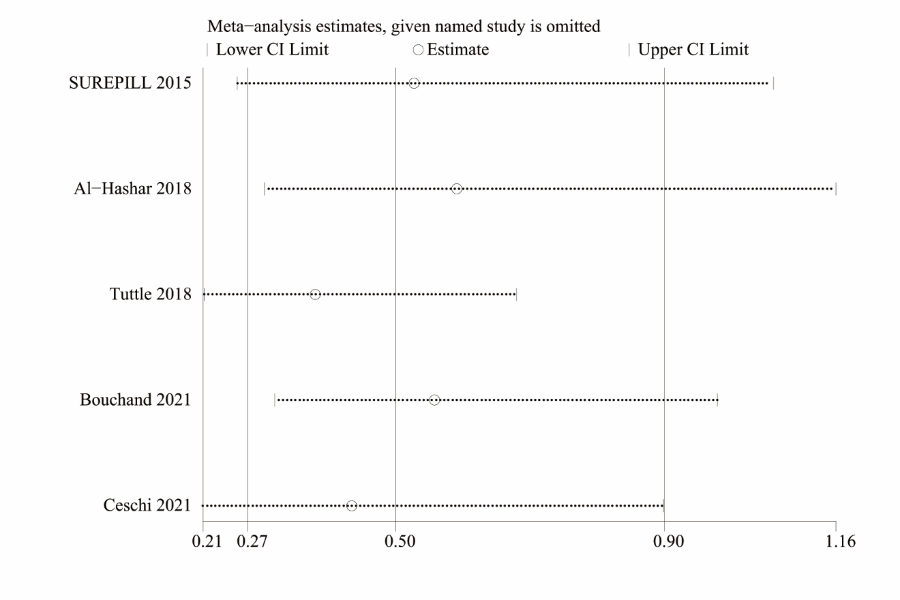


**Supplementary Fig 80.** Result of SAE.


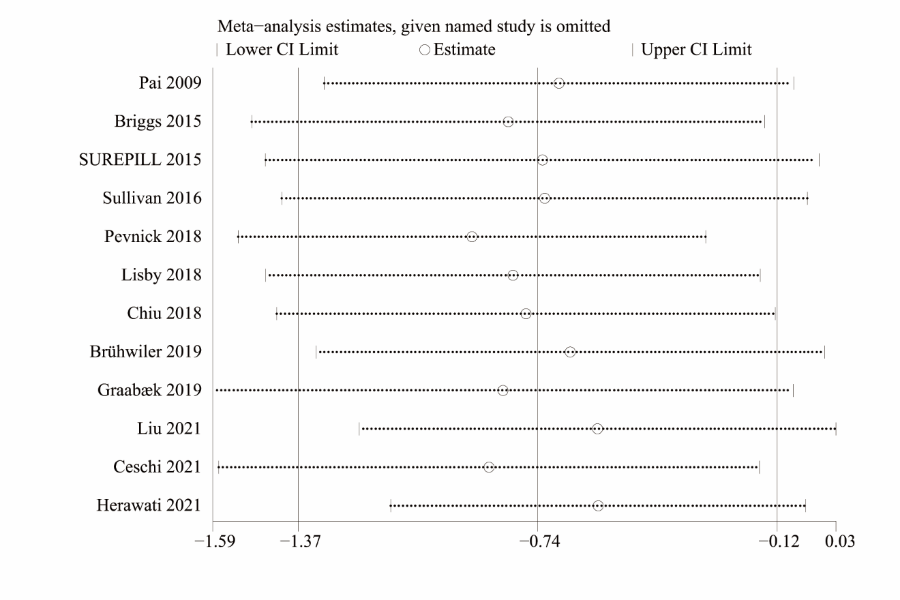


**Supplementary Fig 81.** Result of LoS.


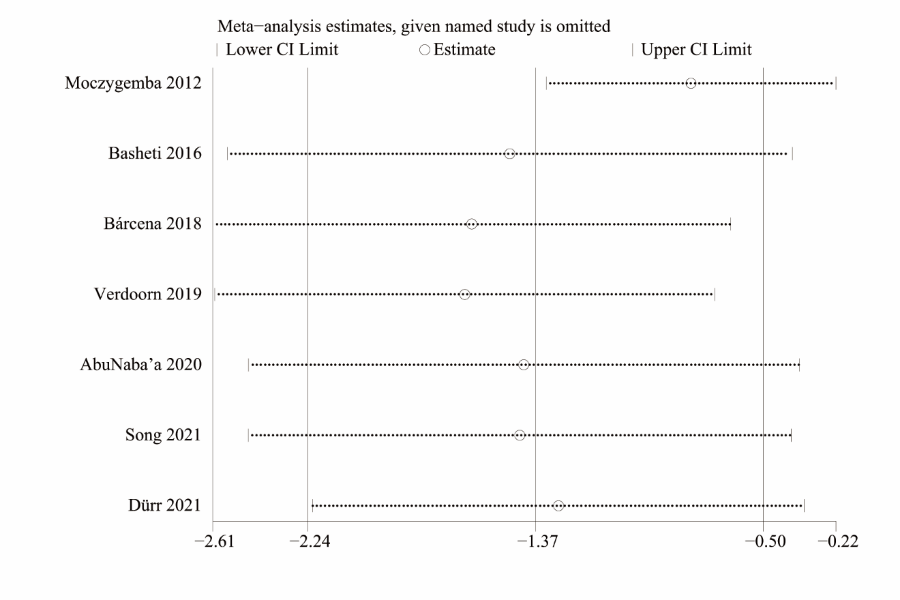


**Supplementary Fig 82.** Result of DRPs.


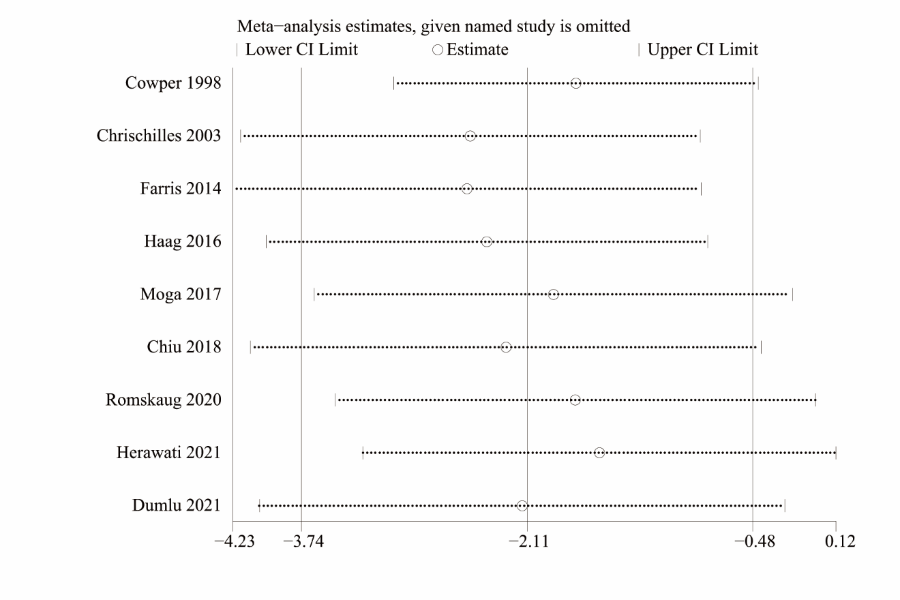


**Supplementary Fig 83.** Result of MAI.

#### Economic outcomes


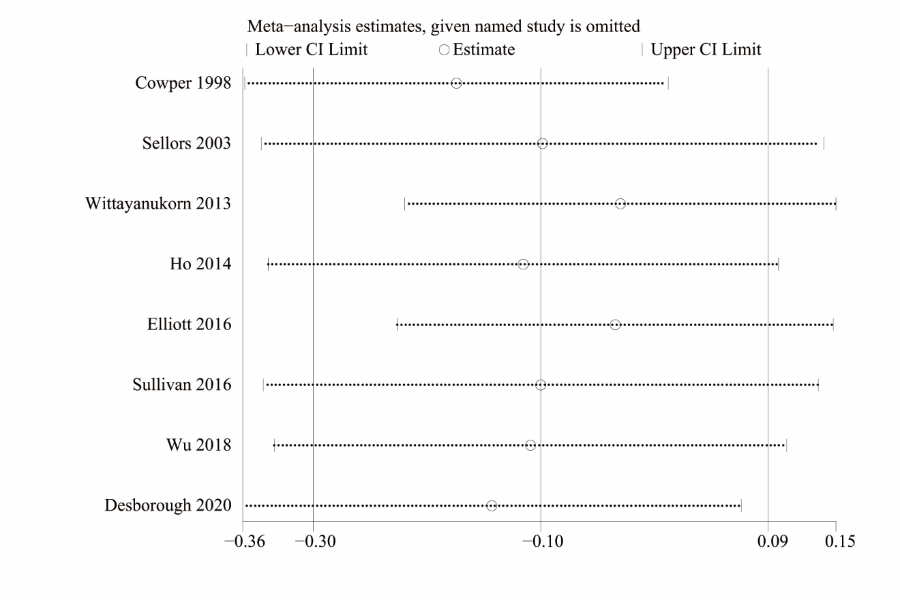


**Supplementary Fig 84.** Result of total cost.


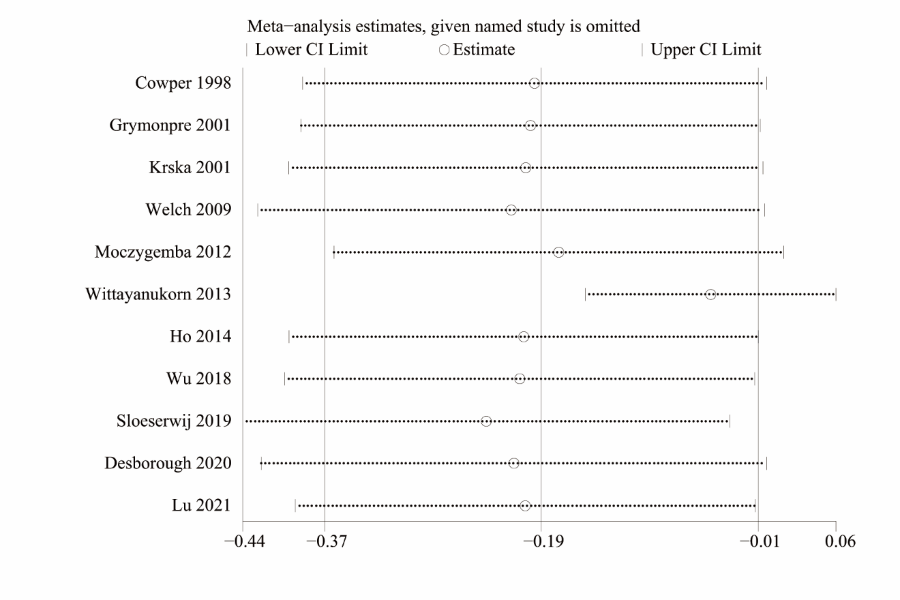


**Supplementary Fig 85.** Result of medication cost.


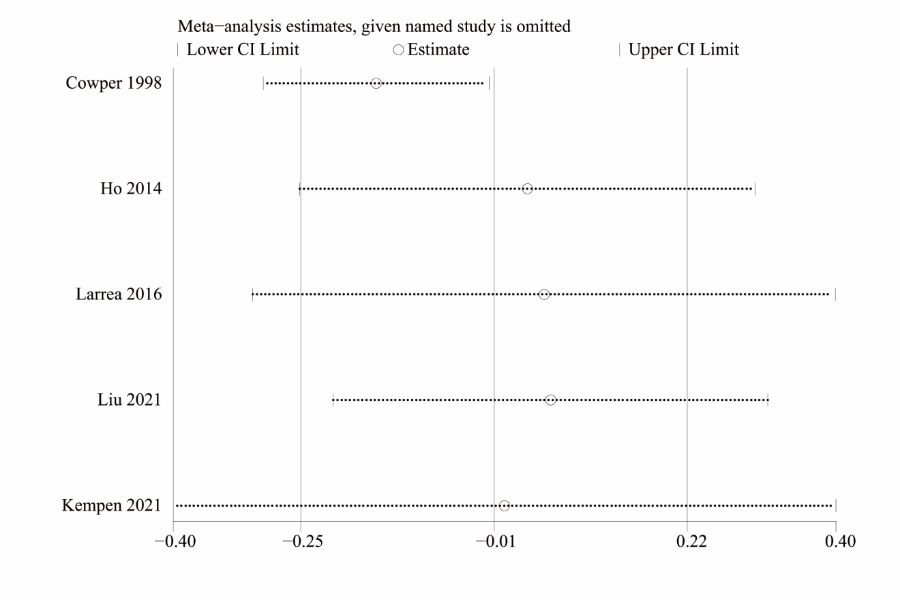


**Supplementary Fig 86.** Result of cost of hospitalization.

#### Humanistic outcomes


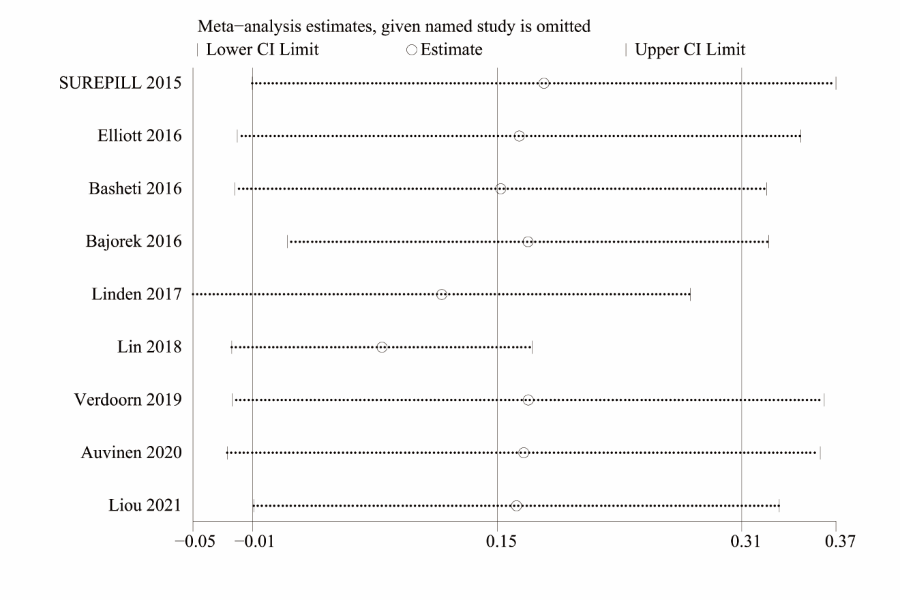


**Supplementary Fig 87.** Result of EQ-5D.


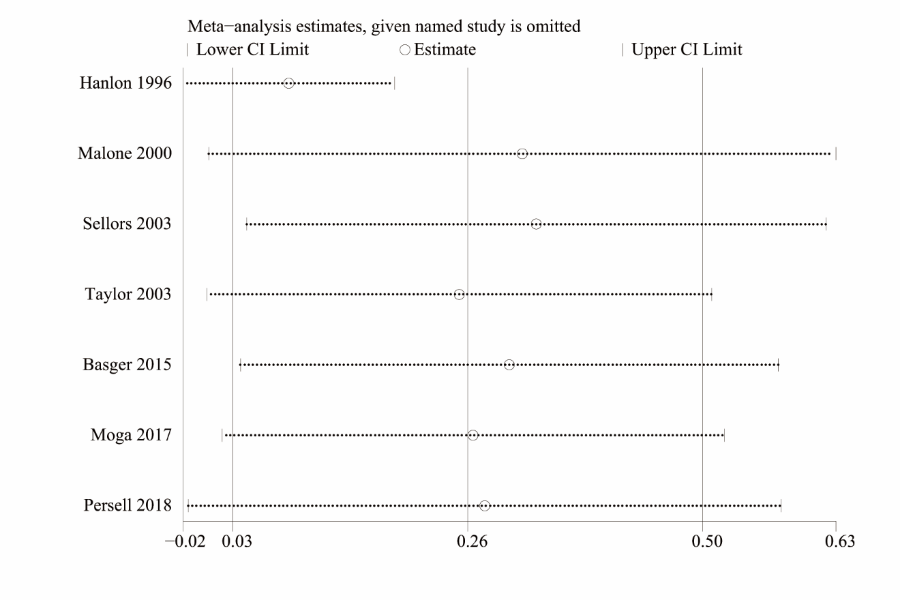


**Supplementary Fig 88.** Result of SF-Physical outcomes.


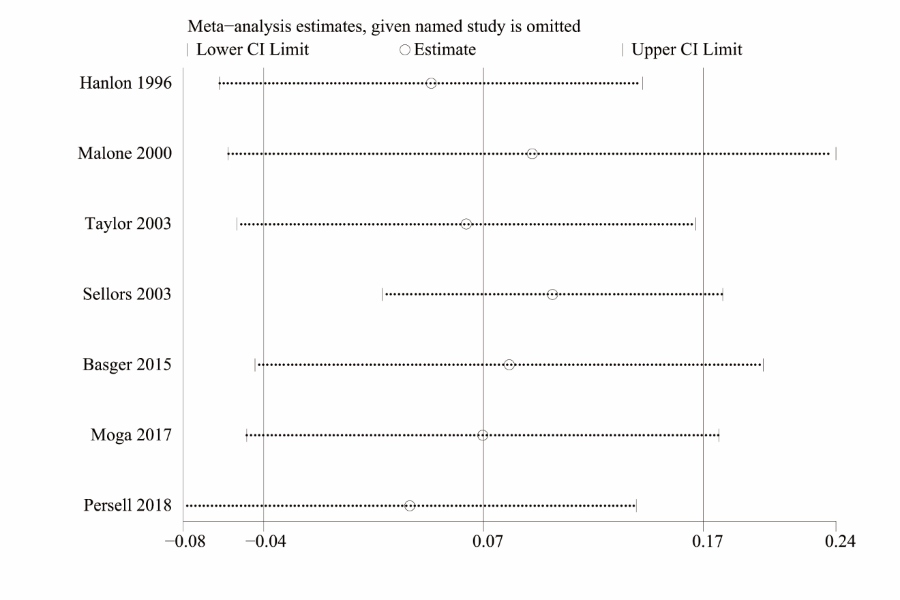


**Supplementary Fig 89.** Result of SF-Mental outcomes.


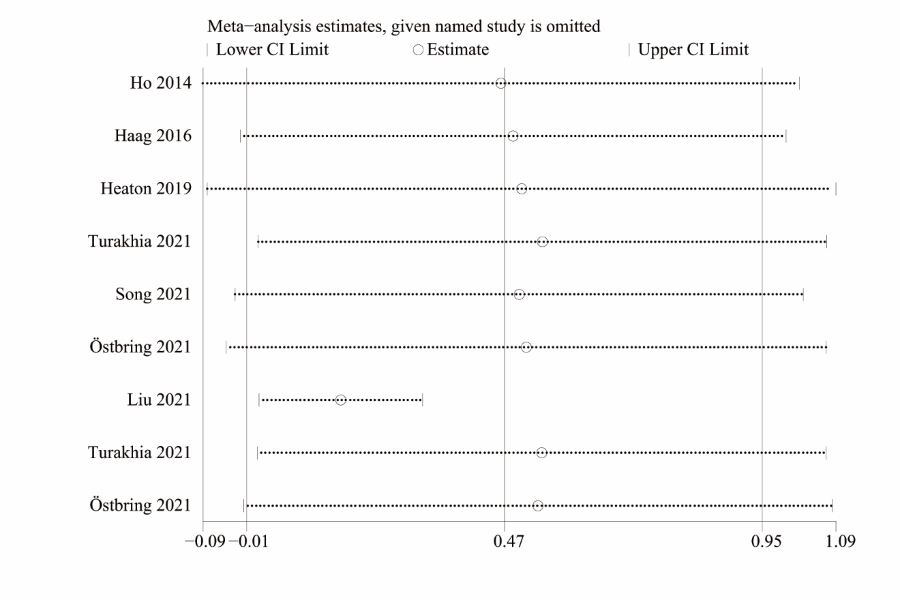


**Supplementary Fig 90.** Result of adherence.

**Reference:**

(2015). Effect of a ward-based pharmacy team on preventable adverse drug events in surgical patients (SUREPILL study). *Br J Surg* 102(10)**,** 1204-1212. doi: 10.1002/bjs.9876.

AbuNaba'a, Y., and Basheti, I.A. (2020). Assessing the impact of medication management review service for females diagnosed with depression and anxiety: a randomized control trial. *Journal of evaluation in clinical practice* 26(5)**,** 1478‐1489. doi: 10.1111/jep.13314.

Al-Hashar, A., Al-Zakwani, I., Eriksson, T., Sarakbi, A., Al-Zadjali, B., Al Mubaihsi, S., et al. (2018). Impact of medication reconciliation and review and counselling, on adverse drug events and healthcare resource use. *International Journal of Clinical Pharmacy* 40(5)**,** 1154-1164. doi: 10.1007/s11096-018-0650-8.

Auvinen, K., Voutilainen, A., Jyrkkä, J., Lönnroos, E., and Mäntyselkä, P. (2020). Interprofessional medication assessment among home care patients: any impact on functioning? Results from a randomised controlled trial. *BMC geriatrics* 20(1)**,** 390. doi: 10.1186/s12877-020-01796-1.

Bajorek, B., Lemay, K.S., Magin, P., Roberts, C., and Armour, C.L. (2016). Implementation and evaluation of a pharmacist-led hypertension management service in primary care: outcomes and methodological challenges. *Pharmacy practice* 14(2) (no pagination). doi: 10.18549/PharmPract.2016.02.723.

Basger, B.J., Moles, R.J., and Chen, T.F. (2015). Impact of an enhanced pharmacy discharge service on prescribing appropriateness criteria: a randomised controlled trial. *International journal of clinical pharmacy* 37(6)**,** 1194‐1205. doi: 10.1007/s11096-015-0186-0.

Basheti, I.A., Al-Qudah, R.A., Obeidat, N.M., and Bulatova, N.R. (2016). Home medication management review in outpatients with chronic diseases in Jordan: a randomized control trial. *International journal of clinical pharmacy* 38(2)**,** 404‐413. doi: 10.1007/s11096-016-0266-9.

Bell, S.P., Schnipper, J.L., Goggins, K., Bian, A., Shintani, A., Roumie, C., et al. (2015). Effect of a pharmacist counseling intervention on healthcare utilization after hospital discharge: a randomized controlled trial. *Journal of general internal medicine* 30**,** S55‐.

Bloodworth, L.S., Malinowski, S.S., Lirette, S.T., and Ross, L.A. (2019). Pharmacist linkage in care transitions: From academic medical center to community. *J Am Pharm Assoc (2003)* 59(6)**,** 896-904. doi: 10.1016/j.japh.2019.08.011.

Bouchand, F., Leplay, C., Guimaraes, R., Fontenay, S., Fellous, L., Dinh, A., et al. (2021). Impact of a medication reconciliation care bundle at hospital discharge on continuity of care: A randomised controlled trial. *Int J Clin Pract* 75(8)**,** e14282. doi: 10.1111/ijcp.14282.

Briggs, S., Pearce, R., Dilworth, S., Higgins, I., Hullick, C., and Attia, J. (2015). Clinical pharmacist review: a randomised controlled trial. *EMA - emergency medicine australasia* 27(5)**,** 419‐426. doi: 10.1111/1742-6723.12451.

Brühwiler, L.D., Beeler, P.E., Böni, F., Giger, R., Wiedemeier, P.G., Hersberger, K.E., et al. (2019). A RCT evaluating a pragmatic in-hospital service to increase the quality of discharge prescriptions. *International journal for quality in health care : journal of the international society for quality in health care* 31(8)**,** G74‐G80. doi: 10.1093/intqhc/mzz043.

Campins, L., Serra-Prat, M., Gózalo, I., López, D., Palomera, E., Agustí, C., et al. (2017). Randomized controlled trial of an intervention to improve drug appropriateness in community-dwelling polymedicated elderly people. *Family practice* 34(1)**,** 36‐42. doi: 10.1093/fampra/cmw073.

Ceschi, A., Noseda, R., Pironi, M., Lazzeri, N., Eberhardt-Gianella, O., Imelli, S., et al. (2021). Effect of Medication Reconciliation at Hospital Admission on 30-Day Returns to Hospital: a Randomized Clinical Trial. *JAMA network open* 4(9)**,** e2124672. doi: 10.1001/jamanetworkopen.2021.24672.

Charra, F., Philippe, M., Herledan, C., Caffin, A.G., Larbre, V., Baudouin, A., et al. (2021). Immunosuppression medication adherence after allogeneic hematopoietic stem cell transplant: Impact of a specialized clinical pharmacy program. *Journal of Oncology Pharmacy Practice*. doi: 10.1177/10781552211000115.

Chiu, P.K.C., Lee, A.W.K., See, T.Y.W., and Chan, F.H.W. (2018). Outcomes of a pharmacist-led medication review programme for hospitalised elderly patients. *Hong kong medical journal* 24(2)**,** 98‐106. doi: 10.12809/hkmj176871.

Chrischilles, E.A., Carter, B.L., Lund, B.C., Rubenstein, L.M., Chen-Hardee, S.S., Voelker, M.D., et al. (2004). Evaluation of the Iowa Medicaid pharmaceutical case management program. *J Am Pharm Assoc (2003)* 44(3)**,** 337-349. doi: 10.1331/154434504323063977.

Cowper, P.A., Weinberger, M., Hanlon, J.T., Landsman, P.B., Samsa, G.P., Uttech, K.M., et al. (1998). The cost-effectiveness of a clinical pharmacist intervention among elderly outpatients. *Pharmacotherapy* 18(2)**,** 327-332.

Desborough, J.A., Clark, A., Houghton, J., Sach, T., Shaw, V., Kirthisingha, V., et al. (2020). Clinical and cost effectiveness of a multi-professional medication reviews in care homes (CAREMED). *International journal of pharmacy practice* 28(6)**,** 626‐634. doi: 10.1111/ijpp.12656.

Dumlu, H., Sancar, M., Ozdemir, A., and Okuyan, B. (2021). Evaluation of clinical pharmacist-led appropriate acid suppression therapy stewardship program in hospitalized older patients: a randomized controlled study. *JACCP journal of the american college of clinical pharmacy* 4(9)**,** 1217‐1218. doi: 10.1002/jac5.1481.

Dürr, P., Schlichtig, K., Kelz, C., Deutsch, B., Maas, R., Eckart, M.J., et al. (2021). The Randomized AMBORA Trial: impact of Pharmacological/Pharmaceutical Care on Medication Safety and Patient-Reported Outcomes During Treatment With New Oral Anticancer Agents. *Journal of clinical oncology* 39(18)**,** 1983‐1994. doi: 10.1200/JCO.20.03088.

Elliott, R.A., Boyd, M.J., Salema, N.E., Davies, J., Barber, N., Mehta, R.L., et al. (2016). Supporting adherence for people starting a new medication for a long-term condition through community pharmacies: A pragmatic randomised controlled trial of the New Medicine Service. *BMJ Quality and Safety* 25(10)**,** 747-758. doi: 10.1136/bmjqs-2015-004400.

Erku, D.A., Belachew, S.A., Tegegn, H.G., and Ayele, A.A. (2017). The impact of pharmacist-led medication therapy management on medication adherence in patients with type 2 diabetes mellitus: a randomized controlled study. *Value in health* 20(9)**,** A402‐. doi: 10.1016/j.jval.2017.08.025.

Farris, K.B., Carter, B.L., Xu, Y., Dawson, J.D., Shelsky, C., Weetman, D.B., et al. (2014). Effect of a care transition intervention by pharmacists: an RCT. *BMC health services research* 14**,** 406. doi: 10.1186/1472-6963-14-406.

Freeman, C.R., Scott, I.A., Hemming, K., Connelly, L.B., Kirkpatrick, C.M., Coombes, I., et al. (2021). Reducing Medical Admissions and Presentations Into Hospital through Optimising Medicines (REMAIN HOME): a stepped wedge, cluster randomised controlled trial. *Med J Aust* 214(5)**,** 212-217. doi: 10.5694/mja2.50942.

Gattis, W.A., Hasselblad, V., Whellan, D.J., and O'Connor, C.M. (1999). Reduction in heart failure events by the addition of a clinical pharmacist to the heart failure management team: results of the Pharmacist in Heart Failure Assessment Recommendation and Monitoring (PHARM) Study. *Arch Intern Med* 159(16)**,** 1939-1945. doi: 10.1001/archinte.159.16.1939.

Graabaek, T., Hedegaard, U., Christensen, M.B., Clemmensen, M.H., Knudsen, T., and Aagaard, L. (2019). Effect of a medicines management model on medication-related readmissions in older patients admitted to a medical acute admission unit-A randomized controlled trial. *Journal of evaluation in clinical practice* 25(1)**,** 88‐96. doi: 10.1111/jep.13013.

Grymonpre, R.E., Williamson, D.A., and Montgomery, P.R. (2011). Impact of a pharmaceutical care model for non-institutionalised elderly: results of a randomised, controlled trial. *International Journal of Pharmacy Practice* 9(4)**,** 235-241. doi: 10.1111/j.2042-7174.2001.tb01054.x.

Gustafsson, M., Sjölander, M., Pfister, B., Jonsson, J., Schneede, J., and Lövheim, H. (2017). Pharmacist participation in hospital ward teams and hospital readmission rates among people with dementia: a randomized controlled trial. *European journal of clinical pharmacology* 73(7)**,** 827‐835. doi: 10.1007/s00228-017-2249-8.

Haag, J.D., Davis, A.Z., Hoel, R.W., Armon, J.J., Odell, L.J., Dierkhising, R.A., et al. (2016). Impact of pharmacist-provided medication therapy management on healthcare quality and utilization in recently discharged elderly patients. *American health and drug benefits* 9(5)**,** 259‐267.

Hanlon, J.T., Weinberger, M., Samsa, G.P., Schmader, K.E., Uttech, K.M., Lewis, I.K., et al. (1996). A randomized, controlled trial of a clinical pharmacist intervention to improve inappropriate prescribing in elderly outpatients with polypharmacy. *Am J Med* 100(4)**,** 428-437. doi: 10.1016/s0002-9343(97)89519-8.

Hayward, K.L., Patel, P.J., Valery, P.C., Horsfall, L.U., Li, C.Y., Wright, P.L., et al. (2017). High-risk medication-related problems are prevalent in people with decompensated cirrhosis. *Journal of gastroenterology and hepatology (australia)* 32**,** 95‐. doi: 10.1111/jgh.13893.

Heaton, P.C., Frede, S., Kordahi, A., Lowery, L., Moorhead, B., Kirby, J., et al. (2019). Improving care transitions through medication therapy management: a community partnership to reduce readmissions in multiple health-systems. *Journal of the American Pharmacists Association : JAPhA* 59(3)**,** 319‐328. doi: 10.1016/j.japh.2019.01.005.

Herawati, F., Maharjana, I.B.N., Kuswardhani, T., and Susilo, A.P. (2021). STOPP-START Medication Review: A Non-Randomized Trial in an Indonesian Tertiary Hospital to Improve Medication Appropriateness and to Reduce the Length of Stay of Older Adults. *Hospital Pharmacy* 56(6)**,** 668-677. doi: 10.1177/0018578720942227.

Ho, P.M., Lambert-Kerzner, A., Carey, E.P., Fahdi, I.E., Bryson, C.L., Melnyk, S.D., et al. (2014). Multifaceted intervention to improve medication adherence and secondary prevention measures after acute coronary syndrome hospital discharge: a randomized clinical trial. *JAMA internal medicine* 174(2)**,** 186‐193. doi: 10.1001/jamainternmed.2013.12944.

Hohl, C.M., Wickham, M.E., Partovi, N., Ghement, I., McGrail, K., and Sobolev, B.G. (2015). Effect of pharmacist-led medication review in the emergency department on downstream health services utilization. *Academic emergency medicine* 22(5 SUPPL. 1)**,** S281. doi: 10.1111/acem.12644.

Jones, C.D., Anthony, A., Klein, M.D., Shakowski, C., Smith, H.K., Go, A., et al. (2018). The effect of a pharmacist-led multidisciplinary transitions-of-care pilot for patients at high risk of readmission. *Journal of the American Pharmacists Association : JAPhA* 58(5)**,** 554‐560. doi: 10.1016/j.japh.2018.05.008.

Kang, J.E., Yu, J.M., Choi, J.H., Chung, I.M., Pyun, W.B., Kim, S.A., et al. (2018). Development and clinical application of an evidence-based pharmaceutical care service algorithm in acute coronary syndrome. *J Clin Pharm Ther* 43(3)**,** 366-376. doi: 10.1111/jcpt.12665.

Karapinar-Çarkıt, F., van der Knaap, R., Bouhannouch, F., Borgsteede, S.D., Janssen, M.J.A., Siegert, C.E.H., et al. (2017). Cost-effectiveness of a transitional pharmaceutical care program for patients discharged from the hospital. *PloS one* 12(4)**,** e0174513. doi: 10.1371/journal.pone.0174513.

Kempen, T.G.H., Bertilsson, M., Hadziosmanovic, N., Lindner, K.J., Melhus, H., Nielsen, E.I., et al. (2021). Effects of Hospital-Based Comprehensive Medication Reviews Including Postdischarge Follow-up on Older Patients' Use of Health Care: a Cluster Randomized Clinical Trial. *JAMA network open* 4(4)**,** e216303. doi: 10.1001/jamanetworkopen.2021.6303.

Komagamine, J., and Hagane, K. (2017). Intervention to improve the appropriate use of polypharmacy for older patients with hip fractures: an observational study. *BMC Geriatr* 17(1)**,** 288. doi: 10.1186/s12877-017-0681-3.

Krska, J., Cromarty, J.A., Arris, F., Jamieson, D., Hansford, D., Duffus, P.R., et al. (2001). Pharmacist-led medication review in patients over 65: a randomized, controlled trial in primary care. *Age Ageing* 30(3)**,** 205-211. doi: 10.1093/ageing/30.3.205.

Lea, M., Mowé, M., Molden, E., Kvernrød, K., Skovlund, E., and Mathiesen, L. (2020). Effect of medicines management versus standard care on readmissions in multimorbid patients: a randomised controlled trial. *BMJ open* 10(12)**,** e041558. doi: 10.1136/bmjopen-2020-041558.

Lin, H.W., Lin, C.H., Chang, C.K., Chou, C.Y., Yu, I.W., Lin, C.C., et al. (2018). Economic outcomes of pharmacist-physician medication therapy management for polypharmacy elderly: a prospective, randomized, controlled trial. *Journal of the Formosan Medical Association / Taiwan yi zhi* 117(3)**,** 235‐243. doi: 10.1016/j.jfma.2017.04.017.

Liou, W.S., Huang, S.M., Lee, W.H., Chang, Y.L., and Wu, M.F. (2021). The effects of a pharmacist-led medication review in a nursing home: A randomized controlled trial. *Medicine* 100(48)**,** e28023. doi: 10.1097/MD.0000000000028023.

Lisby, M., Bonnerup, D.K., Brock, B., Gregersen, P.A., Jensen, J., Larsen, M.L., et al. (2018). Medication Review and Patient Outcomes in an Orthopedic Department: a Randomized Controlled Study. *Journal of patient safety* 14(2)**,** 74‐81. doi: 10.1097/PTS.0000000000000173.

Liu, M., Liu, J., Geng, Z., and Bai, S. (2021). Evaluation of outcomes of medication therapy management (Mtm) services for patients with chronic obstructive pulmonary disease (copd). *Pakistan Journal of Medical Sciences* 37(7)**,** 1832‐1836. doi: 10.12669/pjms.37.7.4518.

Lu, Z., Li, Y., He, Y., Zhai, Y., Wu, J., Wang, J., et al. (2021). Internet-Based Medication Management Services Improve Glycated Hemoglobin Levels in Patients with Type 2 Diabetes. *Telemedicine journal and e-health* 27(6)**,** 686‐693. doi: 10.1089/tmj.2020.0123.

Malet-Larrea, A., Goyenechea, E., García-Cárdenas, V., Calvo, B., Arteche, J.M., Aranegui, P., et al. (2016). The impact of a medication review with follow-up service on hospital admissions in aged polypharmacy patients. *Br J Clin Pharmacol* 82(3)**,** 831-838. doi: 10.1111/bcp.13012.

Malone, D.C., Carter, B.L., Billups, S.J., Valuck, R.J., Barnette, D.J., Sintek, C.D., et al. (2000). An economic analysis of a randomized, controlled, multicenter study of clinical pharmacist interventions for high-risk veterans: the IMPROVE study. Impact of Managed Pharmaceutical Care Resource Utilization and Outcomes in Veterans Affairs Medical Centers. *Pharmacotherapy* 20(10)**,** 1149-1158. doi: 10.1592/phco.20.15.1149.34590.

Manley, H.J., Aweh, G., Weiner, D.E., Jiang, H., Miskulin, D.C., Johnson, D., et al. (2020). Multidisciplinary Medication Therapy Management and Hospital Readmission in Patients Undergoing Maintenance Dialysis: A Retrospective Cohort Study. *Am J Kidney Dis* 76(1)**,** 13-21. doi: 10.1053/j.ajkd.2019.12.002.

Moczygemba, L.R., Barner, J.C., and Gabrillo, E.R. (2012). Outcomes of a Medicare Part D telephone medication therapy management program. *J Am Pharm Assoc (2003)* 52(6)**,** e144-152. doi: 10.1331/JAPhA.2012.11258.

Moga, D.C., Abner, E.L., Rigsby, D.N., Eckmann, L., Huffmyer, M., Murphy, R.R., et al. (2017). Optimizing medication appropriateness in older adults: a randomized clinical interventional trial to decrease anticholinergic burden. *Alzheimer's research & therapy* 9(1)**,** 36. doi: 10.1186/s13195-017-0263-9.

Nielsen, T.R.H., Honoré, P.H., Rasmussen, M., and Andersen, S.E. (2017). Clinical Effects of a Pharmacist Intervention in Acute Wards – A Randomized Controlled Trial. *Basic and Clinical Pharmacology and Toxicology* 121(4)**,** 325-333. doi: 10.1111/bcpt.12802.

Nymberg, V.M., Lenander, C., and Bolmsjo, B.B. (2021). The impact of medication reviews conducted in primary care on hospital admissions and mortality: An observational follow-up of a randomized controlled trial. *Drug, Healthcare and Patient Safety* 13**,** 1‐9. doi: 10.2147/DHPS.S283708.

O'Sullivan, D., O'Mahony, D., O'Connor, M.N., Gallagher, P., Gallagher, J., Cullinan, S., et al. (2016). Prevention of Adverse Drug Reactions in Hospitalised Older Patients Using a Software-Supported Structured Pharmacist Intervention: A Cluster Randomised Controlled Trial. *Drugs Aging* 33(1)**,** 63-73. doi: 10.1007/s40266-015-0329-y.

Östbring, M.J., Eriksson, T., Petersson, G., and Hellström, L. (2021). Effects of a pharmaceutical care intervention on clinical outcomes and patient adherence in coronary heart disease: the MIMeRiC randomized controlled trial. *BMC cardiovascular disorders* 21(1)**,** 367. doi: 10.1186/s12872-021-02178-0.

Pai, A.B., Boyd, A., Chavez, A., and Manley, H.J. (2009). Health-related quality of life is maintained in hemodialysis patients receiving pharmaceutical care: a 2-year randomized, controlled study. *Hemodial Int* 13(1)**,** 72-79. doi: 10.1111/j.1542-4758.2009.00328.x.

Persell, S.D., Karmali, K.N., Lazar, D., Friesema, E.M., Lee, J.Y., Rademaker, A., et al. (2018). Effect of electronic health record–based medication support and nurse-led medication therapy management on hypertension and medication self-management: A randomized clinical trial. *JAMA Internal Medicine* 178(8)**,** 1069-1077. doi: 10.1001/jamainternmed.2018.2372.

Pevnick, J.M., Nguyen, C., Jackevicius, C.A., Palmer, K.A., Shane, R., Cook-Wiens, G., et al. (2018). Improving admission medication reconciliation with pharmacists or pharmacy technicians in the emergency department: a randomised controlled trial. *BMJ quality & safety* 27(7)**,** 512‐520. doi: 10.1136/bmjqs-2017-006761.

Phatak, A., Prusi, R., Ward, B., Hansen, L.O., Williams, M.V., Vetter, E., et al. (2016). Impact of pharmacist involvement in the transitional care of high-risk patients through medication reconciliation, medication education, and postdischarge call-backs (IPITCH Study). *Journal of hospital medicine* 11(1)**,** 39‐44. doi: 10.1002/jhm.2493.

Quintana-Bárcena, P., Lord, A., Lizotte, A., Berbiche, D., and Lalonde, L. (2018). Prevalence and Management of Drug-Related Problems in Chronic Kidney Disease Patients by Severity Level: a Subanalysis of a Cluster Randomized Controlled Trial in Community Pharmacies. *Journal of managed care & specialty pharmacy* 24(2)**,** 173‐181. doi: 10.18553/jmcp.2018.24.2.173.

Romskaug, R., Skovlund, E., Straand, J., Molden, E., Kersten, H., Pitkala, K.H., et al. (2020). Effect of Clinical Geriatric Assessments and Collaborative Medication Reviews by Geriatrician and Family Physician for Improving Health-Related Quality of Life in Home-Dwelling Older Patients Receiving Polypharmacy: a Cluster Randomized Clinical Trial. *JAMA internal medicine* 180(2)**,** 181‐189. doi: 10.1001/jamainternmed.2019.5096.

Schnipper, J.L., Samal, L., Nolido, N., Yoon, C., Dalal, A.K., Magny-Normilus, C., et al. (2021). The Effects of a Multifaceted Intervention to Improve Care Transitions Within an Accountable Care Organization: results of a Stepped-Wedge Cluster-Randomized Trial. *Journal of hospital medicine* 16(1)**,** 15‐22. doi: 10.12788/jhm.3513.

Sellors, J., Kaczorowski, J., Sellors, C., Dolovich, L., Woodward, C., Willan, A., et al. (2003). A randomized controlled trial of a pharmacist consultation program for family physicians and their elderly patients. *Cmaj* 169(1)**,** 17-22.

Sloeserwij, V.M., Hazen, A.C.M., Zwart, D.L.M., Leendertse, A.J., Poldervaart, J.M., de Bont, A.A., et al. (2019). Effects of non-dispensing pharmacists integrated in general practice on medication-related hospitalisations. *British journal of clinical pharmacology* 85(10)**,** 2321‐2331. doi: 10.1111/bcp.14041.

Song, Y.K., Jeong, S., Han, N., Na, H., Jang, H.Y., Sohn, M., et al. (2021). Effectiveness of clinical pharmacist service on drug-related problems and patient outcomes for hospitalized patients with chronic kidney disease: A randomized controlled trial. *Journal of Clinical Medicine* 10(8). doi: 10.3390/jcm10081788.

Tamblyn, R., Abrahamowicz, M., Buckeridge, D.L., Bustillo, M., Forster, A.J., Girard, N., et al. (2019). Effect of an Electronic Medication Reconciliation Intervention on Adverse Drug Events: A Cluster Randomized Trial. *JAMA Netw Open* 2(9)**,** e1910756. doi: 10.1001/jamanetworkopen.2019.10756.

Taylor, C.T., Byrd, D.C., and Krueger, K. (2003). Improving primary care in rural Alabama with a pharmacy initiative. *Am J Health Syst Pharm* 60(11)**,** 1123-1129. doi: 10.1093/ajhp/60.11.1123.

Touchette, D.R., Masica, A.L., Dolor, R.J., Schumock, G.T., Choi, Y.K., Kim, Y., et al. (2012). Safety-focused medication therapy management: a randomized controlled trial. *J Am Pharm Assoc (2003)* 52(5)**,** 603-612. doi: 10.1331/JAPhA.2012.12036.

Turakhia, M., Sundaram, V., Smith, S.N., Ding, V., Michael Ho, P., Kowey, P.R., et al. (2021). Efficacy of a centralized, blended electronic, and human intervention to improve direct oral anticoagulant adherence: Smartphones to improve rivaroxaban ADHEREnce in atrial fibrillation (SmartADHERE) a randomized clinical trial. *American Heart Journal* 237**,** 68-78. doi: 10.1016/j.ahj.2021.02.023.

Tuttle, K.R., Alicic, R.Z., Short, R.A., Neumiller, J.J., Gates, B.J., Daratha, K.B., et al. (2018). Medication Therapy Management after Hospitalization in CKD: A Randomized Clinical Trial. *Clin J Am Soc Nephrol* 13(2)**,** 231-241. doi: 10.2215/cjn.06790617.

Van der Linden, L., Decoutere, L., Walgraeve, K., Milisen, K., Flamaing, J., Spriet, I., et al. (2017). Combined Use of the Rationalization of Home Medication by an Adjusted STOPP in Older Patients (RASP) List and a Pharmacist-Led Medication Review in Very Old Inpatients: Impact on Quality of Prescribing and Clinical Outcome. *Drugs and Aging* 34(2)**,** 123-133. doi: 10.1007/s40266-016-0424-8.

Verdoorn, S., Kwint, H.F., Blom, J.W., Gussekloo, J., and Bouvy, M.L. (2019). Effects of a clinical medication review focused on personal goals, quality of life, and health problems in older persons with polypharmacy: a randomised controlled trial (DREAMeR-study). *PLoS medicine* 16(5)**,** e1002798. doi: 10.1371/journal.pmed.1002798.

Welch, E.K., Delate, T., Chester, E.A., and Stubbings, T. (2009). Assessment of the impact of medication therapy management delivered to home-based Medicare beneficiaries. *Ann Pharmacother* 43(4)**,** 603-610. doi: 10.1345/aph.1L524.

Westberg, S.M., Swanoski, M.T., Renier, C.M., and Gessert, C.E. (2014). Evaluation of the impact of comprehensive medication management services delivered posthospitalization on readmissions and emergency department visits. *Journal of managed care & specialty pharmacy* 20(9)**,** 886‐893. doi: 10.18553/jmcp.2014.20.9.886.

Wittayanukorn, S., Westrick, S.C., Hansen, R.A., Billor, N., Braxton-Lloyd, K., Fox, B.I., et al. (2013). Evaluation of medication therapy management services for patients with cardiovascular disease in a self-insured employer health plan. *J Manag Care Pharm* 19(5)**,** 385-395. doi: 10.18553/jmcp.2013.19.5.385.

Wu, W.C., Taveira, T.H., Jeffery, S., Jiang, L., Tokuda, L., Musial, J., et al. (2018). Costs and effectiveness of pharmacist-led group medical visits for type-2 diabetes: a multi-center randomized controlled trial. *PloS one* 13(4)**,** e0195898. doi: 10.1371/journal.pone.0195898.

Yin, D., Guo, Q., Geng, X., Song, Y., Song, J., Wang, S., et al. (2020). The effect of inpatient pharmaceutical care on nephrotic syndrome patients after discharge: a randomized controlled trial. *International journal of clinical pharmacy* 42(2)**,** 617‐624. doi: 10.1007/s11096-020-00975-9.

Zillich, A.J., Snyder, M.E., Frail, C.K., Lewis, J.L., Deshotels, D., Dunham, P., et al. (2014). A randomized, controlled pragmatic trial of telephonic medication therapy management to reduce hospitalization in home health patients. *Health services research* 49(5)**,** 1537‐1554. doi: 10.1111/1475-6773.12176.
